# Lost and Found: Western Honey Bee (*Apis mellifera*) *Matrix Metalloproteinase 1*

**DOI:** 10.1002/ece3.74600

**Published:** 2026-10-27

**Authors:** Jessica Braunisch, Ken Werner, Ulrich R. Ernst, Christian W. W. Pirk, Anja Buttstedt

**Affiliations:** ^1^ Institut für Evolution und Ökologie, Vergleichende Zoologie Eberhard Karls Universität Tübingen Tübingen Germany; ^2^ Institut für Evolution und Ökologie, Evolutionsökologie der Tiere Eberhard Karls Universität Tübingen Tübingen Germany; ^3^ Independent Researcher Ostfildern Germany; ^4^ Department of Zoology and Entomology, Social Insects Research Group University of Pretoria Pretoria South Africa

**Keywords:** alternative splicing, endopeptidase, extracellular matrix, matrix metallopeptidase, reference genome

## Abstract

The quality of reference genomes is of utmost importance as they and the therein annotated genes and proteins serve as basis for many downstream applications. Although the current Western honey bee ( *Apis mellifera* ) genome assembly (Amel_HAv3.1) represents a significant improvement over previous versions (Amel_4.0, Amel_4.5), it nevertheless lacks certain protein‐coding genes present in those earlier assemblies. One of these “lost and found” genes is locus 409868 of Amel_4.0 and Amel_4.5 which is missing due to an approximately 160 kb assembly gap on chromosome 10 in Amel_HAv3.1. Here we show that the *A. mellifera* genome indeed harbours locus 409868 encoding *matrix metalloproteinase 1* (*mmp1*) and that it is expressed in at least four different mRNA isoforms in honey bee workers. Orthologues from other insect species have essential tissue and organ remodelling functions during development and gene knockdowns at specific developmental time points are lethal. As adult honey bee workers show in addition an elaborate age‐based division of labour with drastic major morphological and physiological changes requiring extensive tissue and organ remodelling, leaving out *mmp1* because of a missing gene in the reference genome, may mean leaving out a potential key player in development.

## Graphical abstract

Despite considerable overall improvements in the Western honey bee ( *Apis mellifera* ) reference genome over the past two decades, some genes are missing in the current reference assembly (Amel_HAv3.1), potentially interfering with the meaningfulness of downstream analyses. One of these genes is *matrix metalloproteinase 1* (*mmp1*, locus 409868), which we show here to be indeed present in the genome and to be expressed in several isoforms. As *mmp1* is a crucial gene during development in other insect species, we recommend performing downstream analyses with the new (Amel_HAv3.1) and the old (Amel_4.5) reference assembly to not miss out on a crucial gene.

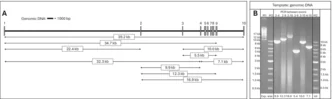

## 1 Introduction

Since the publication of the first insect genome (fruit fly— *Drosophila melanogaster* ) 26 years ago (Adams et al. 2000), insect species for which genomes are available are tremendously increasing within the NCBI and the InsectBase database (2015—138 species; 2021—601 and 815 species, respectively; 2024—2618 species; 2025—3020 species; Yin et al. 2016; Hotaling et al. 2021; Mei et al. 2022; Li et al. 2025; Sayers et al. 2025; Wang et al. 2026). This is amongst others facilitated by decreased sequencing costs and the rise of long‐read sequencing technologies boosting de novo assembly of genomes (routinely reads of more than 10 kb are generated, with the record for ultra‐long reads being pushed over 5 Mb) (Lu et al. 2024; Li et al. 2025). Long‐read assemblies tremendously increase insect genome qualities as they are more contiguous than short‐read assemblies with an average contig N50 value (shortest contig length that needs to be included to cover 50% of the genome (International Human Genome Sequencing Consortium 2001)) of 4.5 Mb compared to 94.4 kb and as a result of it, gene regions are more complete in long‐read assemblies compared to short‐read assemblies with fewer fragmented genes (Hotaling et al. 2021). Increased genome quality is of utmost importance as reference genomes and therein annotated genes serve as basis for many downstream applications such as transcriptome (e.g., Zhang et al. 2022; Mumoki et al. 2025) and proteome (e.g., McAfee et al. 2020; Chapman et al. 2025) analyses.

The first Western honey bee ( *Apis mellifera* ) genome (Amel_4.0), published in 2006, was based on whole‐genome shot‐gun sequencing with Sanger technology (Honeybee Genome Sequencing Consortium 2006). In 2014, an improved assembly (Amel_4.5) was published, additionally using ABI SOLiD and Roche 454 paired‐end sequencing technologies to superscaffold and fill gaps in the old assembly (Elsik et al. 2014). This slightly increased the contig N50 value from 40.5 to 45.7 kb and led to the annotation of newly discovered genes (Elsik et al. 2014) with 9137 and 10,746 protein coding genes being annotated for Amel_4.0 (https://www.ncbi.nlm.nih.gov/datasets/gene/GCF_000002195.3/?gene_type=protein‐coding) and Amel_4.5 (https://www.ncbi.nlm.nih.gov/datasets/gene/GCF_000002195.4/?gene_type=protein‐coding), respectively (Honeybee Genome Sequencing Consortium 2006; Elsik et al. 2014). In 2019, a new de novo *A. mellifera* genome assembly (Amel_HAv3.1) was published, combining data from four genome sequencing and mapping technologies (PacBio SMRT sequencing, 10× chromium linked‐read sequencing, BioNano Genomics Irys optical mapping, Hi‐C chromatin interaction map) (Wallberg et al. 2019). This led to a more contiguous and complete genome as additional sequence could be incorporated into scaffolds and the numbers of gaps could be reduced, increasing the contig N50 value to 5.4 Mb (Wallberg et al. 2019). However, despite of the overall much improved assembly, 6.6 Mb of chromosomal sequence in Amel_4.5 could not be matched in the new reference genome (Wallberg et al. 2019). Assembly completeness was assessed by counting the number of universal single‐copy orthologues (genes that can be found in all genomes within the taxon but have remained in a single copy since the last common ancestor (Creevey et al. 2011; Waterhouse et al. 2011)). It was found that Amel_HAv3.1 had a slightly larger number of Hymenopteran universal single‐copy orthologues genes compared to Amel_4.5 (4337 genes and 4310 genes, respectively) with fewer genes occurring on unplaced contigs (Wallberg et al. 2019). Yet, the number of annotated genes coding for proteins dropped by approximately 800 genes to 9935 (https://www.ncbi.nlm.nih.gov/datasets/gene/GCF_003254395.2/?gene_type=protein‐coding).

One of the protein‐coding genes that is present in Amel_4.0 and Amel_4.5 but missing in the current reference genome (Amel_HAv3.1) is a *matrix‐metalloproteinase* (*mmp*) gene (LOC409868, named ‘*similar to matrix metalloproteinase 1*’ in Amel_4.0 and ‘*matrix metalloproteinase‐14*’ in Amel_4.5), which caught our attention as the corresponding protein was found during a mass spectrometric study identifying proteins in Western honey bee queen larval food (identified with 8 unique peptides, 16% coverage) (Zhang et al. 2014). In the old genome (Amel_4.5), locus 409868 is located on linkage group 10 (NC_007079.3) between loci 409869 (kinesin 3A) and 410006 (uncharacterized LOC410006). The flanking loci can also be found in the current reference genome (linkage group 10: NC_037647.1), but locus 409868 comprising the *mmp* is missing due to a 158,704 bp assembly gap between bases 4,204,752 and 4,046,047.

*Mmps* are best known and studied in humans where 23 different *mmps* are expressed which degrade diverse proteins in the extracellular matrix (Gersh and Catchpole 1949; Laronha and Caldeira 2020). In insects (e.g., *Anopheles gambiae* , *Bombyx mori* , *Tribolium castaneum* ), three genes encoding *matrix metalloproteinases* (*mmp1*, *mmp2* & *mmp3*) are described (Holt et al. 2002; Knorr et al. 2009; Goulielmaki et al. 2014; Jia et al. 2018; Liu et al. 2022), with *Drosophila melanogaster* missing *mmp3* (Page‐McCaw et al. 2003). Most *mmp* genes are expressed in alternative splice variants, resulting in distinct protein isoforms whose main difference is that they are either secreted or stay attached to the cell membrane via a membrane spanning transmembrane domain or a glycosylphosphatidylinositol anchor (Goulielmaki et al. 2014; LaFever et al. 2017). In general, also insect matrix metalloproteinases (MMPs) regulate extracellular matrix remodelling of tissues and organs which is amongst others crucial during metamorphosis (Page‐McCaw et al. 2003; Glasheen et al. 2010; Bond et al. 2011; Jia et al. 2018; Wen et al. 2020; Liu et al. 2022; for review see Page‐McCaw et al. 2007). Furthermore, insect MMPs have functions in innate immunity and wound healing (Knorr et al. 2009; Mitten et al. 2012; Stevens and Page‐McCaw 2012; Goulielmaki et al. 2014; Liu et al. 2022; for review see Page‐McCaw et al. 2007). In *D. melanogaster* both *mmp* genes are required for viability (Page‐McCaw et al. 2003) and in *T. castaneum* knock‐down of *mmp1* leads to developmental arrest and death during early metamorphosis (Knorr et al. 2009). Thus, MMPs likely have similar crucial functions in *A. mellifera* that are difficult to identify when the gene and thus also mRNA and protein reference sequences are missing from the reference genome.

We here show using classical PCR and old‐style Sanger sequencing that locus 409868, missing in the current *A. mellifera* reference genome (Amel_HAv3.1), contains a gene that is comprised of 10 exons. Phylogenetic analyses identified the gene as a *matrix metalloproteinase 1* (*mmp1*) orthologue and we found evidence for the expression of four different mRNA isoforms by the combination of shorter or longer forms of exon 9 and 10, respectively. In addition, the genome of *A. mellifera* harbours one more gene encoding a matrix metalloproteinase, being an ortholog of *mmp2* (LOC409383) and a much‐shortened variant of *mmp3* (LOC102655811) which is likely non‐functional as MMP.

## 2 Results

### 2.1 Amplification of *mmp_LOC409868* From DNA to Confirm Gene Presence

Amplification was successful resulting in PCR products of the expected sizes (Figure 1A) between potential exon 2 and 4 (expected: 9.9 kb, determined: 10.2 ± 0.2 kb [mean ± standard deviation]), 2 and 9 (expected: 12.3 kb, determined: 12.0 ± 0.7 kb), 2 and 10 (expected: 16.9 kb, determined: 14.8 ± 1.8 kb), 3 and 9 (expected: 5.5 kb, determined: 5.4 ± 0.1 kb), 3 and 10 (expected: 10.0 kb, determined: 10.0 ± 0.7 kb) as well as 4 and 10 (expected: 7.1 kb, determined: 7.1 ± 0.3 kb) (Figure 1B).

**FIGURE 1:**
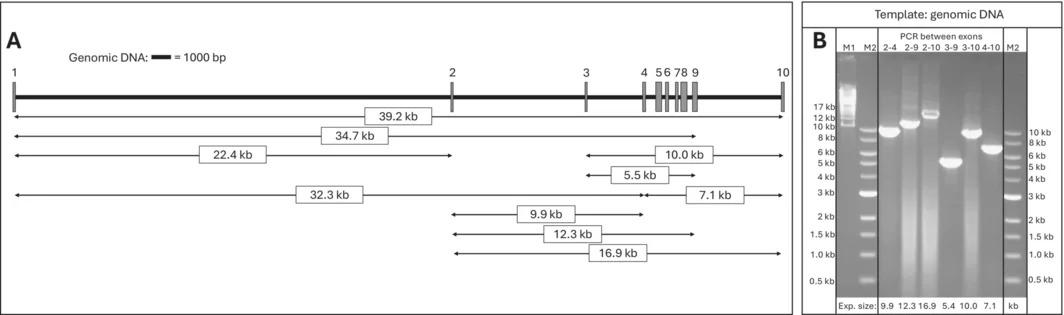
(A) Potential structure of the LOC409868 gene (genomic DNA). Grey boxes represent 10 potential exons, black lines introns. Expected PCR product sizes are given in white boxes. Expected exons and sizes are based on manual annotation of mRNA HP491739.1 (Elsik et al. 2014) on *Apis mellifera* isolate Amel_TP_3 contig_39 (JBGNXS010000264.1; BioProject PRJNA1076921; Gilligan et al. 2024, bioRxiv); see ‘Primer design’ in Material & Methods. (B) Agarose gel (0.5%, TAE buffer, 50 V, 5 h) showing long‐range PCR products of *mmp_LOC409868* amplification from genomic PCR. M1, GeneRuler High Range DNA ladder; M2, 1 kb DNA ladder.

Beginning from exon 1, none of the products could be amplified likely due to their large, expected sizes (22.4–39.2 kb) (Figure 1A). The genomic region between the primers in exon 2 and 10 (10.0 kb) was largely sequenced using Sanger sequencing (Data S1, Figure S1). It was not possible to neither fully sequence intron 2 (4.8 of 6.7 kb missing) nor intron 9 (1.6 of 3.9 kb missing), as overlaying sequence was observed after repetitive bases. This was likely due to the use of a diploid worker and different numbers of repeats between the homologous chromosomes. As the PCR products matched the expected sizes and the Sanger sequencing otherwise confirmed the sequence published within the whole genome shotgun contig JBGNXS010000264.1, we filled the gaps in intron 2 and 9 of the Sanger sequence with Ns according to the missing length based on JBGNXS010000264.1 and uploaded the sequence to NCBI (GenBank ID: PX921700).

Blasting (NCBI, blastn) this sequence (PX921700) against the Amel_4.5 assembly (GCF_000002195.4) resulted in two hits. The first was against bases 4,087,902 to 4,104,809 of chromosome 10 comprising LOC409868 (NC_007079.3, 99.92% identity, *E* value 0.0) and the second against an only 961 bp unplaced genomic scaffold (NW_003379696.1, 99.63% identity, *E* value 6e‐138) which aligns perfectly to bases 4,087,271 to 4,088,174 of chromosome 10, thus to the 3′ end of LOC409868. Blasting against the Amel_HAv3.1 assembly (GCF_003254395.2) did not result in any hits with significant similarity. However, blasting against the long‐read raw data of Amel_HAv3.1 uploaded to the sequence read archive (SRA) database (SRX6817383, Pacific Biosciences SMRT sequencing) resulted in the identification of 139 aligning sequences (> 81.8% identity, *E* values 0 to 7e‐42) (Table S1).

### 2.2 Amplification of *mmp_LOC409868* From cDNA to Confirm Gene Expression

Amplification from 30 ng cDNA was successful from all bees resulting in the expected PCR product sizes between exon 1 and 4 (expected: 456 bp, determined: 417 ± 17 bp), 2 and 4 (expected: 335 bp, determined: 306 ± 19 bp), 6 and 9 (expected: 645 bp, determined: 633 ± 10 bp) as well as 3 and 9 (expected: 1160 bp, determined: 1202 ± 41 bp) (Figure 2B,C). In forager bees, expression was comparable when RNA was isolated from heads and abdomens but using cDNA from thorax‐RNA as template only resulted in very faint bands (Figure 2B, Figure S2). In nurse bees, expression was also detected in the thoraces (Figure 2C, Figure S2). Even though reverse transcription (RT)‐PCR analysed with agarose gels is only semi‐quantitative, it certainly shows that the gene is expressed. Sanger sequencing of the PCR products confirmed all expected exon/intron boundaries for exon 1–9 (Data S1, Figure S3).

**FIGURE 2:**
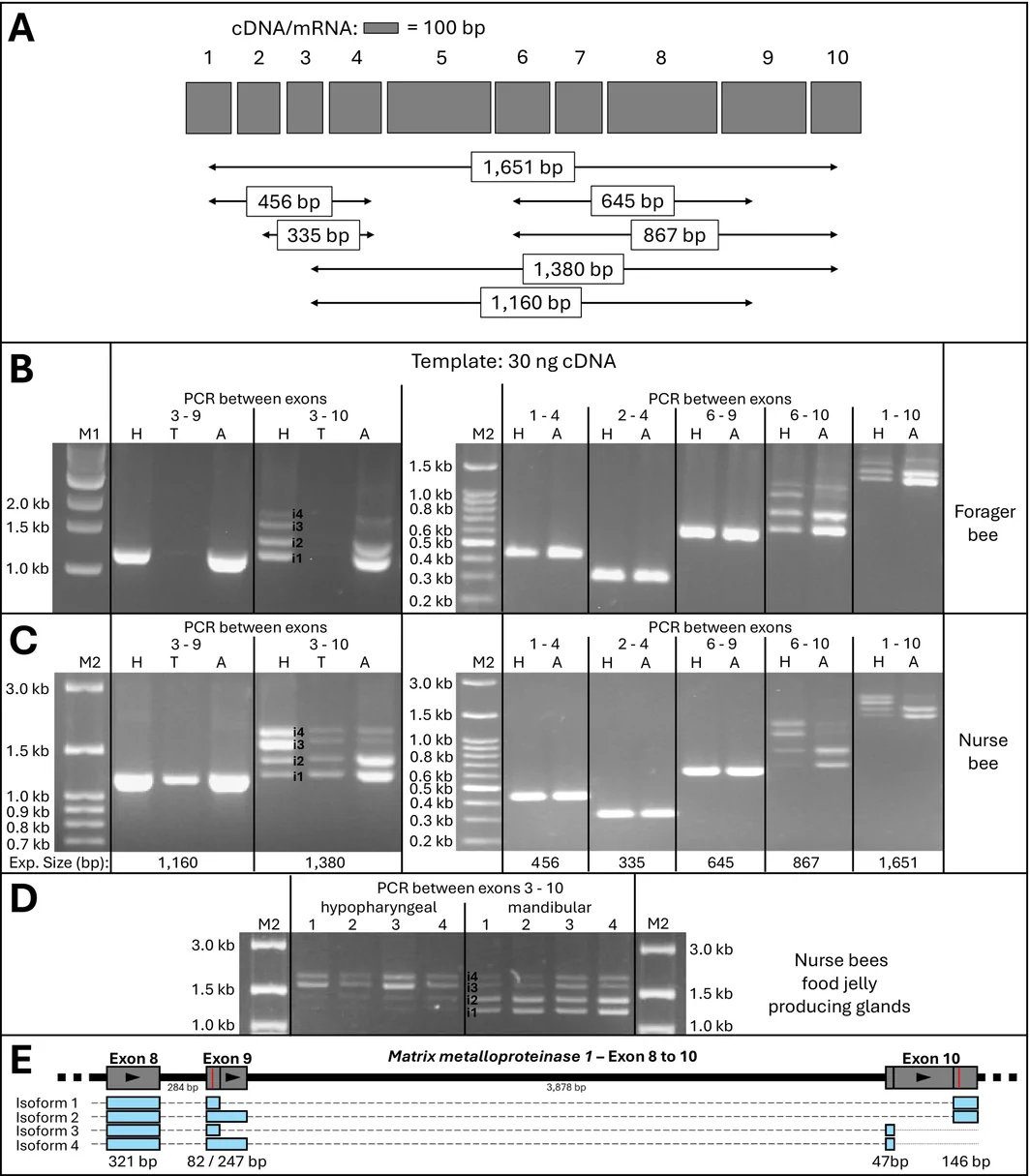
(A) Potential structure of the coding mRNA sequence (cDNA/mRNA) of the LOC409868 gene. Grey boxes represent 10 potential exons, expected PCR product sizes are given in white boxes. (B–D) Agarose gels (1.5%, TBE buffer, 100 V, 2 h) showing PCR products of *mmp_LOC409868* amplification from cDNA (RT‐PCR) of an individual forager (B), an individual nurse bee (C) and hypopharyngeal as well as mandibular glands (D) isolated from four nurse bees. M1, 1 kb DNA ladder (NEB); M2, 100 bp plus DNA ladder (NEB); H, head; T, thorax; A, abdomen; i1‐i4, isoforms 1–4. For gels of PCR products from all bees please see Figure S2. (E) Schematic representation of *mmp1* genomic DNA from exon 8–10 (boxes in grey) showing the different mRNA isoforms (boxes in light blue) produced by alternative splicing. Exon 1–7 are not shown as alternative splicing was not detected between these exons. The binding sites of the revers primers in exon 9 and 10 are indicated by red lines. In isoform 3 and 4, the PCR products, when using the revers primer in exon 10, include 388 bases 3′ untranslated region (dotted line) after the stop codon. Thus, PCR products of isoform 3 and 4 are bigger than those of isoform 1 and 2 even though the coding sequence is shorter.

If amplification included potential exon 10, the RT‐PCR resulted in three to four PCR products (exon 1–10, expected: 1651 bp, determined: 1637 ± 36/1831 ± 47/2096 ± 72/2267 ± 68 bp; exon 3–10, expected: 1380 bp, determined: 1274 ± 51/1463 ± 74/1774 ± 86/1964 ± 101 bp; exon 6–10, expected: 867 bp, determined: 699 ± 14/902 ± 11/1209 ± 21/1429 ± 27 bp) (Figure 2B–D, Figure S2). This pointed to potential alternative splicing as had been described for *mmps* from other insects (Goulielmaki et al. 2014; LaFever et al. 2017) between potential exon 9 and 10. Using individual PCR products after gel elution as template in subsequent PCRs (templates: 1—exon 3–10 PCR, 1240 bp band; 2—exon 3–10 PCR, 1410 bp band; 3—exon 6–10 PCR, 1220 bp band; 4—exon 6–10 PCR, 1440 bp band) resulted in single bands which were sent for sequencing and confirmed the expression of four splice variants (i1 to i4, Figure 2B–D, Figure S3). The four splice variants were annotated on the GenBank sequence (PX921700). The variants contain either a shorter (isoform 1 and 3) or longer exon 9 (isoform 2 and 4) (82 or 247 bp & 27 or 82 amino acids, respectively). Furthermore, two different variants of exon 10 are transcribed (exon 10a—isoform 3 and 4 & exon 10b—isoform 1 and 2), one comprising 47 bp protein‐coding sequence including the stop codon and the second one 146 bp. There seem to be differences in *mmp1* expression patterns between body parts and castes (Figure 2B,C, Figure S2), however, the observed difference is based on a rather small sample size. Analysing food jelly producing glands (Figure 2D), that is, hypopharyngeal and mandibular glands, showed again that the gene is expressed and that there are potential differences in isoform diversity with hypopharyngeal glands primarily expressing isoform 3 and 4.

### 2.3 Analysis of Proteins

The resulting protein isoforms (Table S2) consist of 520, 553, 575 or 608 amino acids (with position 1–505 being the same), respectively and are all predicted to have a 30 amino acids long N‐terminal signal peptide targeting the proteins to the endoplasmic reticulum. All isoforms are predicted to be extracellular zinc‐dependent and calcium‐activated metalloproteases (InterPro: peptidase M10A protein family, IPR021190; I‐TASSER: metalloendopeptidase activity, GO‐score 0.61–0.77 / zinc ion binding, GO‐score 0.61–0.77 / calcium ion binding GO‐score 0.50–0.53/best PDB hits, human MMP1 (1SU3) and MMP2 (1CK7)) that contain a hemopexin‐like domain in their C‐terminal sections, which is thought to facilitate binding to other proteins (InterPro: IPR021190, position 303–498; I‐TASSER: protein binding, GO‐score 0.41) (for review see (Das et al. 2003)). All isoforms contain the HEXXHXXGXXH motif (HEFGHSLGLSH, position 232–242), being the active site for zinc binding (for review see Rawlings and Barrett 1995) and are synthesised as inactive precursors with pro‐domains harbouring a conserved motif, PRCGV (position 99–103), the matrix cysteine switch, in which the cysteine interacts with the essential zinc ion to prevent it from binding the catalytic water molecule, maintaining the precursor inactive (for review see Rawlings and Barrett 1995). All isoforms are predicted to have two N‐linked glycosylations (NetNGlyc: position 57 and 83). The additional 165 bp of exon 9 in isoform 2 and 4 encode an amino acid stretch with an additional glycosylation site (NetNGlyc: position 507), two additional cysteines separated by only four other amino acids (CXXXXC) and very low pI (3.44) as 16.7% of amino acids are glutamate or aspartate. Exon 10b encodes a C‐terminal transmembrane region (ILWYLPLNLMTIFIVKIVA, InterPro). Thus, isoforms 1 and 2 are predicted to be attached to the membrane (endoplasmic reticulum, Golgi apparatus or plasma membrane) with the protein being exposed to the lumen or extracellular space, while isoforms 3 and 4 are likely directly secreted from the cell.

### 2.4 Identification of Transcripts Within the Sequence Read Archive

The following available transcriptomes were screened for the expression of *mmp1* (isoform 2) (Data S2): (1) A comprehensive expression dataset for *A. mellifera* worker development from larva to adult (day 6–58 post‐oviposition, PRJDB14753 (Yokoi et al. 2025)). (2) Transcriptomes of larval and adult worker hemocytes (8 day old larvae, freshly emerged adults, PRJNA1247573 (Braeckman et al. 2025)) as MMPs have been shown to be involved in innate immunity in other insect species (Knorr et al. 2009; Xu et al. 2012; Liu et al. 2022). (3) Transcriptomes of hypopharyngeal (HPG) and mandibular glands (MDG) of nurse and forager bees (PRJNA243651 (Jasper et al. 2015)) as those glands produce food jelly in which MMP1 was already identified on protein level (Zhang et al. 2014). (4) Transcriptomes of worker and queen larval ovaries (PRJNA636804 (Lago et al. 2022)). We found in all datasets sequences producing significant alignments to *mmp1* (Data S2) with more sequences aligning in cDNA from adult worker hemocytes (307 sequences) than from larval ones (46 sequences). *Mmp1* is not listed as a gene that is expressed in hemocytes in the original publication as the alignment was performed against Amel_HAv3.1 (Braeckman et al. 2025). In MDGs and HPGs a continuously high number of aligning sequences is found (nurse MDGs: 4718 ± 362, forager MDGs: 2388 ± 1300, nurse HPGs: 4803 ± 186, forager HPGs: > 5000) and throughout honey bee development the number of aligning sequences peaks shortly before or directly at developmental transitions (day 13—larva to prepupa transition, day 15—prepupa to pupa, day 22—pupa to adult, day 38—adult nurse to forager transition; Data S2, Figure S4). Furthermore, sequences producing significant alignments were found in worker and queen larval ovary transcriptomes (worker and queen larval ovaries: 1574 and 1816 sequences, respectively).

### 2.5 Phylogeny

Blasting of MMPs from other insect species against the Amel_4.5 and Amel_HAv3.1 genome annotation resulted in hits against the here studied gene LOC409868 (only Amel_4.5) and further identified LOC409383 (Amel_4.5 & Amel_HAv3.1) as well as LOC102655811 (Amel_4.5 & Amel_HAv3.1) as potential *mmps* (Furthermore, the 961 bp long unplaced genomic scaffold (NW_003379696.1, Amel_4.5) already described earlier as aligning to LOC409868 was identified as well). LOC409383 is currently annotated as *matrix metalloproteinase 2* coding for four different protein isoforms and LOC102655811 as *matrix metalloproteinase 24‐like* with two protein isoforms.

Phylogenetic analysis including the potential *A. mellifera* MMPs as well as MMPs from *Homo sapiens* , *D. melanogaster* , *T. castaneum* , *A. gambiae* , and *B. mori* revealed that MMP_LOC409868 is an ortholog of insect MMP1 (Figure 3). LOC409383 protein isoforms clustered to MMP2 of other insect species and LOC102655811 isoforms to MMP3 (Figure 3). MMP2 and MMP3 form a monophyletic group which is more closely related to different human MMPs than to insect MMP1, pointing to an ancient divergence in the *mmp* gene family (Figure 3). As for *A. mellifera* MMP1, all *A. mellifera* MMP2 isoforms were identified as members of the peptidase M10A protein family (InterPro: IPR021190) containing a signal peptide, the HEXXHXXGXXH (zinc binding), and PRCGV (matrix cysteine switch) motifs as well as the hemopexin‐like domain (InterPro: IPR021190). Neither of the two *A. mellifera* LOC102655811 isoforms that clustered to MMP3 was identified as such. Furthermore, while *T. castaneum* , *A. gambiae* , and *B. mori* MMP3s are proteins of 572, 582, and 510 amino acids, the two isoforms in *A. mellifera* consist of only 99 and 117 amino acids, respectively. Isoform 1 contains none of the typical motifs while isoform 2 still has the matrix cysteine switch (position 91–95) but is missing the zinc binding motif and the hemopexin‐like domain.

**FIGURE 3:**
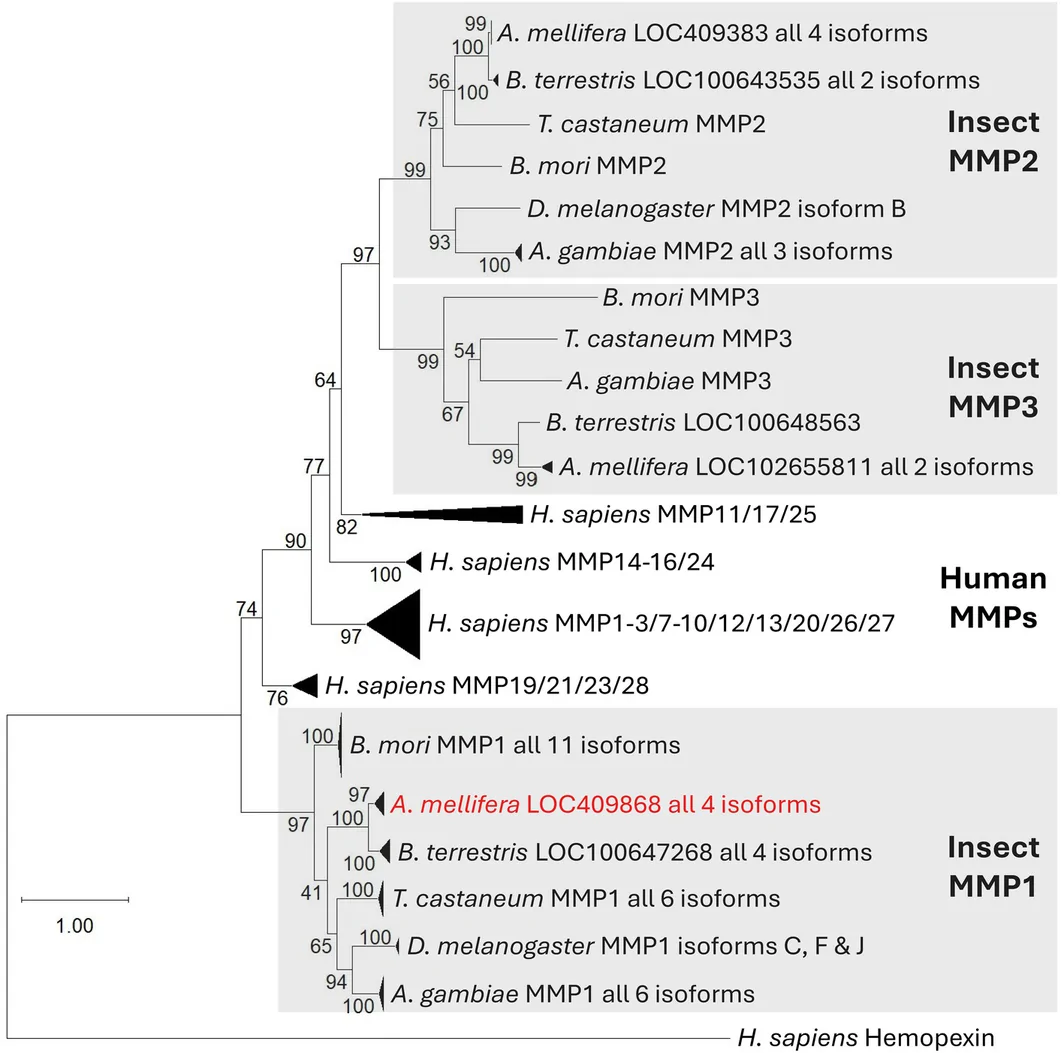
The phylogeny of MMPs was inferred using the Maximum Likelihood method and the Whelan and Goldman (2001) model (+Freq) of amino acid substitutions and the tree with the highest log likelihood (−41.229.90) is shown. The percentage of replicate trees in which the associated taxa clustered together (500 replicates) is shown next to the branches (Felsenstein 1985). The initial tree for the heuristic search was selected by choosing the tree with the superior log‐likelihood between a Neighbour‐Joining (NJ) tree (Saitou and Nei 1987) and a Maximum Parsimony (MP) tree. The evolutionary rate differences amongst sites were modelled using a discrete Gamma distribution across 5 categories (+G, parameter = 1.0959), with 0.39% of sites deemed evolutionarily invariant (+I). The analytical procedure encompassed 76 amino acid sequences with 1.293 positions in the final dataset. Evolutionary analyses were conducted in MEGA (Kumar et al. 2024). Protein isoforms of the same gene always clustered together and isoform subtrees as well as subtrees only harbouring some human MMPs were compressed. For accession numbers of protein sequences please see (Table S2).

Furthermore, protein isoforms encoded by the identified *mmp* loci in *B. terrestris* (*mmp1*—LOC100647268, 4 protein isoforms; *mmp2*—LOC100643535, 2 protein isoforms and *mmp3*—LOC100648563, 1 protein) always clustered to the respective orthologs (Figure 3). As in *A. mellifera* , the protein that clustered to MMP3 consists of only 124 amino acids, is not identified as M10A peptidase, does neither contain the zinc binding motif nor the hemopexin‐like domain but the matrix cysteine switch (position 91–95). Thus, *A. mellifera* and *B. terrestris* *mmp3* encode for largely shortened MMP3 proteins, only consisting of signal peptide and (partial) pro‐domain but missing the actual protease and the hemopexin‐like domain.

### 2.6 BUSCO Analysis

Being now aware of the fact that genes, like *mmp1*, may be missing in the Amel_HAv3.1 assembly, even though they have been present in Amel_4.5, we reanalysed both genomes with BUSCO (Simão et al. 2015) and assessed universal single‐copy orthologues in both genome assemblies as the number of eukaryotic genomes in the database increased 4.6‐fold in the updated version 12 compared to version 10 in 2018 (Kriventseva et al. 2019; Tegenfeldt et al. 2025). As published before (Wallberg et al. 2019), Amel_HAv3.1 has a slightly higher number of Metazoan and Hymenopteran BUSCO genes than Amel_4.5 and reversely a lower number of missing BUSCO genes (Table 1, Tables S3–S7). However, when comparing the identity of the missing BUSCO genes (Table S3), it became clear that the fewer missing BUSCOs in Amel_HAv3.1 were not entirely due to additional genes newly identified in Amel_HAv3.1. Instead, there were 15 genes that have been present in the Amel_4.5 assembly but are now reported by BUSCO as missing in Amel_HAv3.1 (Table 1, Supporting Information Results).

**TABLE 1:** Analysis of BUSCO genes in Amel_4.5 and Amel_HAv3.1.

	Metazoa (*n* = 672)	Hymenoptera (*n* = 5920)
Present BUSCOs		
In Amel_4.5	644 (95.8%)	5755 (97.2%)
In Amel_HAv3.1	661 (98.4%)	5877 (99.3%)
Missing BUSCOs		
Missing in Amel_4.5 & _HAv3.1	7	18
Missing only in Amel_4.5	8	66
Missing only in Amel_HAv3.1	2	15

Of those 15 genes (two Metazoan & Hymenopteran BUSCO genes, 13 Hymenopteran BUSCO genes), eight, including the two Metazoan BUSCO genes, were indeed completely missing even though their flanking loci could be found (Table S3, Supporting Information Results) and three were partially missing. The two missing Metazoan BUSCO genes are an O‐glucosyltransferase (Amel_4.5, LOC100577328) which glycosylates in *D. melanogaster* epidermal growth factor repeats in several proteins (Yu et al. 2016) and mitochondrial ribosomal protein L16 (Amel_4.5, LOC408404). Surprisingly, four of the BUSCO‐reported missing genes were fully present in Amel_HAv3.1 including their flanking loci at the expected location (Table S3, Supporting Information Results). A *matrix metalloproteinase* is listed amongst the Hymenopteran BUSCO genes (Hymenoptera Busco ID: 138194at7399) and LOC409868 encoding *mmp1*, is the by BUSCO found *mmp* in Amel_4.5. However, the gene is not reported as missing in Amel_HAv3.1 as BUSCO refers to *mmp2* (NC_037638.1; 5,521,822–5,493,218) as the *mmp* BUSCO gene in Amel_HAv3.1 even though it has the status ‘fragmented’.

## 3 Discussion

Despite the extraordinary improvements in genome quality over the past decades (Hotaling et al. 2021; Lu et al. 2024; Li et al. 2025), genome assemblies are certainly not free of errors and continued efforts are needed to further improve their quality, especially for reference genomes that are routinely used in downstream analyses. In the current *A. mellifera* reference genome assembly (Amel_HAv3.1) (Wallberg et al. 2019), even though in general more complete, 6.6 Mb of chromosomal sequence of the old assembly (Amel_4.5) could not be matched and seem to have been totally excluded from the new reference even though this sequence contained sometimes complete genes, like locus 409868, for which there was evidence of expression on protein level (Zhang et al. 2014). Furthermore, Amel_HAv3.1 has 51 unsolved genomic gaps of up to 345,148 bp (Kaskinova et al. 2021). Kaskinova et al. (2021) aimed on closing these gaps using de novo re‐assemblies of the Amel_HAv3.1 raw reads and other long‐read‐based assemblies and succeeded to resolve 13 gaps including the 158,704 bp gap on chromosome 10 comprising locus 409868. However, this was never implemented into the NCBI reference assembly where locus 409868 (amongst others) is still missing. We showed here that locus 409868 contains a gene that encodes *matrix metalloprotease 1* which is comprised of 10 exons and that is expressed in adult worker bees in at least four different alternative splice variants which translate into four different protein isoforms with differing C‐termini. This is similar to other insects, like *D. melanogaster* , *T. castaneum* , *A. gambiae* and *B. mori* , where usually more (up to 11) isoforms are predicted by automatic annotation than there is evidence for actual transcription (up to three) (Page‐McCaw et al. 2003; Knorr et al. 2009; Goulielmaki et al. 2014; Jia et al. 2018). MMP1 is crucial during development to remodel several tissues in larvae and pupae. In *D. melanogaster* it is required for normal tracheal growth and for cuticle degradation into smaller pieces for moulting (Page‐McCaw et al. 2003; Glasheen et al. 2010) and it has been shown to digest several collagens of the extracellular matrix (Wen et al. 2020). In *T. castaneum* last instar larvae, knock‐down of *mmp1* leads to developmental arrest and death during early metamorphosis, while when knocked‐down in pupae, individuals developed into normal adults (Knorr et al. 2009). In *B. mori* *mmp1* expression is highest during the first 2 days of pupation (Liu et al. 2022). The protein regulates tracheal tissue development (Wei et al. 2021), as in *D. melanogaster* , and its inhibition delays fat body cell dissociation and hampers ovary development in female pupae (Jia et al. 2018). Contrary, in *A. mellifera* , *mmp1* was not identified as one of the top 30 genes with differential expression between queen & worker larval ovaries (Lago et al. 2022). However, elucidating protein activity remains notoriously challenging when multiple isoforms are present, especially when activation requires removal of inhibitory pro‐domains. As worker ovaries undergo programmed cell death in the late larval stage rendering the resulting adult workers mostly sterile (Capella and Hartfelder 1998), it should be investigated in future studies whether MMP1 (or MMP2) is involved in ovary tissue remodelling. Using the *A. mellifera* worker development expression dataset (PRJDB14753 (Yokoi et al. 2025)) we showed that in *A. mellifera* the number of sequences aligning to *mmp1* peaks shortly before or directly at developmental transitions making an involvement in tissue remodelling during metamorphosis likely. Although this seems plausible, based on comparative studies in other insects, there are yet no data to support this hypothesis in *A. mellifera* . Furthermore, number of aligning sequences also peak in adulthood at the transition from nurse bee to forager during which worker honey bees cease feeding larvae inside the hive with food jelly and start foraging (Seeley 1982). This is accompanied by major morphological and physiological changes in food jelly producing glands, HPGs and MDGs, located within the workers heads (Soudek 1927; Boch and Shearer 1966; Dobritzsch et al. 2019; Langlands et al. 2022; Buttstedt et al. 2023; Yusuf et al. 2024). We found large numbers of sequences aligning to *mmp1* within an RNAseq dataset (Jasper et al. 2015) on MDGs and HPGs in nurse bees and foragers. The four *mmp1* isoforms identified by us were found in HPGs and MDGs of nurse bees (Figure 2D). This may indicate an involvement of one or several MMP1 isoforms in morphological changes of HPGs and MDGs during honey bee worker ageing. MMP1 peptides identified in food jelly (Zhang et al. 2014) are all within the shared part of the isoforms and thus it is not possible to clarify which isoforms are secreted into honey bee food jelly. However, nurse bee HPGs seem to primarily express isoform 3 and 4, the soluble isoforms without the C‐terminal transmembrane region. The presence of MMP1 in larval food jelly is certainly interesting as this opens up a possibility for an involvement of worker secreted MMP1 in larval tissue remodelling. In mice, matrix metalloproteinase 7 (MMP7) activates the antimicrobial peptide α‐pro‐defensin via proteolytic removal of the inhibitory pro‐domain (Wilson et al. 1999). Defensins are expressed for host defence in a mulitude of plants, animals and fungi (Shafee et al. 2017) and also *A. mellifera* expresses defensin as inactive pre‐cursor (Casteels‐Josson et al. 1994) with the mature defensin being amongst others identified in honey bee food jelly (Fujiwara et al. 1990) while the processing protease is not yet identified. Whether or not it may be MMP1 remains to be clarified. Furthermore, food jelly producing glands are not the only glands with drastic major morphological and physiological changes as honey bee workers show an elaborate division of labour by age (Seeley 1982) with a multitude of glands (e.g., wax, tergal, Nasonov, venom gland) that develop, function and deteriorate, sometimes at rather specific time points, dependent on age and colony state (Boch and Shearer 1963; Owen and Bridges 1976; Hepburn et al. 1991; Okosun et al. 2015; for review see Cassier and Lensky 1997).

MMP1 plays a role in wound healing in *T. castaneum* (Mitten et al. 2012) and *D. melanogaster* as it promotes basement membrane repair (Stevens and Page‐McCaw 2012). In *T. castaneum* the different MMPs seem to be at least partially redundant during wound healing as knock‐down of single *mmps* leads only to a delay while knock‐down of *mmps* in pairs inhibits wound closure (Mitten et al. 2012). Furthermore, MMP1 seems to be involved in insect innate immunity as its expression is increased in *T. castaneum* hemocytes of adults after septic injury and larvae injected with a fungal entomopathogen died significantly faster after *mmp1* knock‐down compared to controls (Knorr et al. 2009). Comparatively, in *A. mellifera* hemocytes *mmp1* expression was identified by us within the dataset published by Braeckman et al. (2025). In *A. gambia mmp1* expression increases 24 h after a meal on blood infected with *Plasmodium berghei* but not after feeding on uninfected blood (Goulielmaki et al. 2014). In *B. mori* larvae as well as in *Drosophila* cells, *mmp1* is upregulated after viral infection (Xu et al. 2012; Liu et al. 2022). Similarly, *mmp1* was upregulated in brains of *A. mellifera* workers 13 days post‐infection with black queen cell virus (reads aligned to Amel_4.5, table S2 in Doublet et al. (2016)) and in fat bodies of workers 20–24 h after being fed with extracts of virus‐infected bees (contained IAPV, DWV, BQCV, KBV and SBV) (reads aligned to Amel_4.5, table S1A in Galbraith et al. (2015)). However, *mmp1* did not show differential expression in workers within 72 h after infection with a model virus (reads aligned to Amel_4.5, table S5 in Brutscher et al. (2017)) and in a comparative analysis across studies (table S3 in Doublet et al. (2017)) *mmp1* was identified as one of 179 genes with significant differential expression, that is, being up‐regulated in some and down‐regulated in other studies, in response to multiple pathogens.

Thus, all in all, this points to important functions of MMP1 also in *A. mellifera* and missing such a gene would likely lead to misinterpretations or missing links especially in transcriptome or proteome studies on development and immunity. For example, Zhang et al. (2022) looked for caste‐differentiation‐related factors in honeybee brains and identified *mmp2* as differentially expressed between queen and worker brains. However, as the study is based on the Amel_HAv3.1 annotation, a potential differential expression of *mmp1* is overlooked as the gene is not present in this assembly. If it is not controlled in newly annotated reference genomes, whether genes are missing that have been present in earlier versions, all downstream applications should be performed against the two most recent genome annotations, and the results should be compared.

## 4 Material and Methods

### 4.1 Sample Collection, DNA, RNA Extraction and cDNA Synthesis

Forager honey bees were collected in August 2025 from blossoms in the botanical garden of the University of Tübingen (Hartmeyerstraße 123, 72076 Tübingen, Germany). Nurse bees were sampled in April 2026 from a queen‐right brood‐rearing colony located in 73760 Ostfildern, Germany. To raise worker bees of the same age, newly emerged bees were paint‐marked using Maxx 270 paint markers (Schneider, Germany) on their thoraces and returned to the hive. Marked workers were sampled on day 11. Total DNA was extracted from the thorax of one bee using the Wizard Genomic DNA Purification Kit (Promega, Mannheim, Germany). Total RNA was extracted from the head, thorax, and abdomen of four forager and four nurse bees, as well as from dissected hypopharyngeal (HPGs) and mandibular glands (MDGs) of four more nurse bees using the RNeasy MiniKit (Qiagen, Hilden, Germany) according to the manufacturer's protocol. From heads, thoraces, abdomen, and HPGs, 500 ng total RNA were reverse transcribed using 0.4 μg Oligo (dT)15 primer, 0.8 μL dNTPs (10 mM) and 80 U M‐MLV reverse transcriptase (all Promega). MDGs are known to contain only low amounts of RNA in nurse bees (Yusuf et al. 2024) and thus the total RNA amount (170–380 ng; HPGs: 1.13–2.96 μg RNA) was transcribed into cDNA. cDNA was purified using the QIAquick PCR purification kit (Qiagen). Nucleic acid concentrations were photometrically determined with an Implen NanoPhotometer (Implen, Munich, Germany).

### 4.2 Primer Design

Primers (Table S8) were designed with NCBI Primer‐Blast (Ye et al. 2012) within different potential exons of LOC409868 of the Amel_4.5 genome. Potential exons were identified as follows: The mRNA entry HP491739.1 (Elsik et al. 2014) was manually searched for the protein coding sequence (CDS) using the peptides originally identified in the mass spectrometric study on larval food (Zhang et al. 2014). The CDS (start to stop codon, 1827 bp) was blasted (blastn, NCBI database (Altschul et al. 1990)) against *A. mellifera* whole genome shotgun contigs which resulted in several hits, amongst others to *Apis mellifera* isolate Amel_TP_3 contig_39 (JBGNXS010000264.1) which was published within BioProject PRJNA1076921 aiming on the production of rapid and cost‐effective high‐quality insect genomes (Gilligan et al. 2024, bioRxiv). The 1827 bp long CDS spanned 39,418 bp on the contig with long introns between potential exon 1 and 2, exon 2 and 3, exon 3 and 4 as well as exon 9 and 10 (Figure 1A, Figure S1). Forward primers were designed in potential exon 1, 2, 3, 4 and 6 and revers primers in exon 2, 4, 9 and 10 (Figure S1, Table S8). Expected PCR product sizes based on HP491739.1 and JBGNXS010000264.1 using cDNA or DNA as template are given in Figure 1A and Table S8. Even though *matrix metalloproteinases* are duplicated genes, they only have about 50% sequence identity and none of the primers used for *mmp1* was predicted to bind in *mmp2* or *mmp3* (minimum 10 mismatches in *mmp2* or *mmp3*). All primer combinations produced only a single product during PCR with the expected size (except for those implementing the exon 9 and 10 boundary when using cDNA as template) and sequenced PCR products resulted in the expected *mmp1* sequence. Thus, the here developed primers can be regarded as specific for *mmp1*.

### 4.3 Polymerase Chain Reaction (PCR) and Sequencing

Reactions for the amplification from cDNA to evaluate gene expression were performed in a total of 25 μL. Thirty nanogram cDNA were mixed with 1 μL of each primer (10 μM), 12.5 μL GoTaq Colourless Mastermix (Promega) and ultrapure water. PCR was performed in a Mastercycler thermocycler (Eppendorf, Hamburg, Germany) and an initial denaturation step of 2 min at 95°C was followed by 30 amplification cycles (95°C, 15 s; 57°C, 30 s; 72°C, 90 s). Products were analysed on 1.5% agarose gels in TBE buffer (100 V, 2 h) using the 1 kb DNA ladder (New England Biolabs (NEB), Ipswich, MA, USA) and the GenLadder 100 bp Plus (Genaxxon bioscience GmbH, Ulm, Germany). Reactions for the amplification from DNA were performed in a total of 50 μL. One hundred nanogram DNA were mixed with 0.3 μL of each primer (10 μM), 25 μL GoTaq Long PCR Mastermix (Promega) and ultrapure water. An initial denaturation step of 2 min at 94°C was followed by 30 amplification cycles (94°C, 15 s; 57°C, 30 s; 65°C, 10–40 min). Products were analysed on 0.5% agarose gels in TAE buffer (50 V, 5 h) using the GeneRuler High Range DNA ladder (Thermo Fisher Scientific, Waltham, MA, USA) and the 1 kb DNA ladder (NEB). PCR product lengths were determined with GelAnalyzer 23.1.1 (available at www.gelanalyzer.com by Istvan Lazar Jr., PhD and Istvan Lazar Sr., PhD, CSc) and given as mean ± standard deviation from multiple PCRs. For sequencing, PCR products were purified using the QIAquick PCR purification kit (Qiagen) and sent to Eurofins Genomics (Sequencing lab, Cologne, Germany) (Data S1, https://doi.org/10.6084/m9.figshare.31960203).

### 4.4 Identification of Further *mmp* Genes

To identify genes coding for *mmps* in the buff‐tailed bumble bee *Bombus terrestris* and to identify further *mmp* genes in *A. mellifera* , the following proteins were blasted (tblastn, NCBI database (Altschul et al. 1990)) against the iyBomTerr1.2 (Crowley et al. 2023), the Amel_4.5 (Elsik et al. 2014) and the Amel_HAv3.1 (Wallberg et al. 2019) genome annotation: *A. mellifera* MMP1 isoforms 1 and 4 (identified in this study), *B. mori* MMP1 isoform 1 (NP_001116499.1), MMP2 (XP_004932298.1) and MMP3 (XP_004921585.2) (Jia et al. 2018), *D. melanogaster* MMP1 isoforms C (NP_726473.2), F (NP_001189000.1) and J (NP_001189004.1), MMP2 isoform B (NP_995788.1) (as these isoforms have been confirmed to be indeed expressed) (Page‐McCaw et al. 2003), *T. castaneum* MMP1 isoform 1 (NP_001157646.1), MMP2 (XP_969495.1) and MMP3 (XP_972146.1) (Knorr et al. 2009).

### 4.5 Analysis of Protein Sequences

The maximum likelihood tree was reconstructed from aligned MMP protein sequences (Table S2) ( *A. gambiae* , *A. mellifera* , *B. mori* , *B. terrestris* , *D. melanogaster* , *T. castaneum* , *H. sapiens* , (Page‐McCaw et al. 2003; Knorr et al. 2009; Goulielmaki et al. 2014; Jia et al. 2018; Laronha and Caldeira 2020) with human hemopexin (Takahashi et al. 1985) as outgroup) using the Whelan and Goldman (WAG) (+G, +I, +F) model (Whelan and Goldman 2001), including bootstrap resampling (500 replications) (Felsenstein 1985) to verify the topology of the inferred phylogenetic tree. Protein sequence alignment, model selection and tree reconstruction were performed in MEGA version 12 (Kumar et al. 2024). Signal peptides were predicted using SignalP6.0 (Teufel et al. 2022), Asparagine(N)‐linked glycosylations using NetNGlyc (Gupta and Brunak 2002) and protein domains and protein family membership using InterPro version 107.0 (Blum et al. 2025; Paysan‐Lafosse et al. 2025). Potential functions were inferred using the I‐TASSER server (Yang and Zhang 2015; Zheng et al. 2021) based on structural homologues identified within the protein data bank (PDB) (Berman et al. 2000).

### 4.6 Identification of Transcripts in the Sequence Read Archive (SRA) Database

To further identify transcripts of *mmp1* (isoform 2), a nucleotide BLAST (Altschul et al. 1990) with default algorithm parameters but a maximum of 5000 target sequences was performed against worker whole body larval to adult, hemocyte, mandibular gland (MDG), hypopharyngeal gland (HPG) and worker and queen larval ovary RNA‐seq entries within the SRA database of NCBI (Sayers et al. 2025). These entries were generated within the BioProjects PRJNA1247573 (larval hemocytes: SRX28274185; adult hemocytes: SRX28274184) (Braeckman et al. 2025), PRJNA243651 (nurse bee MDGs: SRX519338, SRX519339, SRX519340; nurse bee HPGs: SRX518718, SRX518719, SRX518720; pollen forager MDGs: SRX518061, SRX519335, SRX519336; pollen forager HPGs: SRX518059, SRX518716, SRX518717) (Jasper et al. 2015), PRJDB14753 (larva to adult: DRA016719) (Yokoi et al. 2025) and PRJNA636804 (queen larvae ovaries: SRX8484801; worker larvae ovaries: SRX8484800) (Lago et al. 2022). For identifiers of aligning sequences please see (Data S2, https://doi.org/10.6084/m9.figshare.31960203).

### 4.7 BUSCO Gene Content

BUSCO (benchmarking universal single‐copy orthologues) version 6.0.0 (Simão et al. 2015; Tegenfeldt et al. 2025) was used to compare BUSCO genes between Amel_4.5 and Amel_HAv3.1 by assessing complete, duplicated, fragmented or missing single‐copy orthologues as inferred from OrthoDB version 12 (Tegenfeldt et al. 2025) in both assemblies (https://ftp.ncbi.nlm.nih.gov/genomes/all/GCF/000/002/195/GCF_000002195.4_Amel_4.5/GCF_000002195.4_Amel_4.5_genomic.fna.gz & https://ftp.ncbi.nlm.nih.gov/genomes/all/GCF/003/254/395/GCF_003254395.2_Amel_HAv3.1/GCF_003254395.2_Amel_HAv3.1_genomic.fna.gz). The Metazoan (*n* = 672 BUSCO genes) and the Hymenoptera (*n* = 5920 BUSCO genes) gene sets were used to identify all BUSCO genes (Tables S3–S7) and to specifically determine those (15) that are present in Amel_4.5 but missing in Amel_HAv3.1 (Table S3). These 15 genes were searched in the Amel_4.5 assembly using the NCBI Genome Data Viewer (Sayers et al. 2025), the corresponding loci as well as their flanking loci determined and the protein sequences extracted (Table S3). Subsequently all thereby identified loci and protein sequences were tried to be located in the Amel_HAv3.1 assembly by either searching the loci in the NCBI Genome Data Viewer (Sayers et al. 2025) or by blasting the protein sequences (tblastn, NCBI database (Altschul et al. 1990)) against the reference genome (Table S3).

## Author Contributions

**Jessica Braunisch:** formal analysis (equal), investigation (equal), writing – review and editing (equal). **Ken Werner:** data curation (equal), methodology (equal), software (equal), writing – review and editing (equal). **Ulrich R. Ernst:** resources (equal), writing – review and editing (equal). **Christian W. W. Pirk:** data curation (supporting), formal analysis (supporting), writing – review and editing (equal). **Anja Buttstedt:** conceptualization (lead), data curation (equal), formal analysis (equal), funding acquisition (lead), investigation (equal), methodology (equal), project administration (lead), resources (equal), software (equal), supervision (lead), validation (lead), visualization (lead), writing – original draft (lead), writing – review and editing (equal).

## Funding

This work was supported by Deutsche Forschungsgemeinschaft, 534955761.

## Conflicts of Interest

The authors declare no conflicts of interest.

## Supporting information


**Figure S1:** Alignment of Sanger sequencing of DNA PCR products with genomic sequence (JBGNXS010000264.1, bases 495,000 to 534,700) covering Amel_4.5_LOC409868 (Elsik et al. 2014) published within BioProject PRJNA1026921 (Gilligan et al. 2024, bioRxiv). Blue: potential exons deduced from the mRNA sequence HP491739.1 (Elsik et al. 2014); yellow: potential introns; red: potential start and stop codon. Underlined, bold, sequences in italics are primer sequences.
**Figure S2:** Agarose gels (1.5%, TBE buffer, 100 V, 2 h) showing PCR products of mmp_LOC409868 amplification from cDNA (RT‐PCR) of four different forager bees. H, head; T, thorax; A, abdomen. As expression in thoraces was barely detectable for the first two primer pairs (3–9 and 3–10), thorax cDNA was omitted as template in all other PCRs.
**Figure S3:** Alignment of Sanger sequencing of cDNA PCR products including different isoforms with genomic sequence (JBGNXS010000264.1) covering Amel_4.5_LOC409868 (Elsik et al. 2014) published within BioProject PRJNA1026921 (Gilligan et al. 2024, bioRxiv). Blue: exons deduced from sequenced cDNA PCR products; yellow: introns deduced from sequenced cDNA PCR products, long introns have been shortened using dashes; red: potential start and stop codon. Underlined, bold, sequences in italics are primer sequences.
**Figure S4:** Numbers of sequences producing significant alignments against mmp1‐isoform 2. The RNAseq data were made available online as part BioProject PRJDB14753 (Yokoi et al. 2025) in which an expression dataset for *A. mellifera* worker larva to adult development was published. The dataset comprises a single individual for days 6–8 and 23–58, two individuals for day 13, three individuals for days 12, four individuals for day 10, 14 and 15 and five individuals for days 9, 11 and 16–22. The graph shows means ± standard deviation for days with replicates.

**Table S1:** Blast hits of the here sequenced genomic mmp1 sequence (PX921700) to the long‐read raw data of Amel_HAv3.1 uploaded to the sequence read archive (SRA) database (SRX6817383, Pacific Biosciences SMRT sequencing).
**Table S2:** MMP protein sequences used to construct the maximum likelihood tree.
**Table S3:** Metazoan and Hymenopteran BUSCO genes that are reported as ‘present’ in Amel_4.5 and ‘missing’ in Amel_HAv3.1.
**Table S4:** Full list of Metazoan BUSCO genes identified in Amel_4.5.
**Table S5:** Full list of Hymenopteran BUSCO genes identified in Amel_4.5.
**Table S6:** Full list of Metazoan BUSCO genes identified in Amel_HAv3.1.
**Table S7:** Full list of Hymenopteran BUSCO genes identified in Amel_HAv3.1.
**Table S8:** Primers designed for this study. PCR products in bold and highlighted in green were amplified successfully, products highlighted in red could not be amplified, products in italics and highlighted in grey were not tried to be amplified as the region was covered by other products.

## Data Availability

All data are incorporated into the article and its online Supporting Information. The *mmp1* gene sequence including expressed isoforms was uploaded to GenBank (PX921700). Data S1 and S2 were uploaded to figshare (https://doi.org/10.6084/m9.figshare.31960203).

## References

[ece374600-bib-0001] Adams, M. D. , S. E. Celniker , R. A. Holt , et al. 2000. “The Genome Sequence of *Drosophila melanogaster* .” Science 287, no. 5461: 2185–2195. 10.1126/science.287.5461.2185. 10731132

[ece374600-bib-0002] Altschul, S. F. , W. Gish , W. Miller , E. W. Myers , and D. J. Lipman . 1990. “Basic Local Alignment Search Tool.” Journal of Molecular Biology 215, no. 3: 403–410. 10.1016/S0022-2836(05)80360-2. 2231712

[ece374600-bib-0003] Berman, H. M. , J. Westbrook , Z. Feng , et al. 2000. “The Protein Data Bank.” Nucleic Acids Research 28, no. 1: 235–242. 10.1093/nar/28.1.235. PMC102472 10592235

[ece374600-bib-0004] Blum, M. , A. Andreeva , L. C. Florentino , et al. 2025. “InterPro: The Protein Sequence Classification Resource in 2025.” Nucleic Acids Research 53, no. D1: D444–D456. 10.1093/nar/gkae1082. PMC11701551 39565202

[ece374600-bib-0005] Boch, R. , and D. A. Shearer . 1963. “Production of Geraniol by Honey Bees of Various Ages.” Journal of Insect Physiology 9, no. 4: 431–434. 10.1016/0022-1910(63)90052-0.

[ece374600-bib-0006] Boch, R. , and D. A. Shearer . 1966. “2‐Heptanone and 10‐Hydroxy‐Transdec‐2‐Enoic Acid in the Mandibular Glands of Worker Honey Bees of Different Ages.” Zeitschrift für Vergleichende Physiologie 54: 1–11. 10.1007/BF00298205.

[ece374600-bib-0007] Bond, N. D. , A. Nelliot , M. K. Bernardo , et al. 2011. “ßFTZ‐F1 and Matrix Metalloproteinase 2 Are Required for Fat‐Body Remodeling in *Drosophila* .” Developmental Biology 360, no. 2: 286–296. 10.1016/j.ydbio.2011.09.015. 21978772

[ece374600-bib-0008] Braeckman, M. , L. De Smet , B. Devreese , and D. C. de Graaf . 2025. “Exploration of the Hemocyte Surfaceome of *Apis mellifera* by a Proteomic and Transcriptomic Approach.” Insect Biochemistry and Molecular Biology 184: 104398. 10.1016/j.ibmb.2025.104398. 40915621

[ece374600-bib-0009] Brutscher, L. M. , K. F. Daughenbaugh , and M. L. Flenniken . 2017. “Virus and dsRNA‐Triggered Transcriptional Responses Reveal Key Components of Honey Bee Antiviral Defense.” Scientific Reports 7, no. 1: 6448. 10.1038/s41598-017-06623-z. PMC5526946 28743868

[ece374600-bib-0010] Buttstedt, A. , C. W. W. Pirk , and A. A. Yusuf . 2023. “Mandibular Glands Secrete 24‐Methylenecholesterol Into Honey Bee ( *Apis mellifera* ) Food Jelly.” Insect Biochemistry and Molecular Biology 161: 104011. 10.1016/j.ibmb.2023.104011. 37716535

[ece374600-bib-0011] Capella, I. C. , and K. Hartfelder . 1998. “Juvenile Hormone Effect on DNA Synthesis and Apoptosis in Caste‐Specific Differentiation of the Larval Honey Bee ( *Apis mellifera* L.) Ovary.” Journal of Insect Physiology 44, no. 5–6: 385–391. 10.1016/s0022-1910(98)00027-4. 12770156

[ece374600-bib-0012] Cassier, P. , and Y. Lensky . 1997. “The Exocrine Glands of the Honey Bees.” In Bee Products, edited by A. Mizrahi and Y. Lensky . Springer. 10.1007/978-1-4757-9371-0_18.

[ece374600-bib-0013] Casteels‐Josson, K. , W. Zhang , T. Capaci , P. Casteels , and P. Tempst . 1994. “Acute Transcriptional Response of the Honeybee Peptide‐Antibiotics Gene Repertoire and Required Post‐Translational Conversion of the Precursor Structures.” Journal of Biological Chemistry 269, no. 46: 28569–28575. 10.1016/S0021-9258(19)61943-5. 7961803

[ece374600-bib-0014] Chapman, A. , A. McAfee , K. L. C. Wrightson , et al. 2025. “Honey Bee Egg Composition Changes Seasonally and After Acute Maternal Virus Infection.” Scientific Reports 15, no. 1: 10418. 10.1038/s41598-025-94670-2. PMC11947112 40140730

[ece374600-bib-0015] Creevey, C. J. , J. Muller , T. Doerks , J. D. Thompson , D. Arendt , and P. Bork . 2011. “Identifying Single Copy Orthologs in Metazoa.” PLoS Computational Biology 7, no. 12: e1002269. 10.1371/journal.pcbi.1002269. PMC3228760 22144877

[ece374600-bib-0016] Crowley, L. M. , O. Sivell , D. Sivell , et al. 2023. “The Genome Sequence of the Buff‐Tailed Bumblebee, *Bombus terrestris* (Linnaeus, 1758).” Wellcome Open Research 8: 161. 10.12688/wellcomeopenres.19248.1. PMC10818103 38283327

[ece374600-bib-0017] Das, S. , M. Mandal , T. Chakraborti , A. Mandal , and S. Chakraborti . 2003. “Structure and Evolutionary Aspects of Matrix Metalloproteinases: A Brief Overview.” Molecular and Cellular Biochemistry 253, no. 1–2: 31–40. 10.1023/a:1026093016148. 14619953

[ece374600-bib-0018] Dobritzsch, D. , D. Aumer , M. Fuszard , S. Erler , and A. Buttstedt . 2019. “The Rise and Fall of Major Royal Jelly Proteins During a Honeybee ( *Apis mellifera* ) Workers' Life.” Ecology and Evolution 9, no. 15: 8771–8782. 10.1002/ece3.5429. PMC6686338 31410279

[ece374600-bib-0019] Doublet, V. , R. J. Paxton , C. M. McDonnell , et al. 2016. “Brain Transcriptomes of Honey Bees ( *Apis mellifera* ) Experimentally Infected by Two Pathogens: Black Queen Cell Virus and *Nosema ceranae* .” Genomics Data 10: 79–82. 10.1016/j.gdata.2016.09.010. PMC5054260 27747157

[ece374600-bib-0020] Doublet, V. , Y. Poeschl , A. Gogol‐Döring , et al. 2017. “Unity in Defence: Honeybee Workers Exhibit Conserved Molecular Responses to Diverse Pathogens.” BMC Genomics 18, no. 1: 207. 10.1186/s12864-017-3597-6. PMC5333379 28249569

[ece374600-bib-0021] Elsik, C. G. , K. C. Worley , A. K. Bennett , et al. 2014. “Finding the Missing Honey Bee Genes: Lessons Learned From a Genome Upgrade.” BMC Genomics 15: 86. 10.1186/1471-2164-15-86. PMC4028053 24479613

[ece374600-bib-0022] Felsenstein, J. 1985. “Confidence Limits on Phylogenies: An Approach Using the Bootstrap.” Evolution 39, no. 4: 783–791. 10.1111/j.1558-5646.1985.tb00420.x. 28561359

[ece374600-bib-0023] Fujiwara, S. , J. Imai , M. Fujiwara , T. Yaeshima , T. Kawashima , and K. Kobayashi . 1990. “A Potent Antibacterial Protein in Royal Jelly. Purification and Determination of the Primary Structure of Royalisin.” Journal of Biological Chemistry 265, no. 19: 11333–11337. 10.1016/S0021-9258(19)38596-5. 2358464

[ece374600-bib-0024] Galbraith, D. A. , X. Yang , E. L. Niño , S. Yi , and C. Grozinger . 2015. “Parallel Epigenomic and Transcriptomic Responses to Viral Infection in Honey Bees ( *Apis mellifera* ).” PLoS Pathogens 11, no. 3: e1004713. 10.1371/journal.ppat.1004713. PMC4374888 25811620

[ece374600-bib-0025] Gersh, I. , and H. R. Catchpole . 1949. “The Organization of Ground Substance and Basement Membrane and Its Significance in Tissue Injury Disease and Growth.” American Journal of Anatomy 85, no. 3: 457–521. 10.1002/aja.1000850304. 15400306

[ece374600-bib-0026] Gilligan, J. , S. N. Inwood , T. W. R. Harrop , et al. 2024. “Rapid and Cost‐Effective Production of High‐Quality Insect Genome Sequences.” *bioRxiv*: The Preprint Server for Biology. 10.1101/2024.10.06.616902.

[ece374600-bib-0027] Glasheen, B. M. , R. M. Robbins , C. Piette , G. J. Beitel , and A. Page‐McCaw . 2010. “A Matrix Metalloproteinase Mediates Airway Remodeling in *Drosophila* .” Developmental Biology 344, no. 2: 772–783. 10.1016/j.ydbio.2010.05.504. PMC2914218 20513443

[ece374600-bib-0028] Goulielmaki, E. , I. Sidén‐Kiamos , and T. G. Loukeris . 2014. “Functional Characterization of *Anopheles Matrix Metalloprotease 1 r*eveals Its Agonistic Role During Sporogonic Development of Malaria Parasites.” Infection and Immunity 82, no. 11: 4865–4877. 10.1128/IAI.02080-14. PMC4249346 25183733

[ece374600-bib-0029] Gupta, R. , and S. Brunak . 2002. “Prediction of Glycosylation Across the Human Proteome and the Correlation to Protein Function.” Pacific Symposium on Biocomputing 2002, 310–322. 11928486

[ece374600-bib-0030] Hepburn, H. R. , R. T. F. Bernard , B. C. Davidson , et al. 1991. “Synthesis and Secretion of Beeswax in Honeybees.” Apidologie 22, no. 1: 21–36. 10.1051/apido:19910104.

[ece374600-bib-0031] Holt, R. A. , G. M. Subramanian , A. Halpern , et al. 2002. “The Genome Sequence of the Malaria Mosquito *Anopheles gambiae* .” Science 298, no. 5591: 129–149. 10.1126/science.1076181. 12364791

[ece374600-bib-0032] Honeybee Genome Sequencing Consortium . 2006. “Insights Into Social Insects From the Genome of the Honeybee *Apis mellifera* .” Nature 443, no. 7114: 931–949. 10.1038/nature05260. PMC2048586 17073008

[ece374600-bib-0033] Hotaling, S. , J. S. Sproul , J. Heckenhauer , et al. 2021. “Long Reads Are Revolutionizing 20 Years of Insect Genome Sequencing.” Genome Biology and Evolution 13, no. 8: evab138. 10.1093/gbe/evab138. PMC8358217 34152413

[ece374600-bib-0034] International Human Genome Sequencing Consortium . 2001. “Initial Sequencing and Analysis of the Human Genome.” Nature 409, no. 6822: 860–921. 10.1038/35057062. 11237011

[ece374600-bib-0035] Jasper, W. C. , T. A. Linksvayer , J. Atallah , D. Friedman , J. C. Chiu , and B. R. Johnson . 2015. “Large‐Scale Coding Sequence Change Underlies the Evolution of Postdevelopmental Novelty in Honey Bees.” Molecular Biology and Evolution 32, no. 2: 334–346. 10.1093/molbev/msu292. 25351750

[ece374600-bib-0036] Jia, Q. , X. Chen , L. Wu , Z. Ruan , K. Li , and S. Li . 2018. “Matrix Metalloproteinases Promote Fat Body Cell Dissociation and Ovary Development in *Bombyx mori* .” Journal of Insect Physiology 111: 8–15. 10.1016/j.jinsphys.2018.10.002. 30300619

[ece374600-bib-0037] Kaskinova, M. , B. Yunusbayev , R. Altinbaev , et al. 2021. “Improved *Apis mellifera* Reference Genome Based on the Alternative Long‐Read‐Based Assemblies.” G3: Genes, Genomes, Genetics 11, no. 9: jkab223. 10.1093/g3journal/jkab223. PMC8661400 34544128

[ece374600-bib-0038] Knorr, E. , H. Schmidtberg , A. Vilcinskas , and B. Altincicek . 2009. “MMPs Regulate Both Development and Immunity in the *Tribolium* Model Insect.” PLoS One 4, no. 3: e4751. 10.1371/journal.pone.0004751. PMC2649432 19270735

[ece374600-bib-0039] Kriventseva, E. V. , D. Kuznetsov , F. Tegenfeldt , et al. 2019. “OrthoDB v10: Sampling the Diversity of Animal, Plant, Fungal, Protist, Bacterial and Viral Genomes for Evolutionary and Functional Annotations of Orthologs.” Nucleic Acids Research 47, no. D1: D807–D811. 10.1093/nar/gky1053. PMC6323947 30395283

[ece374600-bib-0040] Kumar, S. , G. Stecher , M. Suleski , M. Sanderford , S. Sharma , and K. Tamura . 2024. “MEGA 12: Molecular Evolutionary Genetics Analysis Version 12 for Adaptive and Green Computing.” Molecular Biology and Evolution 41, no. 12: msae263. 10.1093/molbev/msae263. PMC11683415 39708372

[ece374600-bib-0041] LaFever, K. , X. Wang , P. Page‐McCaw , G. Bhave , and P. Page‐McCaw . 2017. “Both *Drosophila* Matrix Metalloproteinases Have Released and Membrane‐Tethered Forms but Have Different Substrates.” Scientific Reports 7: 44560. 10.1038/srep44560. PMC5353688 28300207

[ece374600-bib-0042] Lago, D. C. , M. Hasselmann , and K. Hartfelder . 2022. “Sex‐ and Caste‐Specific Transcriptomes of Larval Honey Bee ( *Apis mellifera* L.) Gonads: DMRT A2 and Hsp83 Are Differentially Expressed and Regulated by Juvenile Hormone.” Insect Molecular Biology 31, no. 5: 593–608. 10.1111/imb.12782. 35524973

[ece374600-bib-0043] Langlands, Z. , E. E. Du Rand , A. A. Yusuf , and C. W. W. Pirk . 2022. “Functional Response of the Hypopharyngeal Glands to a Social Parasitism Challenge in Southern African Honey Bee Subspecies.” Parasitology Research 121: 267–274. 10.1007/s00436-021-07391-6. 34988669

[ece374600-bib-0044] Laronha, H. , and J. Caldeira . 2020. “Structure and Function of Human Matrix Metalloproteinases.” Cells 9, no. 5: 1076. 10.3390/cells9051076. PMC7290392 32357580

[ece374600-bib-0045] Li, F. , X. Wang , and X. Zhou . 2025. “The Genomics Revolution Drives a New Era in Entomology.” Annual Review of Entomology 70, no. 1: 379–400. 10.1146/annurev-ento-013024-013420. 39874145

[ece374600-bib-0046] Liu, T. H. , T. H. Liu , Y. Wei , et al. 2022. “The Dual Roles of Three MMPs and TIMP in Innate Immunity and Metamorphosis in the Silkworm, *Bombyx mori* .” FEBS Journal 289, no. 10: 2828–2846. 10.1111/febs.16313. 34862848

[ece374600-bib-0047] Lu, D. , C. Liu , W. Ji , et al. 2024. “Nanopore Ultra‐Long Sequencing and Adaptive Sampling Spur Plant Complete Telomere‐to‐Telomere Genome Assembly.” Molecular Plant 17, no. 11: 1773–1786. 10.1016/j.molp.2024.10.008. 39420560

[ece374600-bib-0048] McAfee, A. , J. Milone , A. Chapman , L. J. Foster , J. S. Pettis , and D. R. Tarpy . 2020. “Candidate Stress Biomarkers for Queen Failure Diagnostics.” BMC Genomics 21, no. 1: 571. 10.1186/s12864-020-06992-2. PMC7441638 32819278

[ece374600-bib-0049] Mei, Y. , D. Jing , S. Tang , et al. 2022. “InsectBase 2.0: A Comprehensive Gene Resource for Insects.” Nucleic Acids Research 50, no. D1: D1040–D1045. 10.1093/nar/gkab1090. PMC8728184 34792158

[ece374600-bib-0050] Mitten, E. K. , D. Jing , and Y. Suzuki . 2012. “Matrix Metalloproteinases (MMPs) Are Required for Wound Closure and Healing During Larval Leg Regeneration in the Flour Beetle, *Tribolium castaneum* .” Insect Biochemistry and Molecular Biology 42, no. 11: 854–864. 10.1016/j.ibmb.2012.08.001. 22940602

[ece374600-bib-0051] Mumoki, F. N. , C. W. W. Pirk , R. M. Crewe , and A. A. Yusuf . 2025. “Transcriptomic Analysis of the Mandibular Gland Genes Associated With Reproductive Dominance in *Apis mellifera capensis* Esch. Parasitic Workers.” Physiological Entomology 50, no. 3: 233–244. 10.1111/phen.12484.

[ece374600-bib-0052] Okosun, O. O. , A. A. Yusuf , R. M. Crewe , and C. W. Pirk . 2015. “Effects of Age and Reproductive Status on Tergal Gland Secretions in Queenless Honey Bee Workers, *Apis mellifera scutellata* and *A. m. capensis* .” Journal of Chemical Ecology 41, no. 10: 896–903. 10.1007/s10886-015-0630-6. 26384295

[ece374600-bib-0053] Owen, M. D. , and A. R. Bridges . 1976. “Aging in the Venom Glands of Queen and Worker Honey Bees ( *Apis mellifera* L.): Some Morphological and Chemical Observations.” Toxicon 14, no. 1: 1–5. 10.1016/0041-0101(76)90113-6. 1258064

[ece374600-bib-0054] Page‐McCaw, A. , A. J. Ewald , and Z. Werb . 2007. “Matrix Metalloproteinases and the Regulation of Tissue Remodelling.” Nature Reviews. Molecular Cell Biology 8, no. 3: 221–233. 10.1038/nrm2125. PMC2760082 17318226

[ece374600-bib-0055] Page‐McCaw, A. , J. Serano , J. M. Santé , and G. M. Rubin . 2003. “ *Drosophila* Matrix Metalloproteinases Are Required for Tissue Remodeling, but Not Embryonic Development.” Developmental Cell 4, no. 1: 95–106. 10.1016/s1534-5807(02)00400-8. 12530966

[ece374600-bib-0056] Paysan‐Lafosse, T. , A. Andreeva , M. Blum , et al. 2025. “The Pfam Protein Families Database: Embracing AI/ML.” Nucleic Acids Research 53, no. D1: D523–D534. 10.1093/nar/gkae997. PMC11701544 39540428

[ece374600-bib-0057] Rawlings, N. D. , and A. J. Barrett . 1995. “Evolutionary Families of Metallopeptidases.” Methods in Enzymology 248: 183–228. 10.1016/0076-6879(95)48015-3. 7674922

[ece374600-bib-0058] Saitou, N. , and M. Nei . 1987. “The Neighbor‐Joining Method: A New Method for Reconstructing Phylogenetic Trees.” Molecular Biology and Evolution 4, no. 4: 406–425. 10.1093/oxfordjournals.molbev.a040454. 3447015

[ece374600-bib-0059] Sayers, E. W. , J. Beck , E. E. Bolton , et al. 2025. “Database Resources of the National Center for Biotechnology Information in 2025.” Nucleic Acids Research 53, no. D1: D20–D29. 10.1093/nar/gkae979. PMC11701734 39526373

[ece374600-bib-0060] Seeley, T. D. 1982. “Adaptive Significance of the Age Polyethism Schedule in Honey Bee Colonies.” Behavioral Ecology and Sociobiology 11: 287–293. 10.1007/BF00299306.

[ece374600-bib-0061] Shafee, T. M. , F. T. Lay , T. K. Phan , M. A. Anderson , and M. D. Hulett . 2017. “Convergent Evolution of Defensin Sequence, Structure and Function.” Cellular and Molecular Life Sciences 74, no. 4: 663–682. 10.1007/s00018-016-2344-5. PMC11107677 27557668

[ece374600-bib-0062] Simão, F. A. , R. M. Waterhouse , P. Ioannidis , E. V. Kriventseva , and E. M. Zdobnov . 2015. “BUSCO: Assessing Genome Assembly and Annotation Completeness With Single‐Copy Orthologs.” Bioinformatics 31, no. 19: 3210–3212. 10.1093/bioinformatics/btv351. 26059717

[ece374600-bib-0063] Soudek, Š. 1927. “Hltanové žlázy včely medonosné.” Sborník Vysoké Školy Zemědělské v Brně, ČSR, Fakulta Hospodářská C10: 1–66.

[ece374600-bib-0064] Stevens, L. J. , and A. Page‐McCaw . 2012. “A Secreted MMP Is Required for Reepithelialization During Wound Healing.” Molecular Biology of the Cell 23, no. 6: 1068–1079. 10.1091/mbc.E11-09-0745. PMC3302734 22262460

[ece374600-bib-0065] Takahashi, N. , Y. Takahashi , and F. W. Putnam . 1985. “Complete Amino Acid Sequence of Human Hemopexin, the Heme‐Binding Protein of Serum.” Proceedings of the National academy of Sciences of the United States of America 82, no. 1: 73–77. 10.1073/pnas.82.1.73. PMC396973 3855550

[ece374600-bib-0066] Tegenfeldt, F. , D. Kuznetsov , M. Manni , M. Berkeley , E. M. Zdobnov , and E. V. Kriventseva . 2025. “OrthoDB and BUSCO Update: Annotation of Orthologs With Wider Sampling of Genomes.” Nucleic Acids Research 53, no. D1: D516–D522. 10.1093/nar/gkae987. PMC11701741 39535043

[ece374600-bib-0067] Teufel, F. , J. J. Almagro Armenteros , A. R. Johansen , et al. 2022. “SignalP 6.0 Predicts All Five Types of Signal Peptides Using Protein Language Models.” Nature Biotechnology 40, no. 7: 1023–1025. 10.1038/s41587-021-01156-3. PMC9287161 34980915

[ece374600-bib-0068] Wallberg, A. , I. Bunikis , O. V. Pettersson , et al. 2019. “A Hybrid De Novo Genome Assembly of the Honeybee, *Apis mellifera* , With Chromosome‐Length Scaffolds.” BMC Genomics 20, no. 1: 275. 10.1186/s12864-019-5642-0. PMC6454739 30961563

[ece374600-bib-0069] Wang, Z. , H. Chen , S. Jin , et al. 2026. “InsectBase 3.0: A Comprehensive Multi‐Omics Resource for Insects.” Nucleic Acids Research 54, no. D1: D1143–D1151. 10.1093/nar/gkaf1248. PMC12807663 41263103

[ece374600-bib-0070] Waterhouse, R. M. , E. M. Zdobnov , and E. V. Kriventseva . 2011. “Correlating Traits of Gene Retention, Sequence Divergence, Duplicability and Essentiality in Vertebrates, Arthropods, and Fungi.” Genome Biology and Evolution 3: 75–86. 10.1093/gbe/evq083. PMC3030422 21148284

[ece374600-bib-0071] Wei, Y. , X. L. Zhou , T. H. Liu , et al. 2021. “A Matrix Metalloproteinase Mediates Tracheal Development in *Bombyx mori* .” International Journal of Molecular Sciences 22, no. 11: 5618. 10.3390/ijms22115618. PMC8198827 34070691

[ece374600-bib-0072] Wen, D. , Z. Chen , Z. Zhang , and Q. Jia . 2020. “The Expression, Purification, and Substrate Analysis of Matrix Metalloproteinases in *Drosophila melanogaster* .” Protein Expression and Purification 171: 105629. 10.1016/j.pep.2020.105629. 32201229

[ece374600-bib-0073] Whelan, S. , and N. Goldman . 2001. “A General Empirical Model of Protein Evolution Derived From Multiple Protein Families Using a Maximum‐Likelihood Approach.” Molecular Biology and Evolution 18, no. 5: 691–699. 10.1093/oxfordjournals.molbev.a003851. 11319253

[ece374600-bib-0074] Wilson, C. L. , A. J. Ouellette , D. P. Satchell , et al. 1999. “Regulation of Intestinal Alpha‐Defensin Activation by the Metalloproteinase Matrilysin in Innate Host Defense.” Science 286, no. 5437: 113–117. 10.1126/science.286.5437.113. 10506557

[ece374600-bib-0075] Xu, J. , G. Grant , L. R. Sabin , et al. 2012. “Transcriptional Pausing Controls a Rapid Antiviral Innate Immune Response in *Drosophila* .” Cell Host & Microbe 12, no. 4: 531–543. 10.1016/j.chom.2012.08.011. PMC3479682 23084920

[ece374600-bib-0076] Yang, J. , and Y. Zhang . 2015. “I‐TASSER Server: New Development for Protein Structure and Function Predictions.” Nucleic Acids Research 43, no. W1: W174–W181. 10.1093/nar/gkv342. PMC4489253 25883148

[ece374600-bib-0077] Ye, J. , G. Coulouris , I. Zaretskaya , I. Cutcutache , S. Rozen , and T. L. Madden . 2012. “Primer‐BLAST: A Tool to Design Target‐Specific Primers for Polymerase Chain Reaction.” BMC Bioinformatics 13: 134. 10.1186/1471-2105-13-134. PMC3412702 22708584

[ece374600-bib-0078] Yin, C. , G. Shen , D. Guo , et al. 2016. “InsectBase: A Resource for Insect Genomes and Transcriptomes.” Nucleic Acids Research 44, no. D1: D801–D807. 10.1093/nar/gkv1204. PMC4702856 26578584

[ece374600-bib-0079] Yokoi, K. , M. Hatakeyama , S. Kuwazaki , et al. 2025. “Comprehensive Expression Data for Two Honey Bee Species, *Apis mellifera* and *Apis cerana japonica* .” Scientific Data 12, no. 1: 926. 10.1038/s41597-025-05279-z. PMC12130247 40456786

[ece374600-bib-0080] Yu, H. , H. Takeuchi , M. Takeuchi , et al. 2016. “Structural Analysis of Notch‐Regulating Rumi Reveals Basis for Pathogenic Mutations.” Nature Chemical Biology 12, no. 9: 735–740. 10.1038/nchembio.2135. PMC4990500 27428513

[ece374600-bib-0081] Yusuf, A. A. , C. W. W. Pirk , and A. Buttstedt . 2024. “Expression of Honey Bee ( *Apis mellifera* ) Sterol Homeostasis Genes in Food Jelly Producing Glands of Workers.” Journal of Experimental Zoology Part A: Ecological and Integrative Physiology 341, no. 5: 627–641. 10.1002/jez.2813. 38567629

[ece374600-bib-0082] Zhang, L. , B. Han , R. Li , et al. 2014. “Comprehensive Identification of Novel Proteins and N‐Glycosylation Sites in Royal Jelly.” BMC Genomics 15: 135. 10.1186/1471-2164-15-135. PMC3942810 24529077

[ece374600-bib-0083] Zhang, W. , L. Wang , Y. Zhao , et al. 2022. “Single‐Cell Transcriptomic Analysis of Honeybee Brains Identifies Vitellogenin as Caste Differentiation‐Related Factor.” iScience 25, no. 7: 104643. 10.1016/j.isci.2022.104643. PMC9254125 35800778

[ece374600-bib-0084] Zheng, W. , C. Zhang , Y. Li , R. Pearce , E. W. Bell , and Y. Zhang . 2021. “Folding Non‐Homologous Proteins by Coupling Deep‐Learning Contact Maps With I‐TASSER Assembly Simulations.” Cell Reports Methods 1, no. 3: 100014. 10.1016/j.crmeth.2021.100014. PMC8336924 34355210

