# Supplementary material for: Lost and Found: Western Honey Bee (Apis mellifera) Matrix Metalloproteinase 1
Source: Ecol Evol. 2026 Oct 27;16(11):e74600. doi: 10.1002/ece3.74600 (PMC13713223; doi:10.1002/ece3.74600)
Supplement: Supplementary file 1 [file ECE3-16-e74600-s002.pdf]

Supplementary for

Lost and found: Western honey bee (*Apis mellifera*) *matrix metalloprotease 1*

Jessica Braunisch, Ken Werner, Ulrich R. Ernst, Christian W.W. Pirk & Anja Buttstedt

Supplementary Results **1**

BUSCO analysis 1

Supplementary Tables **3**

Tables S1-S7 (Excel file) 3

Table S8 4

Supplementary Figures **4**

Figure S1 4

Figure S2 19

Figure S3 20

Figure S4 24

Supplementary results

BUSCO analysis (see also Table S3)

In the vicinity of LOC409868 (*mmp1*), two more loci encoding BUSCO genes were affected by the deletion on chromosome 10 in Amel_HAv3.1. LOC100577328 (NC_007079.3, location: 4,082,953 - 4,087,128) encodes in Amel_4.5 an O-glucosyltransferase which is a Hymenopteran (140244at7399) and Metazoan (4283313at33208) BUSCO gene and which glycosylates in *D. melanogaster* epidermal growth factor repeats in several proteins (Yu et al., 2016). LOC409869 (NC_007079.3, location: 4,152,697 - 4,286,249) encodes in Amel_4.5 kinesin-like protein KIF13, a Hymenopteran (140642at7399) BUSCO gene. Interestingly, LOC409869 is still present in Amel_HAv3.1 (NC_037647.1, location: 4,211,423 - 4,302,920) but encodes a completely different, much shorter protein (Amel_HAv3.1: 2 isoforms, 59 & 64 amino acids, respectively; Amel_4.5: 9 isoforms, 1899 - 1935 aa) with only 20.3 and 23.4% sequence identity. It is still called ‘kinesin 3A’ even though no kinesin-domain (InterPro) could be detected. Two more loci that were located on chromosome 10 and are now missing are: LOC408305 (uridine-cytidine kinase-like 1) (location: 2,693,786 - 2,696,621) and LOC410247 (uncharacterized protein) (location: 2,696,532 - 2,700,914) located adjacent to each other flanked by loci LOC410241 (Rab10) and LOC408306 (sodium/hydrogen exchanger 3). The flanking loci were also present in Amel_HaV3.1, encoding the very same proteins, but the loci in between were missing. Whereas LOC408305 cannot be found at all, the full exon 1 of

LOC410247 in Amel_4.5 can still be found on chromosome 10 between bases 2,784,454 to 2,784,738 in Amel_HAv3.1 but the rest of the gene is missing. This time no alignment gap is visible in Amel_HAv3.1; the sequence is just not there.

On chromosome 13, the following genes are missing: LOC408404 (mitochondrial ribosomal protein L16) (NC_007082.3, location 1,710,173 - 1,711,390) located next to a 170,000 bp gap in Amel_4.5 and between the loci LOC102654165 (transmembrane protein 132E) as well as LOC100576527 (cycAMP-dependent transcription factor ATF-2). The flanking loci are present and code for the same proteins, but in between is a different locus LOC113218575 (neprylisin-1-like) and a 200 bp alignment gap. LOC408404 cannot be found anywhere. Neprylisin-1-like exists in Amel_4.5 as well but is located at a different position on the same chromosome next to another alignment gap. Furthermore, LOC410425 (NC_007082.3, location 2,208,932 - 2,219,588) and LOC408408 (NC_007082.3, location 2,219,765 - 2,255,728) are adjacent to each other on chromosome 13 in Amel_4.5. LOC410425 is completely missing in Amel_HAv3.1 and LOC408408 (NC_037650.1, location 3,873,175 - 3,879,248) is actually there but just the first 5 out of 13 exons are annotated coding for the 275 N-terminal amino acids out of 1007. The motif/domains which are relevant for the BUSCO gene are located in the missing part. The two flanking loci to the right exist (LOC725925 and LOC410427). The flanking loci left of LOC410425 (LOC102654168 - leucine-rich repeat containing protein) is also missing and cannot be found in Amel_HAv3.1 when blasted, but it is found in a variety of other bee species. However, that's not a BUSCO gene and thus it is not present in Table S3. Upstream of LOC410425 lies a gap in Amel_4.5. LOC410399 encoding 'protein bowel' (NC_007082.3, location 9,293,002 - 9,298,169) is present in Amel_HAv3.1 (NC_037650.1, location 10,465,212 - 10,467,520) but codes only for the first 228 of 543 aa, the other amino acids are encoded by LOC113218532 (NC_037650.1, location 10,429,411 - 10,431,509). These two loci are located on opposite strands and are separated by an approximately 15 kb assembly gap. The flanking loci (left: LOC725099; right: LOC724962) are the same in both genomes, encoding the same proteins. Again, it is unclear why it is among the missing genes in Amel_HAv3.1 but not within the fragmented.

LOC107965534 is present in Amel_4.5 on chromosome 14 (NC_007083.3, location 4,557,379 - 4,558,904) and does also exist in Amel_HAv3.1 (location 5,066,923 - 5,068,437), including flanking loci. Thus, we have no idea why this is listed as a missing BUSCO gene in Amel_HAv3.1.

On chromosome 1, two adjacent loci, LOC100578186 (actin-related protein) (NC_007070.3, location, 20,081,264 - 20,083,399; does not "end" with a stop codon but in an assembly gap) and LOC550723 (ras-related protein Rab-39B) (NC_007070.3, location 20,087,891 - 20,091,693) are separated by an assembly gap in Amel_4.5. The flanking loci can be found in Amel_HAv3.1 on chromosome 1 and code for the same proteins (LOC725348 & LOC724496).

In between these loci (LOC725348 & LOC724496) are three loci (2 protein-coding genes, 1 pseudogene) that are different from the ones in Amel_4.5 and a ~ 2 kb assembly gap. LOC100578186 (actin-related protein) and LOC550723 (ras-related protein Rab-39B) of Amel_4.5 could not be found in Amel_HAv3.1 and seem to be missing.

LOC409086 (NC_007071.3, location 14,655,862 - 14,660,654) on chromosome 2 in Amel_4.5 is present in Amel_HAv3.1 and codes for the very same protein. Thus, it is again unclear why this locus is reported as missing. Also the flanking loci of LOC409086 are present and code for the same proteins in Amel_4.5 and Amel_HAv3.1.

LOC102656620 (NC_007073.3, location 12,451,168 - 12,454,015) on chromosome 4 in Amel_4.5 is present in Amel_HAv3.1 coding for almost the same protein (98.9% identity). The same is true for the flanking loci and the reported “missing” status is unclear.

For LOC410135 uncharacterized protein (NC_007075.3, locatio, 4,077,929 - 4,087,611) on chromosome 6 in Amel_4.5, no significant similarity could be found in Amel_HAv3.1. Flanking loci can be found on chromosome 6 but are about 1.5 Mb apart from each other (LOC724389, location 4,500,753 - 4,655,231 & LOC725997, location 5,787,565 - 5,790,901). LOC725997 codes for the very same protein, LOC724389 encodes only 225 amino acids which is the N-terminal part of the 684 aa long protein in Amel_4.5. LOC725965 (NC_007075.3, location 18,411,773 - 18,414,006) is present in Amel_HAv3.1, encoding the same protein. The flanking loci (LOC107964679 and LOC725935) are present and encode the same protein, but LOC107964679 is not annotated.

Supplementary Tables

Table S1: Blast hits of the here sequenced genomic *mmp1* sequence (PX921700) to the long-read raw data of Amel_HAv3.1 uploaded to the sequence read archive (SRA) database (SRX6817383, Pacific Biosciences SMRT sequencing).

Table S2: MMP protein sequences used to construct the maximum likelihood tree.

Table S3: Metazoan and Hymenopteran BUSCO genes that are reported as ‘present’ in Amel_4.5 and ‘missing’ in Amel_HAv3.1.

Table S4: Full list of Metazoan BUSCO genes identified in Amel_4.5.

Table S5: Full list of Hymenopteran BUSCO genes identified in Amel_4.5.

Table S6: Full list of Metazoan BUSCO genes identified in Amel_HAv3.1.

Table S7: Full list of Hymenopteran BUSCO genes identified in Amel_HAv3.1.

Table S8: Primers designed for this study. PCR products in bold and highlighted in green were amplified successfully, products highlighted in red could not be amplified, products in italics and highlighted in grey were not tried to be amplified as the region was covered by other products.

Name	Exon		Sequence	Expected PCR Product size		
				Exon x -x	cDNA/RNA	genomic
Primers used for PCR				1 - 2	*143 bp*	22.4 kb
MMP_F_Ex1	1	F	CCTGTTGGCGACTCTGTTTG	1 - 4	**456 bp**	32.3 kb
MMP_F_Ex2	2	F	AGTGGTGGAATCATATCCGAGG	1 - 9	1,431 bp	34.7 kb
MMP_F_Ex3	3	F	GGCTTTGGAAGATCGAAGCG	1 - 10	**1,651 bp**	39.2 kb
MMP_F_Ex4	4	F	TGGCGTGTGAAGAAATTGTCG	2 - 4	**335 bp**	**9.9 kb**
MMP_F_Ex6	6	F	ATAACGGTGGAGTTCCGAGC	2 - 9	*1,309 bp*	**12.3 kb**
MMP_R_Ex2	2	R	CCTCGGATATGATTCCACCAC	2 - 10	*1,531 bp*	**16.9 kb**
MMP_R_Ex4	4	R	TGGCCTGACTTCTTTTGGACG	3 - 9	**1,160 bp**	**5.5 kb**
MMP_R_Ex9	9	R	CGAACCACCAGTAAGCTGTC	3 - 10	**1,380 bp**	**10.0 kb**
MMP_R_Ex10	10	R	ACGTAACTAACTGCTCGTCCG	4 - 10	*1,338 bp*	**7.1 kb**
				6 - 9	**645 bp**	*1.4 kb*
				6 - 10	**867 bp**	*5.9 kb*
Primers in introns used for sequencing only						
MMP_F_In3	Intr 3	F	CCCATAAAACACTTCGTTTCC			
MMP_F_In8	Intr 8	F	TGGATCGTACTAACCCAATTACTTC			
MMP_F_In9	Intr 9	F	AATACAACATGCCACACATTTC			
MMP_R_In2	Intr 2	R	CCGGTCATTTCTAACCAAAGTTCG			
MMP_R_In3	Intr 3	R	ACAATTGAAATTGATACGCGAGTG			
MMP_R_In9	Intr 9	R	CGTTTGAAAGAAGAAACAATATGCG			
MMP_R_In9b	Intr 9	R	CCAAACAACGTAACCATCCTCC			

Supplementary Figures

Figure S1: Alignment of Sanger sequencing of DNA PCR products with genomic sequence (JBGNXS010000264.1, bases 495,000 to 534,700) covering Amel_4.5_LOC409868 (Elsik et al., 2014) published within BioProject PRJNA1026921 (Gilligan et al., 2024, bioRxiv). Blue: potential exons deduced from the mRNA sequence HP491739.1 (Elsik et al., 2014); yellow: potential introns; red: potential start and stop codon. Underlined, bold, sequences in italics are primer sequences.

>JBGNXS010000264.1: 495000-534700 Apis mellifera isolate Amel_TP_3
contig_39, whole genome shotgun sequence
TTATTCTGCGATCAGAGCTTCTGGACGATCTACGGGAACACGTGTGTGAAGTCAGTGTCAGAGGGGTGAAGGGAG
AACGTTCCGAGGGCCGAGTCTTTGTGAGGATAGATTTCATCTGGTGATCGGCAACGTTTTTTTTTTTTTCGGGTT
TGCTTGGAATAATTGATTGACGGGATGATGGCCAGGTGGAATGGTATTTTAAGCTGGAAAAGATGTGCCGGAAGT
TGTGC***CCTGTTGGCGACTCTGTTTG***TCGTTTCGCGGGCCGAGGAGGAGGAGAAGACTGGCATATCGCAGACCGCG
GCAATGGTAAGCACTTATTATGATCAATTTTTCCTTTCTCTCTTTCTTTCGCTTTTTAAGAGAACGATTAGAATT
TAATAGTTAAAAGTTTGTGATGTAGATTTTTAGAACGGTTTTAGAAGAGATTTATGATCTAGGAAATAATAATTA
CACGTTGTTGATTTAATGATTAGTTTGATAACTAATGGTACGTTGATAAAGAGATCTGTATCAATTTGGCTGTGT
GATGAATAATTCTAATAAAATCTTCTTTGTTAAAAGTGTATCTAATATCTCTAAAACAATTAGTTATTTGCTCAT
CTATAGATAACAAAATATACAAAGTATCCTATAATTCTTATTTGTAATTCACAAAAATTCACGATGAATATTAAC
AATCGTTCGAAATACAGACAATTCTCTATTATTATGCGATATTTCATCAAATTCACTAAATTCGATGAAGTATCG
CAAAAATTCTCTATTCGAAAATGTGACAATGGGCAACGATGAGTAAAATCACGTTACACAATCGTCGATCGGAAG
TGAATTTTCACGGTTGTTCGTGGAAAATGACACGTTTCGAGGGAACGCAACAAGTTCCAGCTGTTGGAGATCGAG
AGGAAAATACCTGGATGGAAACGATTAATCGAATCGGGAAAATTGATTCGCGCGAAATCTATCACGATACGTGTG
TGTCACGCGTGAATGAGTAATGGCCGTATACACCGTTTTCTTTTGACACACGCGAATAACATTCTTGGGGGGGAA
ACGTTTTATCTCTACGATACTGATATACAGATTACGATTGAGACCAGGCTCATATCTCGAGCACGACTTAGTTCT
ATTAGAAACAATTTTCCCAGTTTATTAGAATATTGAACATTTAGAAAATTTTCTTCGTTGTTCGTCCTTTTTTCT
TTTTCTATTCTTTTTCCAATTTTATTTCATTTCGTTTTGAACATTCTTCGATTTTTTTCTTTTTGATGGTCGATG
ATGAATTTGAATTTTGAATTTGTTGATAAAATTTGTTTGAATATTTTTAGAATACTATTAGAATTTTCTTTTATC
CGATGCATGATATTTTGTTTTAATTAATTTCAATTACTTCATTTTATTGTACTTTCTGCTAATACTCACCAAAGT
CAATTTTATCTTATCGCTATATAATCAGTAGGAAAATTCTTTCGAACGCTGTCACTATTCAATTCTATTCAAGAT
ATTCTATTTAATTATCATTGCCTTTACGATTAATTTCAAAATTCAACAACTCTTATAAAATTATTATTGACTTTC
ACACTTTTTCATGTCACTATCATTATTAATTATCGTTATTTCGTTTTATTTCATCAATATTTAACATCACGTATG
TAATTAATATTCTCACTTGCAACAAATTCTTATCCCTCTCCCTCAGAAACAAAACTATTTCCAACGAATCCATTC
TGATGAATTCTTCCTCGTTGTCTTTCGGTTAACAATTGCACAATTTCAATATGCAACAACACCACGAACCGCATG
AAGTCAGACAGAAACTACGTGAAACCAGAACCGAAGGAATGTCATGAATAATTTTATCTATTCGAAGTATCCTCG
AAGGAGCCCGATGTATAAAGGAGGAAAACCAGAGACCGGTTTCCTACGTAATGACATAAGGTCGGCCAATGTGAC

Commented [AB1]: Primer F_Ex1

GTCACTTGGCTACCTTCGCTGACAAAGTTCAGGTTGGCGCGATGACTCGTTCCTGACCTTCGATTCATAGTACTG
TGCTATTGCTATATATCCCGTTTCAACAGGAAATTCGAAATTTTTTCCAATCCAAAGCTTTGAACTTCTTTTGTT
GGCTTTTTTACAGAGTTTATCCTAGTTTTTTCTTTTCTGGTTGCACCGTTGCACGAGTGATTAAATTCGTTTCTT
TTCGATCCACGATGCTGATCACAAACAAATGTGTACACTTATTAATTGATGTATGTATATATTGATGGCTGATAA
ATAGATTTATGATAAATATTTTTGAGTAATTGATATTGGCTGATTTTAGTGTACACATTTAATTTCAAGTTTTTT
ATTGTGATTTAAATGTGATTCATGAAGATATCTAAACATTTTTCACAGGGAAATTTAAAGATAAATTAAAAACGA
TTGATCTTTCATATTTCTGATGCAGAATTCGAAGCTACATTCAGATTGTATTTAATACTTGGCTAACTTGATAAC
AAAATTCATAGACAAACTTCATATAACATTTTGCCTGTACTTTCAAATAATTACAAATAAAAGGAAAATTGTAAG
AAAAATCTAAAAACAAAGGAACAATTATTAAATTTTGAGTACTGATAAAACAATTTGGACTTTTCATTCTTAAGT
CGATATTAATGTTAAAGAATCGAATGTTAATATTGATTGTCTACTTTTTAAACAAAATTTCCTAAGAAGATACAG
TTTGAAAGTGCAAGATAAGATAATTTTGTTCTACAAAAATATTATTTATTTTACTTCCTTTAATAACAGAATGAA
ATAATATACCAATGAAACAATAATTTTTATTATGAAATTATATATTTCTTTATGATAGAAATATATATCAATAGA
AATACATATCTTATATATTTCTATCATAAAAATATTTCAAATATATTCCTCTCAATTTCATTAAGAAATATATTT
TAGAAAAAGAAAATAGAAAGTTCTATATTACAAAAAAAAAAAAAAAAAAAAAAAAAAAGAATTGACACAATACCACA
TACAATTAAAAATAATTTATCTATAAAATTGTTCTATAAATCTATCCAAAAAAAAAATCTCCTGAAGTTCCAAATT
TATTTTTCAATCAAATTTCACTTATATCTAACTGCAAAAATGAAAAATTCCAAACAACATCGTCAATTTTTCCAA
TAAAGATTTTCTCTATTAAGATTCGCATCTTTTTTCATAATTAAACAATAAAGATAAAACTGATAATAGCACCTT
GGCCGAAACGACGGTCATTGAAATCGCGGTTGTAAAAAGACGATTTCGTGCTTAAGCAATGTAGAAACACGATAG
ATAACAACGATAATTATCTGATAAATATAAATGCGAACAAGTTTATTTCCAAGGTTCGCGACGCGTTAAAAAACC
GAACCGATCTCACGCTCTTGATTACGTTTCATCGATTTTTTGTTAAAAATTAATCGATTAATCGGAGGAAACTTG
TCGAATCGAAATTTCGTAAAACCACATGTTTGCCGTATAAAATAAGGAGCATAAAACGTTAATAAGAGAGAAAGA
TACGATAAAATATTTTTAATAATAAAATTCCTAATATTATCATTTATTGAGAGGATTTAATTAGGAATTTTTAAG
ACTTTTTGTATAATCAATTATTAATCACTATCAATTATTAAATAATAAATTATTTTTATACGATACTAGATTAAA
ATAGATTAGAATTTTATGAATTTTACGTGAAACCAGGATGAAATGCATAAAATTTAGAAGACATGATTTAGATCT
TATAATTTTTTATCGAATCATAAAAATTCATATTGTAAAAGATGTTATTCTTTCATAGAATGACATATTTTCAC
ACACGTATGCCCAACTCTATGTTATGATTGTCTTTGTTTGATTATACAATATAGGATAATTGAATAACCTGTCAA
AGGAAGTTTGTCATGTGGAAGGGATAAATGATGGAATTTCGAAAAAAATTGTGAAACGATCCTTTTGTTGCTTAC
TAGTTGAATGAAAAAAAAAGAATATGGGAAACCATGCAAATCGATATTTGTACAAGCATTTATTTAATTTTTAAT
CAATGAAAATAAGAGGGAAAAATAATATTTATAATTCTTATAAAAAAAAAAAAAAACGAATAACTATTAGAATTGT
ATCATTGACAAGATATTTACCGTTATTTCAATTATTTCCTCAAAAAAGAAACACGTGCGAAGTTTTTCGGATATT
TCGATCGAAACGATCTAATGTCAAACGTCTATTTTTATTCAAATTACAGCGACGCGAAGGAAAACTAGGCTAACC
GTTTGTTGCAAGTAGTAAGAGTCAGGAACGTAGGCAGTGGAATCATACTTACTTCAATCAGACGCATCGTGACAC
GTTTCACTCTTTATAACCCTCTTGCCAATTTCCTTGTTAAAAAATTATTTTACTTGCAACAATTTTTTTGAATGC
AACGAAGAAATTGGATAATATTATCTGTTTAAATTCGCACAAATTTCGATCAATATAATTTAAAATAAATTATTA
TTTTTCTAAACACGTACTTAACTTTTTCCGGATATTATTAATTACAACGTATCTTTATCTTATAATCTCAAAAAA
TATTTTCGTTTCGAAAATAAATATTAAATATTCCTATTCTTACATATTTATAGAAACGCGATTAATTTTAATCTC
GACAAGAATTATAAAATCTTATCGTGGCGAGCTTCCCTCCATATTTAGACCGTATAAACATAATCCAGGAAGAAA
TGCTATTGCCCCTGATACCGCTAATTTATTCAATAATTATTATAGTATACTGTACGACTGTGTAACCGTGATACG
TCACTCGACACGTGCGACGTAATACTTTGTACTACCAACGTAATTGACACGAATTTATGTTGCTGGCAACGTGCC
AAGTCAAACAAGCATGATCGAACACAGTTTCGCGACACGACGTTGTAGTAGAAAATTTTAGAGGCGCCAGGTATA
TTCTTACGTGTATAGGAGATTGCTATCGTAATCCAGAATGGAATTAATTATTCGAGGAAGTAACGGTCGCTTATT
ATTCCACGTAATGGAGATCGTCGATCTTTTTTTTTTATAAATAATTGCGAAACGAAGATATTATAGAATTTTTGT
GAAGTTTGTCTTGTATTTTAGATAATAGAATTTATTCTCTGATCGATTTTAAACTCTAATAAATTTGGTGAATTT
GGATAGAAAGTTTATTATTTATCCTCATGTTATTTTATCTCCATTTTTATTCCTGTAGAATAAAGAATCAAAAAT
CTAAAATAAAAATGCACGAAAGTGAGGATAATAAATAATTGTCTAATTAATGATTCATCGATCAAAGATAAAAAT
GTCATCTAGCTGACATTCAAATAGAAATAGAACTTGGAAACATTGAGCACCAATTAATATTCAATCAGATTATCA
TTTATATAGATATCATCCAGCAGAAAAACCTAATAAAATATACAATTATTGACAAATCCTGGAGCAAACAAAGTT
ATAATATATAACATAATAATCTATCCATTACTTGACAAAATCTTCATACAAAAATCTTCATTACAAAACCTCACT
GATCAAAATCAACCGTCAAAATTATCCGTCAAAAATCTTCAAATAAATAAATCATAATTCAAACAAAAAAAAAAA
AAAAAGAAAACTTACAGATTCTTTGTTTGTTCCGATTTTTCAAAGGGTCGATCGAGGGGAAAAATCGATTCGGTA
TCGGGCAAGGTGCCAACTAATCGATTCTCACTCGGCTCTCTTTCTCTCGCTGACGGATCATACTAGGAAATGGTA
AATAATGTATGTGCCTCGACACGGAAATACACACGGCTTAGGATAGCGAATGTTGGCCTTCGTCGTGGCCGATCC
GCAGGCAAGACACGATCCGACCGTCATCGGAATATTAACGATCGTTTCCATAGATATTCTCATCGTCGTTCCATA
CCACCGATCGTTCGCTCTTTTACGTATCCTCACTGTCTGCTGCTTGACAGAGAGAAAGGGAGAAAAAGAAAGCGA
GAGAGAGAGAGAGAAGAGAGGAGAAAGATTTCGAGATGTATGCACGCTCTTCCCCTGAGATGAGATATGATCGAA
TCGATTAGCTTAATTAACGACACCTCTGAAAATATTCGTGAATGGAAGTTTATAAGAAGTTTTTGATTAGTCATC
GAGTGGTTCTACGTGCTTTCTTTTTTGAAATTTTTCGACTCGTGGGTGGTTTTAATTTTAGGAAATTTTAGAATC
GATTTTATTTTTAGGGAAGATCGTCTTCTCTAAAATTGAATTGTAATAGAAAAATTATAATTTCGAGGTAATTGG
AATTAGAAAAAATTTTATTAAAAAAAAAGAAAAAACTAAACTAACTAAAATTCTGTACGTTTAACGCGAAGATGA
TTCATATTGAAAGATTATAATTCTATGGAATGAGATTGTACAGAATTAGAAAGAATTTTGCTGCGAAAAAAAAAA
ATGTTTTTCGTCCGAAGTGAAAAAAAGTTAAGTGCTTCTGTTTTAATCTGCGTAATCTTTATTAGGAAGAGGAAA

AAAACTTAACACAAAATTTGCTTCCTGAATCCTAATCGCATTCATCTAATACTCGGCTACGCATACCAATTAAAA
AAATCTCATCAGAATTCTACTTTCAGTATTAAAATTGAACGATCGATTCTTCGGAGAGTTCAAGGAAGACATGTT
CTCGCTTGTAACGGACACCTTATCTTACTTCCATCGCGACAAAAAGAAATAGAAATGTAGTCATTACGTATGCAT
TGCTCGTTTGATCTTGAATACCGTTTAATACGTGCAGTATCATCATTGATGGAACAAAGGATAATTTACGATATG
ATTTGAATAATAGAAATAGTATCACAAATCAAAAGTAGAATCTCTCATCGTGGATATGACACGTCATGATATTCA
GTATCATTATCATCATTTTTCAAATTGAACATAATCTAAATCTAAAAAAATCTAAAATTAAAAAAAGAAATCTAA
AATTAAAAATTAAAAAAAGAAAAAGAAAAAGAAGAAGAAAGAATTGAAATAATGAAATAAAATCGAAACATGATC
GCTAGATTTTTAACTCGATATTAGTTATGTAAAAATAATGCATAGTAATTCAAGCATCAAGGTTCGTGTAATTAA
CGAAATAATTATTTATATTTATTTTTTCCTTCCAAATTCTACAAATAAATAAAAAATAAATAAAAAAATGTGCCT
TATTTTTTTTTTATATTACATTTAACTTTTCATTGTTAAGATAAACAAATTCTCATTATTAATGATTAACAAAGC
ATCATCAGTTATTAGAATGCAACAAATGAAGTTTTCTTGTAAATTATAAATCAGCCTTGAACATTCTGCAGAGAA
GCGTGTAAATCAAGGATAATTCAATCATCATTCGAAAAGATTGTAATAACTATTTAATCCTCTCTTTAATAAAAT
TTTCCTCGACCTGCACAGCTAATCTGAAATGCAAGCTTGAAAAATTTAATAAAGAAGAATTTTCAAATGATAAAA
AAAAAATGTTATGACAGGAAAGAGATCGATTCGAATTTTGCGAACAATGCGACTAATCAAATCATAAAAATCAAT
CATGCGATCTTCCGTGTCTTATCAGGCTTCGCGGAAGATGCTTACCAATGCAAAGATAGCTATGCTCTTGGCAAC
GGCTTATTACAATTACTAATCGCACTTGATCATTACTCGACGTGTCTCGAACGATTAATTAGACGCACAAGTATA
ATCGCATCGTTGCGCAAAATTTACTCGCAGCCAAAGAAAAAAAATGATTATTAAATCACATCGATTTTAAACAGA
CATAAATATAAATCGTCATAAAGATATTCTTTTTACGATTTTTAAATATCCATTTTTATCAATGTTATTCTGCAT
TAATGATTTTTTTTTTTTTCGAATGCATTTAAATTATATAATCTCGCCTATATTGCTATTCTTTTCGATTGACTT
GATGCTAATCATGAATAACGAGAAATGTTATCGATGTTTCTCTTGAAATTCTTCCAAAAGAAATTAAGATTTTCA
AATCTTGGAAGAAGAGTTCCAGATTTCGAATTATATCAAAATTTCCATAGTTTTGGAAACTTTGAATCGTTGTTT
AGAAATTAAGAATCGAATGTCTATTGCTAGATAAGATTTAACTCTATTAGAATCAATCTTTGAATGGAAATTTGC
TCGGGTACGTGGGGTCGCAACTCCCCAAAGGACTCACGAAGACGACACTTAAGATCGTTTCGTTAGTCCCCCCTG
CGGGAATATCAAATAAACTTTTAGAAAAATTGATTTTTTTTTCTATTTATTAATTAGTTTTTTGACATTTTTATT
CATCATTAATTCATAGTTTGATTCTAATAATTTTACAAAAAAATAAAAGAAATAAAAAATATAATTCATCATTTTT
TATACTGTATAGAATATTTGTTGAATATTAATATCTTAATATATATATGAAATTTGTAATTTTAACCATTTTAAT
CGATAAATGTCAGTCATTGTAAATTTTATTTATATAAATAAAATTTTTGAACAAATAAATAAATCTACCAAGCGA
TTTTCATGCATGTGATTCACATTCAAGTTATACAATGAACTCGAAATATAATAAATGTAATCGAGAGAAGAATGG
TGAGAAGAAAATTCTAATTCCTCGAATTCGTTAGCATTTTCTTCGAACATATTAATATTCATATCTTGTAAATTA
TATGAAATTATTAGTATTTGATGATTAGATTATTAGATTGATAAATTTAGAAATAGAAATAATTAATTTTATAGA
ATTTGATTATTGATTATAATATTTATGAAGTATTTAAATTATAATAAATTAATAAATTATAATCTCGAACATTGG
AACAAATATAACTTGGAAATAATTATAAATTTTATTTTATTATTATTATTTAAATTTAGAAGAATACTATAATAT
TTTCTTTTGTTTTTTTTTAATTCTGCAATTAAATGCAAAACTTACAATCTTAATAATAATCTTGTTAACAAATCT
TATTTACAGTATCATTTACGCCATCATTAGAAATCAAATTATAATTAACGATGAGAAAAAAGGAATGCAAATAAT
ACACAGTCAATTATTATTTTCTTTATAATAAAAAAGTGTTTACTATCTGATAAGTTTTCGAAAAATAAGAAAAAT
ATTTTATTGCACAACACGTTAATTTCTCTATTAATAAATTAAAACCAACGTAAAAAGGTTTCAATTTATAAATTA
TAATATAATTAATTTATAATTATCAAATTTTCAAGTTAATATTTTGTTATTTTACAATTACTTTTAAACATTTTA
AATATTTATATACAATTAATACTTATAATTAATTTTTAAATTTTAGAAAATTAAAAACTTGAAAAAAAAACAGAA
AATTTAAAAATTGTAAAGAGAAAGATTTCAGGACAAAATATTTTAATGATTCCAATGAATGTTATTATATTTTAT
ATCTTCTCACAACAAAAACTTTATTTTGATTATATTTTGATTGTGATATCTTTCTCACGTAAAATAATATTCAAT
CCATTCATTAAAACTAATTTCAACTTTTTTAATTTTATAATGCCATTACTAATTGCGATTATCTACGTATAAATA
AACGTTTCTTGTGCATTCTAAAAATTATACGTATCCAATTACTTTTAAAGTATTTAACTAGCCCAGAAAGATAAA
GAAGCTAACTGATCTAAATAACAATAACTAGCATTAATATTCAAACGTGCATAATATTCAAGACTTTATTACAAT
TAAATGATCACTCTACTTACATCATAAAACGTTCCATAACCGTCCCTTACATCTGACCAAAACCCTACAGAAATG
GAACAATCCCATTAAAAGAAGAACAAAGAGAACAATCGATGCAAAACTCTGGTTCTCCATTAAAAGATATTTGCC
TGGAAAACGCTCGATTAATTATCCGATGGAACATCATCTCTCTGATGTGTGATGTCACCAATATATCGTTCACGA
TAATTCTTATTTGCATCCCGTGACATTGATGAACGTAATCCGAGATCTTTGATACGTGATCGATTCGAGAGAAAA
GAAAGATTCGATACGCGTCTCGATCGACCTCCGCGATACGAAGCGACCCAACACTTCCGGTGTCGTTGAATCAGC
GATTTTTTTTTCTTTTCTTTTTTTTTTGCAAACCGTACTGGTGACTCATTGTTATTTTATCGCCTGTTTTCTGGA
CGTCCGATTGCGACACCGTTTATATCGTAACTGGGGTCGTAGGTCGGCAACCTCGAGCTTTTAAAATATAAACAC
CGGTGACCGAGAAAAGAGGAAGAGGAGTGAATCGTAGTTACGTGTGGTTCGTATAATTCCAACTGCAATTATAGG
TACACGTTTATTTTAGAGTAAAAATCTAGAGAGCTGCTCTCGAAAGTATTTCTATGTTTGTTTAAGAATTTTTAT
TTTGAGGATTTTCATCTTGAAGAGCTTACTTTTAATGGAATTTAGATCTATTGTTATAGATATAGTTAGATAGAG
TTTCTCATATAATTCTCATTATGATTGGATTATATGTATGAAGAATAACTTCTCTCGAGAATTGTTCTAATGAAT
ATAAAATAGAAAAATTTTAGTTATTTAATTAATTGGATTTTTTCTATTAATTTATAAATTCCTAAATGTAAACTT
TTATTATTCAAAGGACAATACGTTTTACTTTTAATAAATTTTTTAATAAAATGTAATATATATATATATTTAGTA
AACTACATTTTAAAATTTCATATCTTATATCGTACACAGTATCAAGATATAAATAGTATTTCTTCATATCTTTTT
ATTTATTTTTTTTTTCAGTTTGAAGCAAATATAAATGGTTTTATAACTATCTTTCGAACAAGAAGATAAACATGA
TGTATTGATAGATATGATAAATTCTAAAATTCAGAAATTTCATCAATATATAAAATACAAATTATTCAATGTTAT
TTTTAAGTCACTCACAATTCAATATCTTTTTCAAAATGTTATTAAATTTCTTCTTTTTAAAATAATCAAAAATTG
TATCATAAAATCCTAATTTTTGTCTGAATTTGTGCAACTCTCGATTCACTTATCAAAATAAAAATACGATTTAAT

CGATCCAATATTTTATAATATAATTATAATATAATTATATACAAAATTTTCGTTAAAAATTTCGGATCATCGAGT
ATTCACAGGTATTTGACCCTAACTGACCTTACTCGACCCAGCTAACGATTAGGGTTAGGTAAAAGGCCAATTTAA
AATGTTGCGTGCACCTGTTCGTGGAATAGTTTCAAGATAACAGATTGAGGAATAATCCCGAATCAACGTTCGTTT
TTCTTAATATGCCGGTATACAGAATTAAAATGTATCGCGTGGAAGATTGCAGGACTGGCACCGTATGATTTTACG
AGGTCGTATTGATTCTTTTTTCTCTCTCTCTCTTATTAAAATTCAGTGTCGTTGACGCAATAAAAACGCGCGCTT
CGCCGAAATTAGTTACGCGAAATGATGGAGGAAATTTTTTAGGATTCGTTATATGAACATTTTGCAAGATGAGAA
TATTTAATGATTAAACGATTTGGAGTTAATTGATATAATTTTTTTTTAAAGAGAAATAATGATTAATTGAATATA
ATGAACAATGCAAGGTGTATTCGAAGAATTTTCCCTTCAATTTCATTGATTTTGAAGATATCGATTACAGATTTT
TAATTTAATTTCGATTTAAATTTATTATAGATATAGAATTTATCACGAATTGATTTCAAAAATTATTTGCATTAC
ATTTCCACGCGATACAATTCAATTGTTCGAATCAACGCTGGCAACGTGATGATGTTTACAATGAATGAAAGATG
ATGACCGTTGAACTCTACTTCAGTTGAAGAAAAATTATATTATACAATTTTCTAAAAAAGATTCAAGCATAGATA
ATCAAATTTTTCGAAACATTTTACGCTTGCCATCTTCGTTTAATAACAGATAAGTAGTTAGTTTCTTTAAAATTC
TAAAATTTCTGTATCTCAAATCCCTCTTTACTTCAATTAATCTCTTTTCAATGATAAACGCAACTCACCTATCTA
ATTTCACTAAAACAAAATTAATTCTCAAAATTTCTAAAATTCTCTAATCAATCAATACAATCCAAATCAAAATCC
AAAGATCAATCTCTCCCTTAATTTCACTTAATTTCATCCTTTTCCATACTCTCATCCCGATAGACAGTTGAAAGA
AAAATCGGTACCAAAACTTAACAATACTCGGTCGACAGAATTGCAGTGCGCAAAAGATATATCGATTGTTGATGT
CTCTTAGAGAGAGAGAAAAAGAAAAGGAAAAGAAGAAACGTTGATACAATGGCAAGAGTACGCGAAAATATATTG
TTCAGCGATACAAAGCTGCACGGTGGACGATCGGGGATCGGTTTGGTATTCAAGGAACGGCCGGCAGAAGATGCG
AAGGCGTTGAATACTGATGCCGTGCGCGTCGACAAAGACCTCTTCAAGGTCGGGGTCGCTACTCGATACCTCTTC
TGCACTTCTCCTCTCGAGTAACAGATCCCATCCGCTGCACCGTTAAACGCGATGTTGTTCTCGGAGAGGAGAGGA
AAAAAAAAAAAAAAAAAGAAAAAAAAAGAAAAAAAAAGAAGAAGGAACTTTTATATGTCACGTATAGAACATGTA
AGATTCAACGATCGTTTTATTCTTGTTAAATATAAGTGTAAGAAGAGAAAAATATCCGGTCACGTTAGGTACTTT
GCATGTTTTACGTTAAATTGCATAAATTGAGTAAAAAGAAAAAGTCTATTTTTAACCCATTATTTCTGGGACCAA
AAAATGTATTTTTGATTATTATTCTAGGATACGGTGGTGTTCTTTGCGGTGGTTAAAAATAAGACGTATCGTAAA
GTAAGAAATTGTATTTTTAAAACTGGCGTACGAAATACGAATACTTGAATTGATTAGATAGAATTTGTGATCGAC
CGGTGGAAGATAAACAAATATTTTCATTTATCATACAAATGATTTTGTGAAAAAGTCATAATTAAATACAATTAT
ATATATATAGATAAATTATAATATAACGCAAGAACAATATAGAGAAGAAAAAAAGGAAAAATAATTAATAGAAAT
ATAAAAAAATTCTAAAGGAAAATGAATTAAATCATCGTGCAAATTCATCGTGTTAATCTATTGTATTTTTTTAAC
GAATGAAAATGATTTTTAATTAAAAAAAAAAAAACGAATGAAATATAAATAATAAGAAATAAAATAACGACACGGC
CTTGTAAATGGTGTGGATATAACTGCGAATACATTCGATTTTCGTACTATCGATCTGTACAAGCAACTGAAAATC
AGGGTACGTCGTATCTGTTCTTTCCCATGCACATGCGATAACAAGGAATAATGTCGTTGACCTAGCCATTCATGT
TAAAACGAATAAAAAAGCACGTCGATAAATCGATCTAATAATGAGCATGCACTTTGAATAACATTCATCATTATA
AATAACGTAGGTATTTCGAATAACGAAAAAAGAGGGGGGAAGGAGAAGAGAGATTTTATATTTTGTTTGAAATAT
TAGATTACCGTGAATGTTTAAAAAATCGAATGAAAAAAAATTGTGAAAGGAATGAAAAAATAATCTACAATCTGT
GCAATGAAGTTTGAACTGATGATAAAATCATTCTGCACGTGATAAAATTTAAGCGAAACCAACGCTCTAATTACT
TTCAATTGCGTATTTTGCTTTTAAAATGGTATTTATTAATTTTTTTACATTTAATCGAAAAGATATTTTTCGTAA
AATAAGATTTTGAATTGACCTATTTTTAAATAGAATTTATTCTACTTTCTTCTATTCTATTTTAATTGAGGAAAA
CGAGTTTTGGTTGGTCGATATTGGATGTAAAAATATTCCAAGCATTGTAATTTATTCATCCAATTACTCCATGTT
CTTTTAATGTCATTAAATTTAAATAAAAATGTAATTTCTTATATGCTACGTAAATATTTCTAAAGACTGAGCTAA
ATTTCTCATATATCATTTCTTAATTTCGTGATTGGAAACTACATTTAATTGAATAATGATTCTCGTTATTAATTA
GCACACATACTTTGAGAAATTCTAAAATTTCAAATCCTGAACTGAAATAATGTTTACTTACTAACTTTCAGAATT
CATATAATTTCTATGTAAAATAATCACATGCAGAATATGATAAATCACTTTTTTTTCCGAAGGAAAACAATTTAA
CAAATTAATAATCTTGTAATATATTTTATTTTAATTAAAGAAAATATCTTTCTAAATTTTAATTAGAAACAAAAA
TCAGTAAAAATGAATTTATAAAATAAGATATTTACAAAAAATTAAATGATGATTAATAAATAATTATTCTCAAAA
TTGCATTCAATTAAAACATATAATTTATTATCGTATTATGTATTAAAAATCTACGAAAAATTTTTATAAGTTTTA
TGCAAAACTATGCCTAATAAATTTCCATGAATTATAGCTAAACTACTGCTGAAAATTTCAAAAAAATACATGCAT
TATTCTTTAAAATCACGACTTGTATGTTACACTATCAAACGATACGATACATATATACATTGTACAAAAAATATG
TATGAAAGTTTTCATTATTCTTTTTCCATTATGCGTTATATTACATTTACAGTTGTATACGCATACTTTTCCTAT
GTTATCAGTGATAAGGTCGCGATAAGGTCCCGCCTGGTAGACAAACCCAACTATCAAGAAGAGACATTTCATTGC
TTCGTGCCCTCAATTATCGAGAAAATTGCGTGGCGAAATTACACGAGCAATTAATAATTATATAAAATTAATAAT
TATACTTAACAAATGTTCATTTTGCATAATAATAAATAATGTTTATTTCATTGTAATAGGATTTTATATAATGAA
TTTTATGGATGAAGAAAAAATAAAACCTTTTTTTTTAATTACACTTCCTTCTGCAACTTGAAGAATAAAAGAATA
AAAAGTTTCTATTTATTTGAAATTTTCGATTGAAATAAAAATTACAAAGAAAAAGGGATAGAAATATCGCAAGAT
TTATGTCCCAAAAGAATCGTGTATACCATTTTTGCGTGATTTCAAGAAAGTCACTTTACAAGTCCATAGTTTTTC
ACGATGAACTACTGCTATGACTTCAATAAATAGTACGAAAAGGAATAAATATTAATGCATTCATTTGCATATATC
GATATCGTGACGAATAATTTCAAACGAGATCTGATAAAATTAAATTTTCATCTTATAAAAATATCCCGCAAAATA
TTCTTTCTTATCATCTTGAACTTGCAATTTAACTTTCCAACGATCGATCCACTAAAAGTTCGTAAAGTAAAAGAA
ATAAATGTGGAATCGCTAGATCTCTCCATTAAAAAAATTCAACTTCTTCATAGCGTGATTTCGAGAGATTCTTAA
CTTAAAGTCATTGCAAAATACGTAACAATCCAACCGTTCTATTTGTTTAATGCGCAGAGATTAAAAAGCTTCTAT
TCAAGATCCACTCTCCCACTCGAAATCTATCCAAGCTCTCGCGATCCCCCAAATCTCCGATTTCCACGGAGATAG
TTAAAAGTCACGGTTTTTCACGCTCGAGAATCGGAAAATTTGCATGAAACGTTTGGCCTTTCAGCCAGGTGTCTA

AGCCTCTATCTCTTCTAATCGGAAAGGTCACTGGTTGATACATCGGTCGACCCAGTTACCCACTTCCTTCCAGCG
CCGTTAAATTTATCAAGCGGTTTGCCTACGCAGGAATTCCGCTTAAAAATCGATCTCCTCATCCGGCAACAAATT
TCACGCTGCGATCCAGCTACCGGGATCGATAGGCTTCAACCGTTGTTATTACCGCAACAGTAAATAGGTTGTCGG
CCGGTTGCAATTGTTTTTTTTTTTTTTTTTGATACGGATCCATTATCGTTATTTTTTCTTTTTTCTTTTTTTTACG
TGAACACGCGCGCATAACGATAGTGTTGAAGGTAGATGGGCAGGATAGATAGAGGGATCATTACGAAACAGAAAA
CAGCGATTATTAGACGGCGTGTGGTAACACGAAATCCGTGCCAGTTTGGAATCGGGTCATGGAAACGGGGCATGC
GTCATACCTGATAACCCAATCAAAATGTCAACTTTTCGACAGGTGAACGCGATACGAATGCGATCTTGTGGATTT
ACGTGGATCGCGAGCTTTAAACGCGGGGCGAATGACGAGCTTTGCGGTTGTCGGTTCGTTCACCAAGTTTTGCAA
ATTGTCTACTGTGTTGATGGAATGATTCTAATTAAAAGAATCTATGAATGGATTTATATCTTCAATTTCTAAATT
CTTTATGTTATAATTATATTATAATATGGTAAAAGTCATATATGTTTTTAATTTATAAGAACTATTATAATTTT
TATCTATGAGCATTGATGATTTTATCTATGAACAATGCTCGATTAATTTTTAAACTATTTATTTTTCAAACATTT
CAAATTCTATTGTTTGACTATTTTATCTAATGTAATATATTCTGAAAGGTTCGTTCACCAAGTTTTGCAAATTGT
CTACTGTGTTGATGGAATGATCCTAATTAAAAGAATCTATGAATGGATTTATATCTTCAATTTGTAAATTCTTTA
TGCTATAATTATATTATAATATGGTATAAAGTCATATATGTTTTTAATTTTATAAGAATATTAGTTTGAATAAAT
CTATTATAACAATGCTCGATTAATTTTTAAACTATTTATTTTTTAAACATTTCAAATTCTATTATTATTATTTGA
CTATTCTATCCAATGCAATATATTCTGAAAGGATAACATTTGAGCATATCTCAAAATGGAATCCAATTTCTCTTC
AAATAATACAAATGGATTATTCGATATTTGAATTGAGACAAAAAATGTACAAAAAATAATATTCTACATTATTTT
TTGCTATTTATCGTAATAAATTCGTGATATTATCAATCAAATTATAAATTCCAGAAAATTGTTTAATTTTCTAAC
TGTATTAGCAATTTCCATAAAAATTAAACTCGCCAGAAGAAATACACCAATAAAATGCTGAAAGTTGGAAAATCT
ACACGGTCGATCCAGCGAAAATTCTCAAGGTTGTACGTTAAACTAGCAACAGAACTGGTCTGTAATTTGAAATTG
TATTATCGTGTACCCTGCACGTTTCTCAAGGTAGCTCGGGTATAAAGTCTCGTGCTTTACCCGAGAACGGCCAGC
CACAGGTAATTACGTCAACGGTGACGATTATCGATTCGCAAGGATCGTGAACCAACCTCGAGAAAAGGGCCACGA
TTACGTGGCTTGTAATCGACAAGCTCGAAAATCGTCTAAAATTCTAATTTTCATTTTCAGGAAAATACATAAATT
CCAAAAGCAAGGGTTAGTTGGAGCCTGACAACTTTCATTTTTGAGTTTTCGTTTATCTTAACTCCCTTTTAATCT
TTCTTATGATGAAATATCATGATTCTGTGGAAATGGAATTTTAAAATACTCCAATTGCTATCTCTCAAGCATCTC
AAATATATTATAAATAATATACACGATATCTTACTTTATCTCGATTAGAATACTTCTTCAAAGAAATTATGAAAA
AAATATTTTTGAGAGAGTTGTTTCATGCACACAAAAATCATAAAAACTTTGGATCTCCTCATCAATATCAAGACC
TCCTTACGTCAATCAAAATTTCGAAAAACTATATGAATTATCTCGATAACCTCTCGATTATCTCAATAATAATAT
TTCCATTTATTCCCATTTTATGACGAAACTAAACTACAACATTCCATCCCCCTCGCCAATAGGAAGCGAATGATA
CCAATAATAAGAAGATCTTCCCAGAAACAAGCCACGTGAGGTGGAAAAAAATGTTCTCGAGCAATTCTAGACGTTT
CATTTTCTCTTCCTCGTTTCGACCGTGAGGATCGCGCAATCGCGATTCTCTTCCTTCGATTTTGCTTCACGTGTA
CGTACTCGCACGTGTAATTACAATGCAATGTCACAATAATTTACGACACATGTCTTCTAGATTTTTATTTTTCCT
CTTTTTCTTGTTTTTCTTTTTTTTTTTTTTTAATCTAACATCTTCTTTCAAACTAATTCAAACTCTTATCGCCAA
AGACGAAGAAAACGATAAGATTCTTTAATTATTACTCTAATTAGTCGTTTGGGAATGTTAAGATGGATTTGTTAT
TATTCTTTATCACGTTGTTGTCTTGAACACCTTGTATCCTGATTACAGATTGGAATTGATTAACTTGCATAACAA
CATGGGAAATATAATTTTAATTTAATTAATCGAACGTGTAACAAATAAAATGCAATCACTTTATGCTCTGTGTCA
AAAGACAAAATTTCATTCAGGATTCAAGGCATTCCGTTATATAATCTAAGGATTTGATACATCTTATTATTTTCT
CTTTGTGAGAATTGTTTGTAAAAAAATTAATCAAATTTATGTTCTGTGTTAAAAGACAAAATTTCATTCAGGATT
CATTCCATTATATAATCTAATATATTTATATATTTATATTTATATAATCTAATATGATTTCATACATCTTATTAT
TTTCTCTTTGTGAGAATTGTTTGTTAAAAAAATTAATCGTTAGCAAATTAAAATAATATCGTACCTTGTACGTAA
GAGATATCGAAGAATTATAATGATAGAACAAAGTGGTTGGGTACTAATGCGATTAAACACACCCACGATTCGTCA
CGTTGCCTTGAAGAATTCGTTCGTCATAAAACAGAAAGACCATTATAGTTAACTTATCGTGTATATGAATTATCG
TATAGATTGTACGACATCACGTATGATTATATTTACACACAGTCATTTATAAAATATATGCTCGAAATTCAGTTT
CTTTTATATTTTTAAAAACCTTCAAAAAGTTCTTATTCGATCAATATTTCATTCGAATAAGAATTAATCAAAATA
TGATATATGATATATCAGAATTTTAATATAAAGTATATCATATTTTGTAAAAATTAAATGATAATATTACGAATA
TGGAAGATTTTAAATAAAGATTTCTGATCATATTGCAATGTTTCTGCAATTGTACTTCTTATTTTCAAATTTTCG
ATTGTACTTTAAATTCGAAAGATATCGTGGCAAAATTAACTTCAATGACCAATTCTTCGTAAAAATTCATAAAAA
TCATTATTATATCGAACACGATATGCACTCGAATACCACAAATCGAAGTCACGAGATTAACTGGATGACGATCAT
GCGCGTGTCTCGACTCGCGATCTCTTCGCTTCCACCCACGCCGGCCAGTATTCGTCAATGGCGAATACGAAATCC
TTGGATTCCGCCAACGATTTTGCCCGGCGTTCGAGTCACGAGGAAAAAAAGAAAGAAAAGAAAGAAGAGGCACGA
ATGATTCAGAAATCGTAGTTTCCTGGCTCGTACGTTCGCCTACATCGAAAATGTACACTCACGTCCATAAGTCAT
TGGATACTCGTTAATTTATCGATCTAATAAAATGTATTCCTTTGATTCAAAATTGAATACAAAACTGATCGAAGT
TTTTATTCAATCATTAGAATTTTATTTTTGATTTTATATGAGCTTCCATGAAAAATAAGCATTATATTTTATCTA
TATTAATTGCTTTCTAACACTTACATTTTATCCAAATGCTGTTTAAAGATTTGTCAAAAATATTCATAAATATAA
AATAATCAAATTTTTCTCGTATAATTTTCTTAACATTCTTGCATTTTCTTTTATCTTATACAACTTTCCAAAAGT
GTATAAGTGTTTCTCGTGAATATATTAATTAGAGGCTCTTGTTTCGATCGTATCTCTGTGAACGTGTCTCATAGA
ATGACTTATGAACAGAAGTGTATTATACGATCCTGTTACGAGGGCTTTGGAAGGAGTGGCCATTCACGCGTGACA
CACGCGCTCTCGCGAGTACGCGAGAGCTCACGTTCATTCATAAAAATCACTCTATTCTCGGAACCAACTCGGGTT
TATTACGAGATTCGTAAGCCAGGGAACCGTGATCCATGTTTGAATCATTGACACCGGGTAAAAAAGCGATAGTTA
TCGGTGTGGACGAGGAATTTTGGCCGGAGGATCGTGGTCGAAGCTTCCCATTCACGAGGAAAGAAATTTATTCGT
AGAGAGATTTCGTGGAAGAATTTTATTTTTTTTCCGACTTTCGATCAAATTTTTGGGGAATTTCGTATCTATTTT

CCGTGACTCAAGATATTTCCAAGAAATATCCAAGATTATTTTATCCCTCGAATTCTTGGTAATTAGAGATAATTG
GATTATGATAATGAGAAACGTATGGAAAGAAATAATTTTTTGTACGAATATTTAAAAATATGTTTGTTATTTCTT
TTTTTTTTTTAATTCTTCGAAGTATAAATACTTTATAAATCAACAATGGACAATTTTTTAAAAAATAAAATTACAG
TAGTTCTTTTTTTCTCTCTCTCTTTGTCTTTCTCTATGCAAATCTCGATGTTTATTTTCGTCGATCTTACATTCA
TTACATACGAGTCCAGATCCATAAGAGCAGAGCTAATTTACATAAACGTTCCCTGGAGCTCGAGCTCGATGCACT
TGAAGCCATAATTTCGTGGAATCCTGGACGAAATCCTTCTACGTGTTTAGAAAGGAATGCATCACGTTAAAAATA
ATACTGGAAGAATGGAATAATCTTGTCTTATAATACAGGTTTTAAATTTCTAAGTTAAGCCATTTAAAAAATTGC
TTTAAATTCCAAGTATAAGTTAACCAGTTTGTTTTTCTTTTTTTTTTTTCAATTGATGTTTCAATAGTGATCAAT
AGTGAGAAAAATGAAATCGAATATTACATTAACAAATATTTTAATGAAATTATTTTCATTAACAATTAATCAGAA
AAAAGAATGATTAATTGAAGATCTGTTATCTGTAATTACCAACTATTTAATAAAACAAAACAGCAAAAATTGATT
ATAAAAATTCTTTCTCATTAACACTGCACATTATTTCATTTTTTCTACCGTTAAAATTTACATTATCGATGTACT
TCATCTACTCCACAAATATTGCACGTCCATTTTTCTTGTATCTTCGAGAAAAACAGGTTTAAAGATTCAATGGTA
CGTATAGATTCAAGGATAAGCGATAAATAAAAATATAATTTTCATAAATTCATAAAGTAGATATAAATGTGACTT
TTATACAACATGAAGAATTATTGAAGAACTTATATATATATATATATATATATATATATATATATATATATATTA
CAATACAAAGACTCAAATTTCAAAAAAAGAAAAAATGGATATTCGTGGAACGAACCATAACCTCTCCAAAACGAT
ATCTCCACGGAATAACGAAAATATCGTATCAAATTTTCAACAAAATTCGTTTTTCTAAAATCCATCCATTAAAAA
TTATGAATCGAATACCATAAATTTCTTCAAAAATAAAAGCACAAAAAGTTTATTTCGATGAATCATCATCGTTCA
ACGAGACTTCCGACTTCGAATCCACGTGATGGTTCGAGTGCAGCATAAACGCAATCCGCACTTCGATTATTCGAA
TGAATATCTCTTTTATCTGTATCGTGAATCACGGCATAACTTCGCGTTTACGAGGAGTCTAGTCGAGGCTAGGAT
TACGTTTTATTGCTCGTTTCACGGTTTATTGGCGTTTGTTCGGCGTTGAATTTAATGTTATTTAGCTAACAATAA
AGAGGTTTAAAAGGACGCGCAATAATGCACGTTTATCGAGTGTAATTGTCGAATACGTTCCTCATTAGAAATAGA
TACTAATTCGTTATTTGAAGAAGATTTAAAAGAAATACTTTTTTCTAGAATCTTAAGCGTTGAAATGATTGACAA
AGAAAAAAAAATCCATCCAGATGAATAATATTTCTTATTATTATTAAAACTCGAAATACCACGGATTTCTTTGAA
GAATATCATTTATCATTTTTAAAAGAATTGAAAAAATTGAAAAAAAAATGAGAAACAAAGAATAATAATTTATCG
AATGATTTTTTACGTCTTTATTATTCGCGAGTTTTCAATGAGATTTCTTGCAAAAATTAACGAATAAACTTCATC
GTTCGTACATTCCTTGCTGTGATTCACGAATTAACAAACTTCTACCTGCTTGATAAACCGAAATTCACAATGATT
CAGCGTAGAAATAAGAGACAAGGAAAGCGGAAACACGTACACGCGGCTCGCTTTATTCGCCAGAATATTAATTCC
CTTTACTCGCCGTGGTTAAATAACTGTATCATAGCACAATATAGTCGCTAGTCGGGTCAATCCCCTCGGACATCA
CCATCTGTCCCATTTATATTTTTTCAGCGTGCGAAATCTAGCGGTACGATCATCGATCGAGATACCACGAACCTT
TTTACGATATTTTTAATACCAAAAATAATACGAAAATGTCAGTTGAAAAAAAAATGTTCAAATAGATTCGATATTT
CTTCGATATAACGAATTATAAATTAAAAAGGAAAAATTATTAAATAAAATTACTATTTATTATCTGTTACAGAAT
TATCTCTCTCAATTTGGATATTTACAACCTATGAATCCAACA***AGTGGTGGAATCATATCCGAGG***AAACACTTTCT

```
AAAGCAATCTCGGAATTTCAAGCTTTTGCCGGATTGAATATAACAGGTAAGTCTGATATTTCAATTTTTATAACA
      ATCTCGGA-TTTC-AGCTTTTGCCGGATTGAATATAACAGGTAAGTCTGATATTTCAATTTTTATAACA
      ******** **** *******************************************************

TATTTTTTAACATTACAGTAATTAAAATTTACAAAGAAACGATAAAAGTTTAATTAAAAAATTCTACAATAAAAT
TATTTTTTAACATTACAGTAATTAAAATTTACAAAGAAACGATAAAAGTTTAATTAAAAAATTCTACAATAAAAT
***************************************************************************

TATATCAAACAAGAATAACTAATAACTAAAAATTCGAAAACCAATCTAAATATAATCCAAAAATGATTCAAGAAT
TATATCAAACAAGAATAACTAATAACTAAAAATTCGAAAACCAATCTAAATATAATCCAAAAATGATTCAAGAAT
***************************************************************************

AATTGAAAAATTAGAGAATAATAATCAAAAAATAATAATAATTTACAAAAAAACGATGAAAGCTTAATGAAAAAA
AATTGAAAAATTAGAGAATAATAATCAAAAAATAATAATAATTTACAAAAAAACAATGAAAGCTTAATGAAAAAA
***************************************************************************

CTCTACAATACAATTATATCAAACAAGAATAACTAATAACTAAAAATTCGAAAACCAATCTAAATATAATTCAAA
CTCTATAATACAATTATAACAAACAAAAATATCTAACAA
***** ************ ******* **** **** **

AATGATTCAAGAATAATTGAAAAATTAGAGAATAATAATCTAAAAATAATAATAATTTACAAAGAAACGATGAAA
AATGATTCACGAATAATTGAGAAAATTGAGAGTAATAATCTAAAAATAATAATTATTTACAAAGAAACGGGAAAA
********* ********** *** * **** ********************* ***************   ***

GCTTAATGAAAAAACTCTACAATAAAATTATATCAAACAAGAATAACTAATAATTAAAAATTCGAAAACCAATCT
GCTTAATGGAAAAACTCTACAATTAAATTATATCAAACAAGAATAACTAATAATTAAAAATTCGAAAACCAATCT
******** ************** ***************************************************

AAATATAATCCAAAAATGATTCAAGAATAATTGAAAAATTAGAGAATAATAATCTAAAAATAATAATATTCCACC
AAATATAATCCAAAAATGATTCAAGAATAATTGAAAAATTAGAGAATAATAATCTAAAAATAATAATATTCCACC
***************************************************************************

CAGAATTTTCTCTTATTCCTCGAACGATCCACTTTAAAGTAATCGTGCAAATCAACCGACAGATTACCTCGTTCA
CAGAATTTTCTCTTATTCCTCGAACGATCCATTTTAAAGTAATCGTGCAAATCAACCGACAGATTACCTCGTTCA
***************************************************************************

CTTCATTATCAATTAACTTTTATTTACGTCCTTGCGATCGTATTATATTAATACCGTAATTGAGTATAATAAGTC
```

Commented [AB2]: Primer F_Ex2 & R_Ex2

Commented [AB3]: Sequenzierung 18.12.25
Seq. ID KPV891
Exon2-4 For2

CTTCATTATCAATTAACTTTTATTTACGTCCAAGCGATCGTATTATATTAATACCGTAATTGAGTATAATAAGTC
****

GCCGTGAATGCGTGAATCACACGCATTCAACGGATGCATTCGTTCGGCGTTCGCTCTGTTTGCTCTGACTCACTG
GCCGTGAATGCGTGAATCACACGCATTCAACGGATGCATTCGTTCGGCGTTCGCTCTGTTTCGTCTGACTCACTG
****

ACTAAACCTCGTGACAACTATTAAATTTGCTAAAATAATGGAAGTAGTAATGAAATAGTTGCGAATGGCGGCGCG
AATAAACCTCGTGACAACTATTAAATTTGCTAAAATAATGGAAGTAGTAATGAAATAGTTGCGAATGGCGGCGCG
* **

TTTGTATGCTTGATATCAGAGTCCAATTCCAAAAGTCGTTTCCTATACGTCAATAGACCGCGAGCGTCATACCTG
TTTGTATGCTTGATATCAGAGTCCAATTCCAAAAGTCGTTTCCTATACGTCAATAGACCGCGAGCGTCATACCTG
****

TTTCGCACGAATAATTATCATTCAACCGAGCGAGTTGAATCGAGAAAAAAAAAATGATTCTGATTATCAGTCAGTC
TTTCGCACGAATAATTATCATTCAACCGAGCGAGTTGAATCGAGAAAAAAAAAATGATTCTGATTATCAGTCAGTC
****

AGGGGGTAATTAGGATCTGAGATAAAAAAAAAAAAGACGGCGCAAAAGAATGACGATCGAAATAATGGAT**CGAACT**
AGGGGGTAATTAGGATCTGAGATAAAAAAAAAAAAGACGGCGCAAAAGAATGACGATCG
****

TTGGTTAGAAATGACCGGTTCATAGAGATAGAGAAAAGAATTAAAATTATATAAAATTTGTTAGGTGAGAAAGAA
TGAAGATGAATGAAATGTTGATAAATGAAGAATTTTTTTATTTCATTGAAAAATAGAATGTGCAAAAAGTTTAAC
AAAAAGTAGAGATATGAGAGAATCTTTGTTCGAGATGTTGTAATACTTTTCTAATTAAAATAACCGGGAAAAAAT
AAAAATAAAATAAAAATATTGAAAAATTTTAATTCCTTACGAGATATTTTCTTGTTTCGAAAAATATAATTATTT
ATAATCTAATATTAAATGATATAATGTTATTTAATTTTAATAATATTATTAAATAAGAATGAGATGTTAAATGAA
ACTTACTTATGAGAGAAAAATGAAGTTGATTATGCTTGTTTGATTAGAATTATTTGTCTAAGCACATCTATGATA
TTTATTGATAATCGTAAATAACTCCAAGTTAGTCTATTTTAAAAGTAATAAAATTTAACTTTATATTGTTTTTTA
TTTAAAAAAAATAAAAGAAAAAAAATAGAAATAGTTAAGAAACTGTAAATATAAATTGCAATGCATAATGAATGC
GAGATATCACATTATTACGTCGTTGAAGTTCCATATCTTAATGAGGTGATAAACTGTCTTCAAAGAAATATATTA
CGTAGGGTTAACATTGAAATTATGATGATTTTGCGAAGAGATACTACATCAGCTGTTAATCTACGTACGTAATTT
TTGAACAAATAAACCAGTCTTTAAAAAAGACAAATATTAAAAAAATTAATTTGACCCTGCACCAAGTAATAAATA
ATTTAAAAAGAAAACTACACGATATATAAAAAAAAAACAAAATTTTTAACTTCATTTAAATCACATTTATATTAA
AACTAATATAATTATCGTTTATTACACCAAAGAAGAAGAGGCAAAAATAATAGAAATCTCCAAAGCATCTCTCTA
TTTTATCAAAATAATAGAAGCCAATTTTAAGGGAGCAACAAAGCAAATTACAGCTCTATTCTTTCAACACATCTC
TATCCTTTTATATCCAAGAAAACTGTAGAAAAAAATATACACTTGCACCTTCACGTACTTGTCTGACCGGCTCCG
AATAAAGACCGGATTTTTAGCGTTTCTAGACCTTGAAAGAAACTGGACGCCAGTTAAACCGTTCGTTCGCATTGA
AAACACGCTCCATTCCATGGACAAAAATCGATGGTAGAAAAGAAGGGAAAAGAAAAAGAAGAGATAACAACAGTT
TTTGGAAAAATGGAGGTACATGAATGTCAAACAATATCTTGAAACGACGAGAACTGTCGTTTAACGAGAACTGGC
TATTAAAACAAACCTTGAGAGAAACGTCGAGTCGACAACGTGACGAACGAAAAAACAAATTGTTTCCGGTAAAAT
TTCAATGATTAGTTTGGGTAAAAACGAGAGTGCGTCGTTGATAAGAGAGATTTCTAATAACTGAGAGACTGGAAT
ATCAGATATTTTTGAAATATCAGATATTTTTGAAATATCAGATATTGATTTAATTCCATCGATAAATTTAAGAAA
TTGTTTTTGTAACAGCTTGGATTTGAATATTTTATTTGGAATGAAAAATAAGAATCGAGAAGACTTCTAAAAAAG
AATTGTATTGTAATATAGCTAATTTGAAAAACATATAAAAATTGAAGAGATTGAATAAATGAATGGAGTATAAAT
AATTTTTGCAAAAAATAAAATAGGAATTTGTTTCATAAAGAATCGCGTAGGAACCGGAAATAGCTTATGTTTGAA
AATGAAGACAATGAAGTCTCAATGATGATGCTGAGAAATGACTCATTGAGTTCGATGAAATGCAGCAGATTCATC
AATGGCGCTTAAAAAAATGACTCATACGATATACAGTAACTTGATCACGCGAACATTTTCAAACATTTTCCTTAT
TTTAATTACGATCTGAAATTATTAATCGTTGATTTATTTTTAAATAATTTTAATGTTCCGAAATTAAATTTTTTA
TGAATGTTTATTTTTTAATAATAATAATGAAAACAATAATGTAACAATCAATAAATTATATTGAATCAAAAATTT
CGATTTAACAAATATGATTATTGTATCATATTATATAATATTTAATATTATTCTATGATCTACTTAATATTGAAA
CTTGATTTTTGTTTCATTTAATTGAAATTAAAATAATAATATCAAATACAATTATTACAATCGATTTTAAAAAAA
TTTCCTAATCATCTAATAGACATCTTATTAAACATTAATTATTAAACATCGATCATTCTTTTTTTTATCGACTTA
ATAAATTATATGTAATATTTCAAAGTGATTAGATAGACATTATTAATATTCTGAAATTAAAAGCAATTAAAAACA
CGCATCGAATAACAAAGAATAATAAATATTAAAAAATATATTGCAACAAGAAGAGATAATGATAAAAATTGTAAC
GCAATTACGTTTTTCTATTGATTTTCTACCAAAAAAGACAAGTTGCCCAAGAAAGATTTCTATTTCGAATAATGA
AATCTCCAACCGGAACGAGTAATCTGTGACACAAAAATTATCGAATTTGTCGATTATTATGAAATCGTGGTAAAA
GTCATCTCGTATTTCCTGTAGAGAAATGATTTCACTTCGATCATTCGTAATAACGTCTGTACTCAGTTTGAAACG
CGCACAAATAAAAAATAAAATAAATCACGTATGAACCAGAAAATTTTCCATTTTTGAAATGTATAAATAATAAAA
TAAAGATTGGAGAGCGAAAGAAAAAATTAAATTACAATTTAATTGAAATAACTTAGATGATTAGAAAATTATGAC
TTCAAAGTATTATTGAAAGATCATAATTAAAAATTCAATTTTTGAAAATAATACTGGATTGATGGATACGGATAA
AAAAACGAAGGTGAACGGAAGAATGATCTAACTATTAAATTGCACCCTATATAGCGGTGTTTATTCGAGATGAAA
CGGTATTTATCGACAGCAATCATTTGCCACTATCAAACTAGAATTTCTTCTACTATGTGTATACTTAACACAATC
AACCTTGTACTTATATTATTGAACGATTATGAATTAACGTAACAAATATGCTAGAAAGAGGAAGAGGATATTTCG
TAAGAAGTTGTTCAACAAAAGTGACAAATTTCATGAATTCCCGTGAAATCGATTATTACGCAATCGTGTGTTTTC
GAATATTTGCAAGAAATCTCTTCATTAATGGTTAAAAAAAAAAAAACATCGTGAAAAAAAAAACAGATGTTAACCG

Commented [AB4]: Primer R_In2

Commented [AB5]: Sequenzierung 22.01.26
Seq. ID KPV897
Exon2-4 RevIn2

```
ACAATACTTTATATATTATTTATATTTTGAATTACGTATACATAAGAGAATTATGAATTGATTTTAATTATTTCC
ATTGACCTGATTCATTAAATTAATTTATAAAGAACGAAAATAGCTTGAAAATGATTTGATCATTGCTATTCCTAT
GATCTGTCATTTTATAATTAAAACTGAATTATCATATTACATTGTCAAGAAGAAAGAAAAGTAATAATGAAGCAC
TAGTTACGTGTTCAGAATGATAAATATTGTAATTAGTATACATATGTGTCAATATGTAAAATGTAAAATAACCGT
ATTCTAGTCCTTCTAGTATTATAATTATAGCTATCATAAATGGTCGTCATATCGACTACTTTTCTAACTACGCTA
TTGTTCTGTCAGTAAATTTCAATATGATACAATAATGTATTCTACTTTTTATTTTTCTATTGCAAACATTAGTTT
TTGAATGAAAAATATTGATATTTCTGTGACAAAAAGTTATCTATTAAAATTTCATCGTCAAAAACTAATTATTAG
ATAAAAATCAAAATATGTTTCAAAATATCTACGATTGTAGATAATAAAAGAGAAAAAAATACGTAATGAATGTTA
CAACAAAGAAAAAAATAATGTTATATAAATCATTAAGCTGAATCAGATGTGATAACACTAATAATCAACGATAAA
ACGGAAAATAATAATACATTTAATAATATATTCATTTATCATGTAGTAAATACAATGAAAAGAATAAGTTAATTT
CAAGCTGAAACTTCGAACCGATGAAAGAAAAATATTTCATCAACTGCATTAATGATTATTATATAATTTGTTTTG
AAATTAAAATTTTTTCATCACAAAGTAAAGAATCACATTTGCAATTATATAACTATTGCTGACTTCAGTTTTATA
AAAATATGGAATTCTTGCCAATTGAATCAATTTGATCAAATTTGCATTGAAAGAAAAATAAGTCGAAGTCCACGT
GTTATCCACTTCGAAAAAAGTAAATATAATTGCTCTTCTAACTTCATGAAGGATACTTAACATTTACACGTGTGT
AAATTTAATTTTTTCTTTTTAATTGAATGAAAACTATTGCGAGTAAATATTTTTGTGCATACAAATCTTTTCATG
AATTGTTAATTAAATATTTTTTAAAAAAAGAAAAATTAGATATCGAATTTATTAGCTATTCAGAGAAGAAAATAA
TAAATTGAGGCCTCTTTCTAAGTTATAAAATTTTTCATAAAAAAAAAGTATGCTAAACTCGATATATATCTCATA
AATAACTTAAATCTATATGATAAATTATAAAAATCTATTTAATAAATTTATGGATTAAAAAATATATTCTAAATC
TATATTAAGAAAACCAGAATATGTAGAATATTATTTGAAATTCAAAACCTAGTAGAAAACTGACATTTATCATGC
AATAATTTAACAGAGATATTTCCAGTACATTTTCAATCTATTCTAAACACACTCACACCAATATCGAACAACTCG
                                                       CACCAA-ATCGAACAACTCG
                                                       ****** *************

ATGGCTCGTAACCATATTCAAATCGATTTACACGATCGTTCCGTTAATACATCACAATTTCCACGTAAAGAAGTA
ATGGCTCGTAACCATATTCAAATCGATTTACACGATCGTTCCGTTAATACATCACAATTTCCACGTAAAGAAGTA
***************************************************************************

TTAATAATAATTCGAATCGCGTGACAAGTAAAACTCGGATCAAACGTTTTCAAACCGTTTCGAGTAAAATATTCA
TTAATAATAATTCGAATCGCGTGACAAGTAAAACTCGGATCAAACGTTTTCAAACCGTTTCGAGTAAAATATTCA
***************************************************************************

GAGATTAAAACACGATCGAGATCGAGATAATTATACCGATATATAATTCTTAAACCGTGATCTTTAGAAATCTAA
GAGATTAAAACACGATCGAGATCGAGATAATTATACCGATATATAATTCTTAAACCGTGATCTTTAGAAATCTAA
***************************************************************************

AAGTCTAAACAAAGATCTATTGATTGATTTTTCAAACTAAAATTTAAATTTACTCGAATTCAATACAATTTTATT
AAGTCTAAACAAAGATCTATTGATTGATTTTTCAAACTAAAATTTAAATTTACTCGAATTCAATACAATTTTATT
***************************************************************************

AATAGATTATAATCGTGTTATCGATCATCTTAAGATTGGTCTTAAAATGTACGAATAATTTATCACGTACTATGC
AATAGATTATAATCGTGTTATCGATCATCTTAAGATTGGTCTTAAAATGTACGAATAATTTATCACGTACTATGC
***************************************************************************

ATTATTATTCATGTACGTGTTCACTTGATGCATATTGCACCGCGATACAAGGAAACGCACGTATGAATGATTGTA
ATTATTATTCATGTACGTGTTCACTTGATGCATATTGCACCGCGATACAAGGAAACGCACGTATGAATGATTGTA
***************************************************************************

AATAAAGAACGTAGAACCATGTAATATGGTTTTAAGGATCGATAACACTTTACGAATCCTATATAATGAATAAAC
AATAAAGAACGTAGAACCATGTAATATGGTTTTAAGGATCGATAACACTTTACGAATCCTATATAATGAATAAAC
***************************************************************************

TGAATTTCATAAGAAACGAATTATGAAAGAAATGTATTCGATGCGTTTTAAAAAAGAATTGCTTTCCATTAAGTT
TGAATTTCATAAGAAACGAATTATGAAAGAAATGTATTCGATGCGTTTTAAAAAAGAATTGCTTTCCATTAAGTT
***************************************************************************

AATAAGAAAATGAAAATTATGTATTGAAATGAACATACTAGAATAATAAAATCGATTAAATTTTAATGGAAGAAT
AATAAGAAAATGAAAATTATGTATTGAAATGAACATACTAGAATAATAAAATCGATTAAATTTTAATGGAAGAAT
***************************************************************************

ATTATCTGATGTGTTATGAATGGATGAAAATTAATAAGATATTATATTGTGGATAAATAATTTTCTTTTTAAATT
ATTATCTGATGTGTTATGAATGGATGAAAATTAATAAGATATTATATTGTGGATAAATAATTTTCTTTTTAAATT
***************************************************************************

AAAAAAAATATAATTTAATTTTATCTTAAAAATTAAATTATAAGCAATGTTAAAAATTTATTATGTTATATTTGT
AAAAAAAATATAATTTAATTTTATCTTAAAAATTAAATTATAAGCAATGTTAAAAATTTATTATGTTATATTTGT
***************************************************************************

AATTATTATTGAAAAAATTGTTTTTTCCTTTTTTTTTAGGTGATATCGATGAAGAAACATATAAATTAATGACATT
AATTATTATTGAAAAAATTGTTTTTTCCTTTTTTTTTAGGTGATATCGATGAAGAAACATATAAATTAATGACATT
***************************************************************************

GCCAAGGTGTGGTGTTAAAGACAAAGTTGGTCCTGGCTTTGGAAGATCGAAGCGATACGCTCTTCAAGGTAAATC
GCCAAGGTGTGGTGTTAAAGACAAAGTTGGTCCTGGCTTTGGAAGATCGAAGCGATACGCTCTTCAAGGTAAATC
***************************************************************************
```

Commented [AB6]: Sequenzierung 22.01.26
Seq. ID KPV896
Exon2-4 RevIn3

Commented [AB7]: Primer F_Ex3

```
AAATTATAATTTAATTCCATAAAATGGAAAAATTTAATTCTATGAATATCTTTTCATGCAATGCAAATATTTTCA
AAATTATAATTTAATTCCATAAAATGGAAAAATTTAATTCTATGAATATCTTT-CATGC
 AATTATAATTTAATTCCATAAAATGGAAAAATTTAATTCTATGAATATCTTTTCATGCAATGCAAATATTTTCA
 **************************************************************************
CTCGCGTATCAATTTCAATTGTTAAAAATATCCAAATTGAAAAATAAAAAAAATAGAAAAGTAAAAAAAAATTTCC
CTCGCGTATCAATTTCAATTGTTAAAAATATCCAAATTGAAAAATAAAAAAAATAGAAAAGTAAAAAAAAATTTCC
***************************************************************************
TTTTACAAAGAAAAGAAAAATAGATTATAAAATAATTATGAATTTAATCATTCTAATCTAATCAAACTTCATCAA
TTTTACAAAGAAAAGAAAAATAGATTATAAAATAATTATGAATTTAATCATTCTAATCTAATCAAACTTCATCAA
***************************************************************************
AATATCTTATGACATAACAATCGAACTATCTAATCGAATCATGTAATAAAATCCAAAATCTAAATTCTAATCACA
AATATCTTATGACATAACAATCGAACTATCTAATCGAATCATGTAATAAAATCCAAAATCTAAATTCTAATCACA
***************************************************************************
TTTGACTTCCATTCTCATTAAAATATAATGCTATTTATCGTTAATATTTTCTACGATAATAATATATATCATGTA
TTTGACTTCCATTCTCATTAAAATATAATGCTATTTATCGTTAATATTTTCTACGATAATAATATATATCATGTA
***************************************************************************
CATCAATTTAATGATCATATATTCTAAAATGAATTTATTTAAGAAAAATTTATCAAAGATACTAGAATTCGGAGC
CATCAATTTAATGATCATATATTCTAAAATGAATTTATTTAAGAAAAATTTATCAAAGATACTAGAATTCGGAGC
***************************************************************************
AATTACAAGAATTAGAGAATATTAAAACCAATAATTTAAATAATATAATAAAAATGTTTAATAATTAATAGAGAA
AATTACAAGAATTAGAGAATATTAAAACCAATAATTTAAATAATATAATAAAAATGTTTAATAATTAATAGAGAA
***************************************************************************
AATTTGAATTTAAAGTTATTACACAAAATAACTGAAACAATTATTAAAAATTCGTGTAATTATCCGAGTAGTGGA
AATTTGAATTTAAAGTTATTACACAAAATAACTGAAACAATTATTAAAAATTCGTGTAATTATCCGAGTAGTGGA
***************************************************************************
ACGCATTCACAATTCAATGTTACTTCTAACTTCCTGTCACATTATCCTTATTATAGAATCAGTCACCTCAAACGG
ACGCATTCACAATTCAATGTTACTTCTAACTTCCTGTCACATTATCCTTATTATAGAATCAGTCACCTCAAACGG
***************************************************************************
AATATATACACACCTGTAGCCTTTTGTTGAATGCTCTAAATGCATTTTTTATGCATCAAAATTGTTCCAAATACA
AATATATACACACCTGTAGCCTTTTGTTGAATGCTCTAAATGCATTTTTTATGCATCAAAATTGTTCCAAATACA
***************************************************************************
ATTCTGAGAAGGTTTTTTATGCGACTTATTAAATGAATCTTTGTTTTCCAATTTATAATTAAAATTTCGATACAA
ATTCTGAGAAGGTTTTTTATGCGACTTATTAAATGAATCTTTGTTTTCCAATTTATAATTAAAATTTCGATACAA
***************************************************************************
TTGACCGAATATCAGATGTAAAATTTAATTTTTTAATAAAGTTAATTTGACAACTTACATTTTCCTCAAAACTAA
TTGACCGAATATCAGATGTAAAATTTAATTTTTTAATAAAGTTAATTTGACAACTTACATTTTCCTCAAAACTAA
***************************************************************************
CATATAAATCCTCACAAGAAAAAAAAAAATTTTTATTAAAAAAAAATATTTTAAAAAATCTATGTAAAAATAATATA
CATATAAATCCTCACAAGAAAAAAAAAAATTTTTATTAAAAAAAAATATTTTAAAAAATCTATGTAAAAATAATATA
***************************************************************************
TGTCCAATCATTCTATCGTCGATTCTAACAATTTAAAATTCCACAAAATCATGATTCCAGTTATCCAATCAACTA
TGTCCAATCATTCTATCGTCGATTCTAACAATTTAAAATTCCACAAAATCATGATTCCAGTTATCCAATCAACTA
***************************************************************************
TAACCCATAAAACACTTCGTTTCCTTTTCATTAACTCTTTCTTCTCGCAAGAAAATGATTTTTTGATTCTAACTT
TAACCCATAAAACACTTCGTTTCCTTTTCATTAACTCTTTCTTCTCGCAAGAAAATGATTTTTTGATTCTAACTT
                                                    AAATGATTTTTTGATTCTAACTT
***************************************************************************
ATTCAATCGCACGAAAAGGATACAATCTATCTAAAAATTATCGAGATTCTTCTAACCATGACTTTGAACCTCGTG
ATTCAATCGCACGAAAAGGATACA-TCTATCTAAAAATTATCGAGATTCTTCTAACCATGACTTTGAACCTCGTG
ATTCAATCGCACGAAAAGGATACAATCTATCTAAAAATTATCGAGATTCTTCTAACCATGACTTTGAACCTCGTG
************************ **************************************************

CAAACGTGCCTCGTTTCAGTGAACTCGTGGCGACAAGATCACCGATGCGTGTTTCCTATGGATTTTTATCGCGTA
CAAACGTGCCTCGTTTCAGTGAACTC
CAAACGTGCCTCGTTTCAGTGAACTCGTGGCGACAAGATCACCGATGCGTGTTTCCTATGGATTTTTATCGCGTA
***************************************************************************
ACAGATCCCCTGGATGACCCATTAGATCGATCATGATCGAAACGACGATAGAAATTTATCAGGGCTGAAAATTGG
ACAGATCCCCTGGATGACCCATTAGATCGATCATGATCGAAACGACGATAGAAATTTATCAGGGCTGAAAATTGG
***************************************************************************
ACCGTTTCAAAGCTAGATTGTGAAGAGTTTATTGTTCGAAGAAATTTGTTTTGACAATCGAATAAATTAGAGATC
ACCGTTTCAAAGCTAGATTGTGAAGAGTTTATTGTTCGAAGAAATTTGTTTTGACAATCGAATAAATTAGAGATC
```

Commented [AB8]: Sequenzierung 04.12.25
Seq. ID KPV876
Exon3-9 For3

Commented [AB9]: Primer: R_In3

Commented [AB10]: Primer F_In3

Commented [AB11]: Sequenzierung 11.12.25
Seq. ID KPV880
Exon3-9 For_In3

```
***************************************************************************
AAAAGTGGAGGAGAGATTTGAGATAAATTAAAATAGAAGAATGAAATATTCTATTCTATTCTATTCTATTCTAAT
AAAAGTGGAGGAGAGATTTGAGATAAATTAAAATAGAAGAATGAAATATTCTATTCTATTCTATTCTATTCTAAT
***************************************************************************
ATTTTGTTTGTTTGTTTTTCTTTTTT-CTTTTAATATCTAAAATTTTCTCTACTCTTAGTCTATTCATCTATTCA
ATTTTGTTTGTTTGTTTTTCTTTTTTTCTTTTAATATCTAAAATTTTCTCTACTCTTAGTCTATTCATCTATTCA
************************** ************************************************
AAGAAGGATATAAGCTTGGACGAGTTTCGCTCTTTAATGATGGAGGTATAATTGATCGACCTTGCTGTTAGATGT
AAGAAGGATATAAGCTTGGACGAGTTTCGCTCTTTAATGATGGAGGTATAATTGATCGACCTTGCTGTTAGATGT
***************************************************************************
GATGTTTGACAAAGATAATAGATCGGTGATGCGATGACAAGGCTACGTATTTGACAATGAGCTTCTTTGTAATTT
GATGTTTGACAAAGATAATAGATCGGTGATGCGATGACAAGGCTACGTATTTGACAATGAGCTTCTTTGTAATTT
***************************************************************************
ATGGATTACTTTTTTTTTTATCGTTGCTTCATCACAATTCAAAAATATTTGAAATATTGCAAAGATTTTTTTAGA
ATGGATTACTTTTTTTTTTATCGTTGCTTCATCACAATTCAAAAATATTTGAAATATTGCAAAGATTTTTTTAGA
***************************************************************************
TATAATGGCATATATTGATTAAATTTATCAATAATTTTTGAAATTAAAAAAAGAATTTAATTCAATGATTCCTAG
TATAATGGCATATATTGATTAAATTTATCAATAATTTTTGAAATTAAAAAAAGAATTTAATTCAATGATTCCTAG
                                              AAAAAAGAATTTAATTCAATGATTCCTAG
***************************************************************************
AAGATTGCTTGTCTTTTTTTATAATAATAGATTGTTTTGTTAAATACTTTTAACGATCGAGACAATATTATTTCT
AAGATTGCTTGTCTTTTTTTATAATAATAGATTGTTTTGTTAAATACTTTTAACGATCGAGACAATATTATTTCT
AAGATTGCTTGTCTTTTTTTATAATAATAGATTGTTTTGTTAAATACTTTTAACGATCGAGACAATATTATTTCT
***************************************************************************
ACAATTTTTCAAAATGATAACTCACCAACACATTTGTGATGAGAGTTATATGTATAATACATTTCACGATTGTTT
ACAATTTTTCAAAATGATAACTCACCAACACATTTGTGATGAGAGTTATATGTATAATACATTTCACGATTGTTT
ACAATTTTTCAAAATGATAACTCACCAACACATTTGTGATGAGAGTTATATGTATAATACATTTCACGATTGTTT
***************************************************************************
CTAATAGCAAATAAGATAAGAACATAAAGAAGATCTATGGAAGAACTTAGATATATACAAGTACAAAGTTCGTCG
CTAATAGCAAATAAGATAAGAACTTAAAGAAGATCTATGGAAGAACTTAGATATATACAAGTACAAAGTTCGTCG
CTAATAGCAAATAAGATAAGAACATAAAGAAGATCTATGGAAGAACTTAGATATATACAAGTACAAAGTTCGTCG
*********************** ***************************************************
ATGCGTGTTTTAGACTTTTCAGACACGTTGTCGTGATTAAGGGGTGAATCAATACACTTGATTAGAAAAATGGAT
ATGCGTGTTTTAGACTTTTCAGACACGTTGTCGTGATTAAGGGGTGAATCAATACACTTGATTAGAAAAATGGAT
ATGCGTGTTTTAGACTTTTCAGACACGTTGTCGTGATTAAGGGGTGAATCAATACACTTGATTAGAAAAATGGAT
***************************************************************************
TACTAATAATGGAAAATCACGTCCCGATAATTCTTTCCAGATAGATGAAAATCAAATGAAACGTTTCGTTATAAA
TACTAATAATGGAAAATCACGTCCCGATAATTCTTTCCAGAT
TACTAATAATGGAAAATCACGTCCCGATAATTCTTTCCAGATAGATGAAAATCAAATGAAACGTTTCGTTATAAA
***************************************************************************
CTTCATCGCTGAAGTCTTATTCAAAATGAACGTTTATTTAAAAATAAATCGTTGCGCAAGAGACAAATAAAATGG
CTTCATCGCTGAAGTCTTATTCAAAATGAACGTTTATTTAAAAATAAATCGTTGCGCAAGAGACAAATAAAATGG
***************************************************************************
TAGTGAAATGATTGGCACATAGAGTACCGTTATATAAAAAAACATTATAGAGCTGCCAAGATCTTCTTTGTAAAT
TAGTGAAATGATTGGCACATAGAGTACCGTTATATAAAAAAACATTATAGAGCTGCCAAGATCTTCTTTGTAAAT
***************************************************************************
ATATATTAAATAACGATCGAAGATCCCTATGATATATATTTCTTCTACCAAATTAATTTTTCAAAAGTTTATCAA
ATATATTAAATAACGATCGAAGATCCCTATGATATATATTTCTTCTACCAAATTAATTTTTCAAAAGTTTATCAA
***************************************************************************
ATTTTTATTATTTTCAATTATATAGATATCCTAAGAAAACTTGTTACACGCCCAATCATTCACTCCTCTTTTTCT
ATTTTTATTATTTTCAATTATATAGATATCCTAAGAAAACTTGTTACACGCCCAATCATTCACTCCTCTTTTTCT
***************************************************************************
ATTATTAACTATAATTTATTAACTATAATTTTTTATTAACTATTTTTTTATTAACTATAATTTTATAGTTTTAGA
ATTATTAACTATAATTTATTAACTATAATTTTTTATTAACTATTTTTTTATTAACTATAATTTTATAGTTTTAGA
***************************************************************************
AATTTAATTTATTCTTGAAATTCAAAACAAATTTCAGATATTTTGTTATCTGAAATTTGATTTATCTTTTGATTG
AATTTAATTTATTCTTGAAATTCAAAACAAATTTCAGATATTTTGTTATCTGAAATTTGATTTATCTTTTGATTG
***************************************************************************
ATCTAATACATTGTTATCAAAATATATATTTTGCATATACAATTTTCATAATCATAAATCGGAATGTTTAATGTA
ATCTAATACATTGTTATCAAAATATATATTTTGCATATACAATTTTCATAATCATAAATCGGAATGTTTAATGTA
***************************************************************************
```

Commented [AB12]: Sequenzierung 11.12.25
Seq. ID KPV881
Exon3-9 Rev_4

ATAGTTGATAGAAGAATCGAAAGAAAAAGAAAAGAAATATTTTATTACAGGCTCTAGATGGCGTGTGAAGAAATT
ATAGTTGATAGAAGAATCGAAAGAAAAAGAAAAGAAATATTTTATTACAGGCTCTAGATGGCGTGTGAAGAAATT
****

GTCGTACAAGATCAGCAAGTATCCGCGAAATTTGCCACAACATAAAGTTGATGCCGAGTTGAACAAAGCGTTCAA
AATTTGCCACAACATAAAGTTGATGCCGAGTTGAACAAAGCGTTCAA
GTCGTACAAGATCAGCAAGTATCCGCGAAATTTGCCACAACATAAAGTTGATGCCGAGTTGAACAAAGCGTTCAA
****

AGTATGGTCGGAGTACACGGATCTCGTTTTCGTCCAAAAGAAGTCAGGCCAGGTGAGACGAATAATATTTACCGA
AGTATGGTCGGAGTACACGGATCTCGTTTTCGTCCAAAAGAAGTCAGGCCAGGTGAGACGAATAATATTTACCGA
AGTAT
****

GATATTTCACAATCATGAAGTGAAAAAGAAATGAAAAGCAAAGAATAGTGATCTTATAGCTTATAGCTAAAAATC
GATATTTCACAATCATGAAGTGAAAAAGAAATGAAAAGCAAAGAATAGTGATCTTATAGCTTATAGCTAAAAATC
****

TGGTAATGCAAACATTGTTACCAATGATGATTTCGCATGATTTTATCATTGCTTATTTTTCATGAAACCATCAGC
TGGTAATGCAAACATTGTTACCAATGATGATTTCGCATGATTTTATCATTGCTTATTTTTCATGAAACCATCAGC
****

GATATTAATCGCAATACTTTAACGTCTATGAGATTTATATTAAATAATTATACAATTATAATTGAAAAATAAATA
GATATTAATCGCAATACTTTAACGTCTATGAGATTTATATTAAATAATTATACAATTATAATTGAAAAATAAATA
****

TTCAATAGAATATTCGAGTAAAATTCAACAAAATTGTTACAATCCATAAATAATATTAGATATAATAAAGATATA
TTCAATAGAATATTCGAGTAAAATTCAACAAAATTGTTACAATCCATAAATAATATTAGATATAATAAAGATATA
****

TTTATAATTTGGATTGAGTTATTCTAAAAGAAAAAAGCTAATCAAACTAATATTTTAATAAGACACATAAAAAAA
TTTATAATTTGGATTGAGTTATTCTAAAAGAAAAAAGCTAATCAAACTAATATTTTAATAAGACACATAAAAAAA
****

TGTCTGAAATATCTGAAAACATTTCGATAAAATTCTTATAGATCCTTCGAATAATTTTAACTTTTTAGATTGTAA
TGTCTGAAATATCTGAAAACATTTCGATAAAATTCTTATAGATCCTTCGAATAATTTTAACTTTTTAGATTGTAA
****

CAATTTTCAATCCACTTTTGTTAGGTCCATATCGAAATTAGATTCGAGAAAGGCGAACACGGGGACGGAGATCCT
CAATTTTCAATCCACTTTTGTTAGGTCCATATCGAAATTAGATTCGAGAAAGGCGAACACGGGGACGGAGATCCT
****

TTCGATGGACCTGGTGGCACTTTGGCTCACGCTTACTTCCCCGTATACGGTGGTGATGCTCACTTCGACGACGCA
TTCGATGGACCTGGTGGCACTTTGGCTCACGCTTACTTCCCCGTATACGGTGGTGATGCTCACTTCGACGACGCA
****

GAACAATGGACCATCGACAGTTTCCGTGGAACGAATCTGTTTCAAGTAGCTGCTCACGAGTTTGGCCATTCTCTC
GAACAATGGACCATCGACAGTTTCCGTGGAACGAATCTGTTTCAAGTAGCTGCTCACGAGTTTGGCCATTCTCTC
****

GGTCTGAGCCACAGCGACGTTAAATCCGCTTTAATGGCACCCTTTTACCGCGGCTATGAGCCTTATTTCCGGTTA
GGTCTGAGCCACAGCGACGTTAAATCCGCTTTAATGGCACCCTTTTACCGCGGCTATGAGCCTTATTTCCGGTTA
****

GACGAGGACGATATTCAGGGTATCCAGGTAAATACAAAGTTCAAAATGTTGGGAGAATTAATTTTTCATAAACGA
GACGAGGACGATATTCAGGGTATCCAGGTAAATACAAAGTTCAAAATGTTGGGAGAATTAATTTTTCATAAACGA
****

AGAATGACAGAATTTCTTCGATTTGAGAAAAAAATTGGAAGAATACATTTTGAAATGGAATGGAAATTGATTGAG
AGAATGACAGAATTTCTTCGATTTGAGAAAAAAATTGGAAGAATACATTTTGAAATGGAATGGAAATTGATTGAG
****

AAGAAATATACGAATTGTATAGCACGATGATGAGGCAGAAGAGTTTAAATTTGAACTTTTCGCGTGATTTAGGCT
AAGAAATATACGAATTGTATAGCACGATGATGAGGCAGAAGAGTTTAAATTTGAACTTTTCGCGTGATTTAGGCT
****

CTGTATGGAACGAAAACGTCAAATAACGGTGGAGTTCCGAGCGGGCCAAAACCAACTACCACTGCATCGCCTAGC
CTGTATGGAACGAAAACGTCAAATAACGGTGGAGTTCCGAGCGGGCCAAAACCAACTACCACTGCATCGCCTAGC
CTGCATCGCCTAGC
****

GAAGAGGACTCGGAACTGTGCACGAATTCAAAGGTCGATACCATGTTCAATTCTGCGGAGGGACATATGTACGTG
GAAGAGGACTCGGAACTGTGCACGAATTCAAAGGTCGATACCATGTTCAATTCTGCGGAGGGA
GAAGAGGACTCGGAACTGTGCACGAATTCAAAGGTCGATACCATGTTCAATTCTGCGGAGGGACATATGTACGTG
****

TTCAAGGGTACGTATATGTCAGAATTGGTATGATAACGAAATATCTTTATATATATACTATTAATATATTTATAA
TTCAAGGGTACGTATATGTCAGAATTGGTATGATAACGAAATATCTTTATATATATACTATTAATATATTTATAA
****

Commented [AB13]: Primer F_Ex4

Commented [AB14]: Sequenzierung 04.12.25
Seq. ID KPV878
Exon4-10 For_4

Commented [AB15]: Primer R_Ex4

Commented [AB16]: Primer F_Ex6

Commented [AB17]: Sequenzierung 11.12.25
Seq. ID KPV882
Exon3-9 For_6

```
AATAGAATTATATTTATCTAAATTTTATTTTATTCATTGAACTGATCTATCCTCAACTTTCCCTTACTATATAGA
AATAGAATTATATTTATCTAAATTTTATTTTATTCATTGAACTGATCTATCCTCAACTTTCCCTTACTATATAGA
           TTTATCTAAA-TTTATTTTATTCAT-GAACTGATCTATCCTCAACTTTCCCTTACTA-ATAGA
           ********** ************** ******************************* *****
AATATTATTTACAAAATAATGGGAAAAATTAAATTTTGTACGAATTCCAATGCCAATTTTCTGAATTCCAATAGA
AATATTATTTACAAAATAATGGGAAAAATTAAATTTTGTACGAATTCCAATGCCAATTTTCTGAATTCCAATAGA
AATATTTTTTCCAAAATAATGGGAAAAATTAAATTTTGTACGAATTCCAATGCCAATTTTCTGAATTCCAATAGA
****** *** ****************************************************************
AAAAATGAGTAAATCATTGTAACAATAAGCAGAAAGGGCAAATACAAAAATCTGAGAAGATTTATTCTAGAGAAG
AAAAATGAGTAAATCATTGTAACAATAAGCAGAAAGGGCAAATACAAAAATCTGAGAAGATTTATTCTAGAGAAG
AAAAATGAGTAAATCATTGTAACAATAAGCAGAAAGGGCAAATACAAAAATCTGAGAAGATTTATTCTAGAGAAG
***************************************************************************
AAAAATTCAATTTCTAAATCCTAACTTATTCATTTATTTTGTAATAAATGATTAAATGAACTACAGGTGATCGTT
AAAAATTCAATTTCTAAATCCTAACTTATTCATTTATTTTGTAATAAATGATTAAATGAACTACAGGTGATCGTT
AAAAATTCAATTTCTAAATCCTAACTTATTCATTTATTTTGTAATAAATGATTAAATGAACTACAGGTGATCGTT
***************************************************************************
ACTGGAGATTAACCTCAGATGGTGTAGCAGTCGGCTATCCCAAGCTCATTTCACACTCATGGAAAGGATTGCCAG
ACTGGAGATTAACCTCAGATGGTGTAGCAGTCGGCTATCCCAAGCTCATTTCACACTCATGGAAAGGATTGCCAG
ACTGGAGATTAACCTCAGATGGTGTAGCAGTCGGCTATCCCAAGCTCATTTCACACTCATGGAAAGGATTGCCAG
***************************************************************************
GGAACATAGACGCTGCGTTCACGTACAAAAATGGGAAGACTTACTTCTTCAAGGTATGAATAAAAACCAATAAAT
GGAACATAGACGCTGCGTTCACGTACAAAAATGGGAAGACTTACTTCTTCAAGGTATGAATAAAAACCAATAAAT
GGAACATAGACGCTGCGTTCACGTACAAAAATGGGAAGACTTACTTCTTCAAGGTATGAATAAAAACCAATAAAT
***************************************************************************
GATACAACTATCATTATTTCGTTAGATAATTTCTTAGATACAAGACCAAGAATAAAACGGAGAATATAGATGGAA
GATACAACTATCATTATTTCGTTAGATAATTTCTTAGATACAAGACCAAGAATAAAACGGAGAATATAGATGGAA
GATACAACTATCATTATTTCGTTAGATAATTTCTTAGATACAAGACCAAGAATAAAACGGAGAATATAGATGGAA
***************************************************************************
CGTAAAAGAAGAAATAAATGATATTGTTGCAGGGCTCGAAATACTGGAGATACGTGGGCAAAAAGATGGATGGAG
CGTAAAAGAAGAAATAAATGATATTGTTGCAGGGCTCGAAATACTGGAGATACGTGGGCAAAAAGATGGATGGAG
CGTAAAAGAAGAAATAAATGATATTGTTGCAGGGCTCGAAATACTGGAGATACGTGGGCAAAAAGATGGATGGAG
***************************************************************************
ACTATCCGAAAGAAATCAGCGAAGGTTTTACAGGAATTCCGGATAATATCGACACTGTGACCGTTTGGACCGGAA
ACTATCCGAAAGAAATCAGCGAAGGTTTTACAGGAATTCCGGATAATATCGACACTGTGACCGTTTGGACCGGAA
ACTATCCGAAAGAAATCAGCGAAGGTTTTACAGGAATTCCGGATAATATCGACACTGTGACCGTTTGGACCGGAA
***************************************************************************
ATGGCAAACTCTACTTCTACAAAGGTAGCAAATTCTGGAGGTTTGACCCATCCCAGAAACCACCTGTGAAGAACA
ATGGCAAACTCTACTTCTACAAAGGTAGCAAATTCTGGAGGTTTGACCCATCCCAGAAACCACCTGTGAAGAACA
ATGGCAAACTCTACTTCTACAAAGGTAGCAAATTCTGGAGGTTTGACCCATCCCAGAAACCACCTGTGAAGAACA
***************************************************************************
CTTATCCCAAACTGATCTCGAATTGGGAAGGGCTTCCCGATAATCTTGATGCTTCGATAGTCTATCGTGGTTACA
CTTATCCCAAACTGATCTCGAATTGGGAAGGGCTTCCCGATAATCTTGATGCTTCGATAGTCTATCGTGGTTACA
CTTATCCCAAACTGATCTCGAATTGGGAAGGGCTTCCCGATAATCTTGATGCTTCGATAGTCTATCGTGGTTACA
***************************************************************************
CGTATTTCTTCAAAGGAGATGCTTATTATCGATTCAACGATAGGACGTTTTCCGTGAGTACTCAAATGATTTTAT
CGTATTTCTTCAAAGGAGATGCTTATTATCGATTCAACGATAGGACGTTTTCCGTGAGTACTCAAATGATTTTAT
CGTATTTCTTCAAAGGAGATGCTTATTATCGATTCAACGATAGGACGTTTTCCGTGAGTACTCAAATGATTTTAT
***************************************************************************
GTTTTTTTTTTCAAATTTTTTGATAAAAAGTTTTATAATATACGATAAAAAGATTGGAAATTGTTTTTAATAAAA
GTTTTTTTTTTCAAATTTTTTGATAAAAAGTTTTAAAA
GTTTTTTTTTTCAAATTTTTTGATAAAAAGTTTTATAATATACGATAAAAAGATTGGAAATTGTTTTTAATAAAA
***************************************************************************
GAAGAATATAAAAGTCATAACTCAACTCAACATAATAAAAATAACACGTTCAGAATAATTGAAAAATAAAAAAAA
GAAGAATATAAAAGTCATAACTCAACTCAACATAATAAAAATAACACGTTCAGAATAATTGAAAAATAAAAAAAA
***************************************************************************
TTATCAAAATTATTTTGGATCGTACTAACCCAATTACTTCTAAATAAATAAATAAAATAATTAGAAATAAGAAAA
TTATCAAAATTATTTTGGATCGTACTAACCCAATTACTTCTAAATAAATAAATAAAATAATTAGAAATAAGAAAA
                                                      AAATAATTAGAAATAAGAAAA
***************************************************************************
AATACCTTTGATTAAAATTGTTTTAAATACTGTTTAGGTGGACATTGCTGATCCAGCATTCCCTAGGTCGACAGC
AATACCTTTGATTAAAATTGTTTTAAATACTGTTTAGGTGGACATTGC
```

Commented [AB18]: Sequenzierung 04.12.25
Seq. ID KPV877
Exon3-9 Revers

Commented [AB19]: Primer F_In8

Commented [AB20]: Sequenzierung 11.12.25
Seq. ID KPV883
Exon4-10 For_In8

Commented [AB21]: Primer R_Ex9

```
AATACCTTTGATTAAA-TTGTTTTAAATACTGTTTAGGTGGACATTGCTGATCCAGCATTCCCTAGGTCGACAGC
**************** **********************************************************

TTACTGGTGGTTCGGATGCAGATCGGCCAACAAGGGAACGTTAGGTAATGGTTCAATGAATCGATGGTACAATAG
TTACTGGTGGTTCGGATGCAGATCGGCCAACAAGGGAACGTTAGGTAATGGTTCAATGAATCGATGGTACAATAG
***************************************************************************

CCAATGTAATGGCAACGAATGCCTATGGTTTCCTGAAAATGCTATTAATACGTTTCTGAATGGCGGAGATTTCTT
CCAATGTAATGGCAACGAATGCCTATGGTTTCCTGAAAATGCTATTAATACGTTTCTGAATGGCGGAGATTTCTT
***************************************************************************

CATTCCGGACTCCGTTGATGGTGACAATAACGACGTCGGAGACATCATATTAGATGCAGGTATTAAAGCTTCATT
CATTCCGGACTCCGTTGATGGTGACAATAACGACGTCGGAGACATCATATTAGATGCAGGTATTAAAGCTTCATT
***************************************************************************

CGAGTCAGACCAGATTCTTACCTCGCCATAGTACAGTTTCATAGCATCCACTTTTTTTTTATGATGTACATAAAA
CGAGTCAGACCAGATTCTTACCTCGCCATAGTACAGTTTCATAGCATCCACTTTTTTTTTATGATGTACATAAAA
***************************************************************************

TATATATTTAAATATATTAAGTATATAGATATATAAATATTAATATAATTTATATTTCATTATATTTTATCTTTA
TATATATTTAAATATATTAAGTATATAGATATATAAATATTAATATAATTTATATTTCATTATATTTTATCTTTA
***************************************************************************

TAAGGAAAATTTTAAATATAGAATATTTTGTATTTAGATTCTTTTTAAATATTTTTAATTTTTGTAATTTTCCAA
TAAGGAAAATTTTAAATATAGAATATTTTGTATTTAGATTCTTTTTAAATATTTTTAATTTTTGTAATTTTCCAA
***************************************************************************

AACTAAGATTGTATTTGTGATTTTTCTAAAAGTGGATACTTGAAGCTTCGAACTTATCTCACCACTACGAAAAAT
AACTAAGATTGTATTTGTGATTTTTCTAAAAGTGGATACTTGAAGCTTCGAACTTATCTCACCACTACGAAAAAT
***************************************************************************

CGAATTTTAAGTGGTAATAAGTAAAATATATAGAAAGATATAAAAAAGGCGAGGATACAGTATTTGGTGTCTCGA
CGAATTTTAAGTGGTAATAAGTAAAATATATAGAAAGATATAAAAAAGGCGAGGATACAGTATTTGGTGTCTCGA
***************************************************************************

ACACCTGGCTATTACATACCTAGGGCTTTTATAACCATCGAAGAAATTTGTGCATGCAATAATAATTATCTGATA
ACACCTGGCTATTACATACCTAGGGCTTTTATAACCATCGAAGAAATTTGTGCATGCAATAATAATTATCTGATA
***************************************************************************

ATTTAAAATAAAATTTTATCACGAGAAGTTAAACGGATATTAGAAAATTATAATTTAAAGTAAAATAAGAAACAA
ATTTAAAATAAAATTTTATCACGAGAAGTTAAACGGATATTAGAAAATTATAATTTAAAGTAAAATAAGAAACAA
***************************************************************************

TCGTTTCTCAACAGCTCTTCCACAGGAGACTAAAATTATTATTACACACGTTCAATTTTAATACAACATGCCACA
TCGTTTCTCAACAGCTCTTCCACAGGAGACTAAAATTATTATTACACACGTTCAATTTTAATACAACATGCCACA
***************************************************************************

CATTTCATAATTTATATTTTAATTCATTGCCTTGTAATACAAAATCGCGTTAACATATAATTATGACTTAGATAT
CATTTCATAATTTATATTTTAATTCATTGCCTTGTAATACAAAATCGCGTTAACATATAATTATGACTTAGATAT
***************************************************************************

AATGATATTAAATGATTATAATTAAATTTTGAGTTCTATTGATCATTATTTAACGCATTATGTCATTATTATTTA
AATGATATTAAATGATTATAATTAAATTTTGAGTTCTATTGATCATTATTTAACGCATTATGTCATTATTATTTA
***************************************************************************

TTTAACGCAACATGATTAATAATAATATAATGTGGCTTGCACAAAATTCTTCGTGAGTATAATAATATTTACAAA
TTTAACGCA-CATGATTAATAATAATATAATGTGGCTTGCACAAAATTCTTCG
********* *******************************************

CATGGTGGTCATCTTAATTTGGGATATTTCATGGTGGAAGGTAATTAACATTGAAACCAAAAAATAATTTAATTA
TGAATATACCATTGGATCTTCGATGCCATGCAGCATATAACATATTAATTTGCTAACAAAAAACATAAAATCGCT
TTAACACATCCATTATTCATCCATTTTTATTAATTCATTATCTAAATTGCACATCAACAAAAATATTAATATAAT
TGTAATTAATATAATTGTCATTAAATTATACAATATTTTATTATTTTATACAATAATGATATTTTTAAAAGCTTT
AAGCAAATAAAATTGCTTAGATTTTTAATTAACCTTTAAGAAAAATCAATTTTTACTATAACAAATATTTTTTTC
CAGAGTATGTACTGATCTCCATGTACATCAGTTTTATTTTTATTATTATTGAAAATTGGATTATTTTATTTTTGT
CAATGTGCAAAATTGTCATTTGATTTTACCCTTATGAGCCTTAGGATAACTTCCATGGAATTTTTCATGATTGAA
AATTAAAAATTCTATCAAATTCCCAAAATAAAATTAAATTTACTTGTTTAAATTCAGATTAAATACCGCAACGAA
ATATCATCTTTTTTAAATAAATTATATAAAACGGAAATTAAAAAAAACAATTTACAAATTCTGTCACAGCATTGG
AAGTTTTCCTAAGACTTATCCTTACGCATAAATACATATCCTATGATCCTTATTCTCCAAAGAAATTGTTATACA
AATGAATCATTCGCGAAATTATATTCGCATGCTTGTCGAAGCCCAGAGTATTTATTAGCCAGGCAAAAAAAAAAA
TGAAGTTTTATGTATCTGCATAAAAAAACTCAAAACAAAAAGAGAAACCAAAGAAAGAATAGAAATTTTCCATTC
GGTATAATTAATGTGACAGAAATAATGTAAAAACGTCTGTCACCATCATCGGATCCAGTTTGATGGAAACAATAA
ATATTACAACACAAGCTTTATCTATTAAATTTCTCTTTTGTTTCTTTTTCTTTTTTTTTTTTTTTTTTGCTTATT
TATGTGATCGCATTTTATCCACGAATTATTTTATTTGTAGTTATGTAGTTTTTTACCTTTTTTTTTTTTACCTATT
TTATACGTCGTACATTCTTCTTGCTCCAAATTTAAATATTCATAGGTATATCATATTCCTACTTGCTCACTTACG
AGAATAAATTCGCACCATGATCTCTTTCATCAGATAAATGAATTTCGCTTCTGCACGTTTTTTTTTTTCTCTCGA
```

```
TTTTATTTACTAATATTTTCAATCGCTTGTAAAGCAAAAAATAATACTGTCTTCAGGTTCGAGCTTTTTATTATC
TTAAAACCTATATGGAACTATATCAATCGATATCGCTTTCGACAGCTTTCAATGATTTCACGAACAGAATATTTT
CACCGAGTTAAATCAGGTCTGTCAACGCCAGCTCTAAGCGTCAAATTATCCGTTTATCATATGATGAACACAAAT
AATCAGTTATTTGAATAATGGAATATAAACCTGTGAAATTTTAAACACGAAATCGCATGGACAGAGTTCATTTGA
CAGACCTGTGTGTGAAATCAAAATCACTGCGACGCATGATGATAGATATCCATTTATTGATATCTATTGCACGAA
                                   GATAGATATCCATTTATTGATATCTATTGCACGAA
                                                                   GAA
                                                                   ***

TTCTTTTTCTTTTTTTTTTTTTTTGCATATAATTACGAAGGATGTATCACAGCACGAAACACATAAAACATGCGC
TTCTTTTTCTTTTTTTTTTTTTTTGCATATAATTACGAAGGATGTATCACAGCACGAAACACATAAAACATGCGC
TTCTTTTTCTTTTTTTTTTTTTTTGCATATAATTACGAAGGATGTATCACAGCACGAAACACATAAAACATGCGC
**********************************************************************

ACATGCACGAATCTCCGCGCAAACGTATGAAGCAAACAGGTGCATGGTAAAAACTATGTATATGGAGGATGGTTA
ACATGCACGAATCTCCGCGCAAACGTATGAAGCAAACAGGTG
ACATGCACGAATCTCCGCGCAAACGTATGAAGCAAACAGGTGCATGGTAAAAACTATGTATATGGAGGATGGTTA
**********************************************************************

CGTTGTTTGGAACTTAATCTATCATAGAAAATTGTCTAATCCCGTCTTCCCTTTTAACCTTTTTCAAATAAATAA
CGTTGTTTGGAACTTAATCTATCATAGAAAATTGTCTAATCCCGTCTTCCCTTTTAACCTTTTTCAAATAAATAA
**********************************************************************

AATCAATCTTCATTTCCTATATTTCTCGATTTCCTCTGTCTCGTTTCTTCCTTTTTTGACATTTCTTTTTCTTTT
AATCAATCTTCATTTCCTATATTTCTCGATTTCCTCTGTCTCGTTTCTTCCTTTTTTGACATTTCTTTTTCTTTT
**********************************************************************

CTTTTTTTTTTCTACACGTACAAATACATACATCGTACACAAAAATGCACGTACTTTTGTTTATTATCTTTTATA
CTTTTTTTTTTCTACACGTACAAATACATACATCGTACACAAAAATGCACGTACTTTTGTTTATTATCTTTTATA
**********************************************************************

TTTATACAATTTATTTGCACGAGTATTTCGTTCTATTTTTTAGTTCTAAATAATCTTTCCCCACACAGAATCATT
TTTATACAATTTATTTGCACGAGTATTTCGTTCTATTTTTTAGTTCTAAATAATCTTTCCCCACACAGAATCATT
**********************************************************************

TGGTACAGAGCATGTTGTATTTTCTTTTCTTTCTGCACTCATTTTCTTTTATTCTCGGGTTTATTTCTGTTTTGG
TGGTACAGAGCATGTTGTATTTTCTTTTCTTTCTGCACTCATTTTCTTTTATTCTCGGGTTTATTTCTGTTTTGG
**********************************************************************

TAAAGTCTATGTGGAAGTTGCTAAATATCTACTATATTATAGTAATATTCCCCTTAATGCTCTGCACTTATTGTA
TAAAGTCTATGTGGAAGTTGCTAAATATCTACTATATTATAGTAATATTCCCCTTAATGCTCTGCACTTATTGTA
**********************************************************************

AGATATCTCTCTTTGAAGAAAAATATAAGTTTCTTTTTTTTTCGATAATGAACAATATTAACTGATTTGAAATTCT
       TCTCTT-GAAGAAAAAT-TAAGTTTCTTTTTTTTTCGATAATGAACAATATTAACTGATTTGAAATTCT
AGATATCTCTCTTTGAAGAAAAATATAAGTTTCTTTTTTTTTCGATAATGAACAATATTAACTGATTTGAAATTCT
       ****** ********** *********************************************

ACATTTTAAAAAATTATTCCTAGCTTGATAGAACAATTTAAATTTTAATTTATTTCTCTAAATTTATTAAAATAA
ACATTTTAAAAAATTATTCCTAGCTTGATAGAACAATTTAAATTTTAATTTATTTCTCTAAATTTATTAAAATAA
ACATTTTAAAAAATTATTCCTAGCTTGATAGAACAATTTAAATTTTAATTTATTTCTCTAAATTTATTAAAATAA
**********************************************************************

GAATACTTATTAATTATCTTCTGAAGATTTTTGTGCATAAAAGAAATATACTTTCTCACAGATATTTCTACATTT
GAATACTTATTAATTATCTTCTGAAGATTTTTGTGCATAAAAGAAATATACTTTCTCACAGATATTTCTACATTT
GAATACTTATTAATTATCTTCTGAAGATTTTTGTGCATAAAAGAAATATACTT
**********************************************************************

ACAATTATCGCATATTGTTTCTTCTTTCAAACGACTTTACTTTTAATAAGCAAAATATTAAAAATTCAACAATCT
ACAATTATCGCATATTGTTTCTTCTTTCAAACGACTTTACTTTTAATAAGCAAAATATTAAAAATTCAACAATCT
**********************************************************************

CAAAGTTTGATCTTTTCATCAATAAATTTGCATTTTTTTACATTTACGTAATTTATCCAAATAGGAAAAAAGAAC
CAAAGTTTGATCTTTTCATCAATAAATTTGCATTTTTTTACATTTACGTAATTTATCCAAATAGGAAAAAAGAAC
**********************************************************************

ATTAGAAAATTCATAATCAACAAAATGAGTAACTGTCCACAAGACTCGTGTTATTTATCTTTGTTTTACACTTTT
ATTAGAAAATTCATAATCAACAAAATGAGTAACTGTCCACAAGACTCGTGTTATTTATCTTTGTTTTACACTTTT
**********************************************************************

GTTTTTGTTAAAGCACACTTTTTATTTAAAATTATAATATATATAAGTTATAATTTATTCTCACATTATTAATCT
GTTTTTGTTAAAGCACACTTTTTATTTAAAATTATAATATATATAAGTTATAATTTATTCTCACATTATTAATCT
**********************************************************************

TCGATGATAACAACAACGCAAACATGAATCACACATATTCTCACATATTTTCTCGAATTCTGAAAAAAAAATTTT
TCGATGATAACAACAACGCAAACATGAATCACACATATTCTCACATATTTTCTCGAATTCTGAAAAAAAAATTTT
**********************************************************************
```

Commented [AB22]: Sequenzierung 11.12.25
Seq. ID KPV884
Exon4-10 Rev_In9

Commented [AB23]: Primer: R_in9b

Commented [AB24]: Sequenzierung 22.01.26
Seq. ID KPV895
Exon4-10 Rev_In9b

Commented [AB25]: Sequenzierung 04.12.25
Seq. ID KPV879
Exon4-10 Revers

Commented [AB26]: Primer R_In9

```
TTGAAAATTAACAATTGATAATCAAAATTTAATCAAAAGCACAAAAATCTTGAGATTTACCTTAATACTTCGATG
TTGAAAATTAACAATTGATAATCAAAATTTAATCAAAAGCACAAAAATCTTGAGATTTACCTTAATACTTCGATG
***************************************************************************
ATATAAATAACGATGGCGAGAAATTTCGAGAAGAACGATCACGGGAACGTACGTTTCGCGATAACGAAAAATAAC
ATATAAATAACGATGGCGAGAAATTTCGAGAAGAACGATCACGGGAACGTACGTTTCGCGATAACGAAAAATAAC
***************************************************************************
GAAATTCCATTAACCTGAAAAAACCTCACTTTTCCAGAAGACAACAATCTGTATTTTGAATCGCACGGTTACGGG
GAAATTCCATTAACCTGAAAAAACCTCACTTTTCCAGAAGACAACAATCTGTATTTTGAATCGCACGGTTACGGG
***************************************************************************
AAAAAGTGAACGGACAGGAATGAAAACTTGTTCGTTCGAGGAAATCTCGTTGAATCGTAAACTCGAGGTTAGGAG
AAAAAGTGAACGGACAGGAATGAAAACTTGTTCGTTCGAGGAAATCTCGTTGAATCGTAAACTCGAGGTTAGGAG
***************************************************************************
GCAACAAAATGGGACAAGAAATTTTCAAGGCGAAAATATCATAAAAATCGAGACACAGAGATCACGAGGGGAAAC
GCAACAAAATGGGACAAGAAATTTTCAAGGCGAAAATATCATAAAAATCGAGACACAGAGATCACGAGGGGAAAC
***************************************************************************
GTTCAGGAAAAAGCCTAGATCTATCTAATGGCGTAACTGATAAGTTCCAAACAACAGAAAACGATTGTTCAACCG
GTTCAGGAAAAAGCCTAGATCTATCTAATGGCGTAACTGATAAGTTCCAAACAACAGAAAACGATTGTTCAACCG
***************************************************************************
TAGTGCTGTTTCACGCGAGATAGATCGTGTTGAGCGTGTTTGGTGCTGAAGTGAACCATCCATCGATCAGCGACG
TAGTGCTGTTTCACGCGAGATAGATCGTGTTGAGCGTGTTTGGTGCTGAAGTGAACCATCCATCGATCAGCGACG
***************************************************************************
ATCCTCTTATAATATTGTACATACAATTTTTTCAATGTCGACCCTCCTACGATTTTTAAACAATTCGACAGGCGA
ATCCTCTTATAATATTGTACATACAATTTTT-CAATGTCGACCCTCCTACGATTT
******************************* ***********************
AACGGACGAGCAGTTAGTTACGTCTTCCAATCAGGATATGGCTCCGCGGCGGGAAACGTTTTCACATCGTCATC
GATATTGTGGTATCTTCCGCTCAATTTGATGACCATATTCATCGTTAAGATTGTTGCCACTACGTAAAAAAAAAA
AAAAAACAGAGATGAATTACTTGCTGGAGATCGTAAAGCGTTGATATCATCAACGAAGGTCCGATAGAAAATATT
TGAAAAAAACGGACAATTAAAACGGA
```

Commented [AB27]: R10

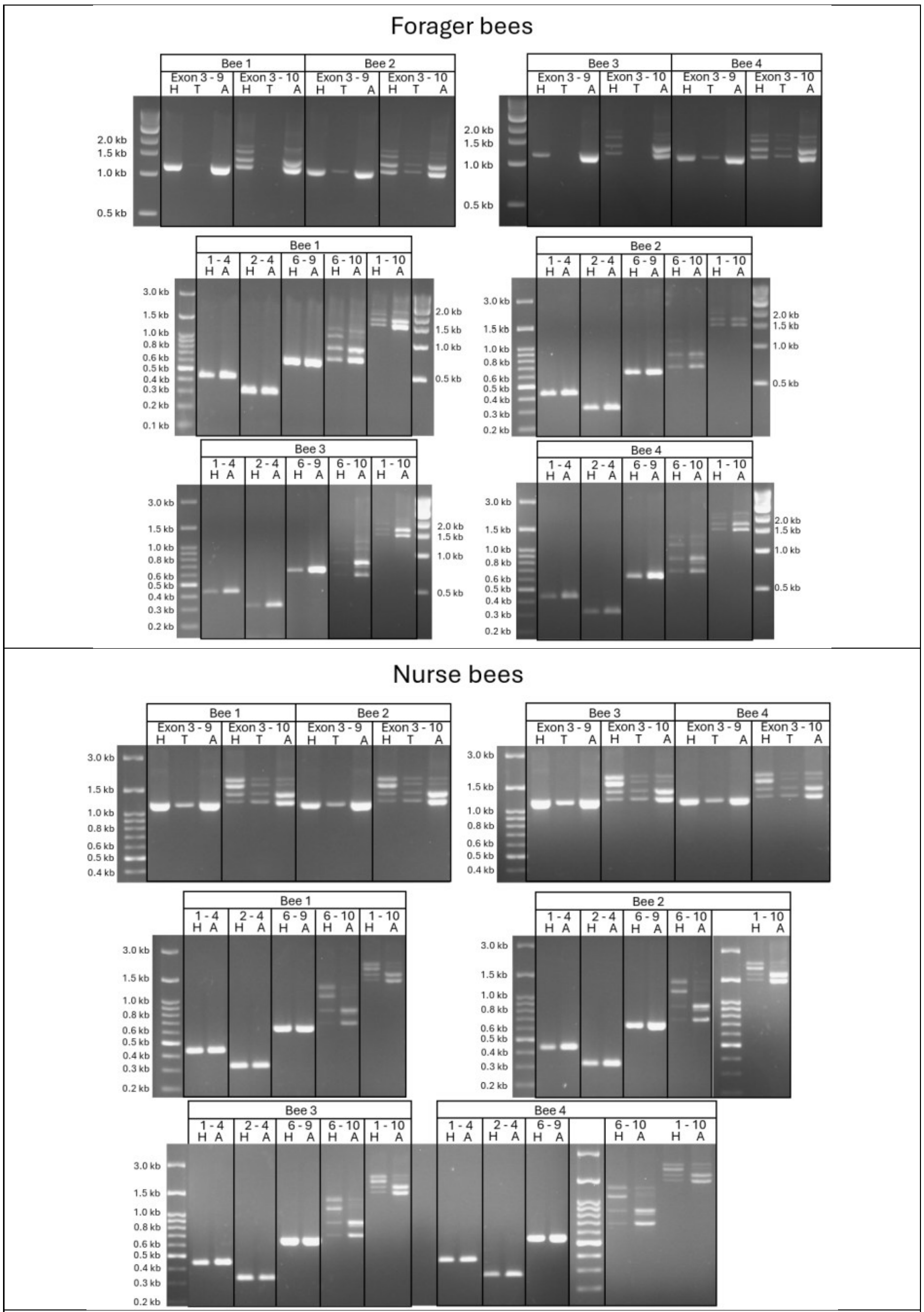

Figure S2: Agarose gels (1.5%, TBE buffer, 100 V, 2 h) showing PCR products of *mmp_LOC409868* amplification from cDNA (RT-PCR) of four different forager bees. H, head; T, thorax; A, abdomen. As expression in thoraces was barely detectable for the first two primer pairs (3-9 and 3-10), thorax cDNA was omitted as template in all other PCRs.

Figure S3: Alignment of Sanger sequencing of cDNA PCR products including different isoforms with genomic sequence (JBGNXS010000264.1) covering Amel_4.5_LOC409868 (Elsik et al., 2014) published within BioProject PRJNA1026921 (Gilligan et al., 2024, bioRxiv). Blue: exons deduced from sequenced cDNA PCR products; yellow: introns deduced from sequenced cDNA PCR products, long introns have been shortened using dashes; red: potential start and stop codon. Underlined, bold, sequences in italics are primer sequences.

```
TGCTTGGAATAATTGATTGACGGGATGATGGCCAGGTGGAATGGTATTTTAAGCTGGAAAAGATGTGCCGGAAGT
TGTGCCCTGTTGGCGACTCTGTTTGTCGTTTCGCGGGCCGAGGAGGAGGAGAAGACTGGCATATCGCAGACCGCG
                                             GA-GAG-AGACTGGCATATCGCAGACCGCG
            GCG-CTCTGTTTGTCGTTTCGCGGGCCGAGGAGGAGGAGAAGACTGGCATATCGCAGACCGCG
GCAATGGTAAGCACTTATTATGATCAATTTTTCCTTTCTCTCTTTCTTTCGCTTTTTAAGAGAACGATTAGAATT
GCAATG.....................................................................
GCAATG.....................................................................
TAATAGTTAAAAGTTTGTGATGTAGATTTTAGAACGGTTTTAGAAGAGATTTATGATCTAGGAAATAATAATTA
---------------------------------------------------------------------------
TTTACGATATTTTTAATACCAAAAATAATACGAAAATGTCAGTTGAAAAAAAATGTTCAAATAGATTCGATATTT
CTTCGATATAACGAATTATAAATTAAAAAGGAAAAATTATTAAATAAAATTACTATTTATTATCTGTTACAGAAT
........................................................................AAT
........................................................................AAT
TATCTCTCTCAATTTGGATATTTACAACCTATGAATCCAACAAGTGGTGGAATCATATCCGAGGAAACACTTTCT
TATCTCTCTCAATTTGGATATTTACAACCTATGAATCCAACAAGTGGTGGAATCATATCCGAGGAAACACTTTCT
TATCTCTCTCAATTTGGATATTTACAACCTATGAATCCAACAAGTGGTGGAATCATATCCGAGGAAACACTTTCT
AAAGCAATCTCGGAATTTCAAGCTTTTGCCGGATTGAATATAACAGGTAAGTCTGATATTTCAATTTTTATAACA
AAAGCAATCTCGGAATTTCAAGCTTTTGCCGGATTGAATATAACAG.............................
AAAGCAATCTCGGAATTTCAAGCTTTTGCCGGATTGAATATAACAG.............................
TATTTTTTAACATTACAGTAATTAAAATTTACAAAGAAACGATAAAAGTTTAATTAAAAAATTCTACAATAAAAT
---------------------------------------------------------------------------
AAAAAAAATATAATTTAATTTTATCTTAAAAATTAAATTATAAGCAATGTTAAAAATTTATTATGTTATATTTGT
AATTATTATTGAAAAAATTGTTTTTCCTTTTTTTTAGGTGATATCGATGAAGAAACATATAAATTAATGACATT
......................................GTGATATCGATGAAGAAACATATAAATTAATGACATT
......................................GTGATATCGATGAAGAAACATATAAATTAATGACATT
GCCAAGGTGTGGTGTTAAAGACAAAGTTGGTCCTGGCTTTGGAAGATCGAAGCGATACGCTCTTCAAGGTAAATC
GCCAAGGTGTGGTGTTAAAGACAAAGTTGGTCCTGGCTTTGGAAGATCGAAGCGATACGCTCTTCAAG.......
GCCAAGGTGTGGTGTTAAAGACAAAGTTGGTCCTGGCTTTGGAAGATCGAAGCGATACGCTCTTCAAG.......
AAATTATAATTTAATTCCATAAAATGGAAAAATTTAATTCTATGAATATCTTTTCATGCAATGCAAATATTTTCA
---------------------------------------------------------------------------
ATCTAATACATTGTTATCAAAATATATATTTTGCATATACAATTTTCATAATCATAAATCGGAATGTTTAATGTA
ATAGTTGATAGAAGAATCGAAAGAAAAAGAAAAGAAATATTTTATTACAGGCTCTAGATGGCGTGTGAAGAAATT
..................................................GCTCTAGATGGCGTGTGAAGAAATT
..................................................GCTCTAGATGGCGTGTGAAGAAATT
                                                              GTGTGAAG-AATT
                                                              GTGTGAAG-AATT
GTCGTACAAG-ATCAGCAAGTATCCGCGAAATTTGCCACAACATAAAGTTGATGCCGAGTTGAACAAAGCGTTCAA
GTCGTACAAG-ATCAGCAAGTATCCGCGAAATTTGCCACAACATAAAGTTGATGCCGAGTTGAACAAAGCGTTCAA
GTCGTACAAG-ATCAGCAAGTATCCGCGAAATTTGCCACAACATAAAGTTGATGCCGAGTTGAACAAAGCGT-CAA
GTCGTACAAG-ATCAGCAAGTATCCGCGAAATTTGCCACAACATAAAGTTGATGCCGAGTTGAACAAAGCGTTCAA
GTCGTACAAGTATCAGCAAATATCCGCGAAATTTGCCACAACATAAAGTTGATGCCGAGTTGAACAAGGCGTTCAA
GTCGTACAAGTATCAGCAAGTATCCGCGAAATTTGCCACAACATAAAGTTGATGCCGAGTTGAACAAAGCGTTCAA
                      TCCGCGAAATTTGCCACAACATAAAGTTGATGCCGAGTTGAACAAAGCGTTCAA
AGTATGGTCGGAGTACACGGATCTCGTTTTCGTCCAAAAGAAGTCAGGCCAGGTGAGACGAATAATATTTACCGA
AGTATGGTCGGAGTACACGGATCTCGTTTTCGTCCAAAAG
AGTATG-TC-GAG
AGTATGGTCGGAGTACACGGATCTCGTTTTCGTCCAAAAGAAGTCAGGCCAG.......................
AGTATGGTCGGAGTACACGGATCTCGTTTTCGTCCAAAAGAAGTCAGGCCAG.......................
AGTATGGTCGGAGTACACGGATCTCGTTTTCGTCCAAAAGAAGTCAGGCCAG.......................
AGTATGGTCGGAGTACACGGATCTCGTTTTCGTCCAAAAGAAGTCAGGCCAG.......................
GATATTTCACAATCATGAAGTGAAAAAGAAATGAAAAGCAAAGAATAGTGATCTTATAGCTTATAGCTAAAAATC
---------------------------------------------------------------------------
TGTCTGAAATATCTGAAAACATTTCGATAAAATTCTTATAGATCCTTCGAATAATTTTAACTTTTTAGATTGTAA
CAATTTTCAATCCACTTTTGTTAGGTCCATATCGAAATTAGATTCGAGAAAGGCGAACACGGGGACGGAGATCCT
```

Commented [AB28]: F1

Commented [AB29]: black = sequencing PCR product exon 1-4
Primer: F1, R4
KPV893, KPV892

Commented [AB30]: F2 + R2

Commented [AB31]: Red = sequencing 1.4 kb variant (Ex 3-10)
Primer: F3, F6, R10
KPV885, KPV886, KPV889

Commented [AB32]: Green = sequencing 1.2 kb variant (Ex 3-10)
Primer: F3, F6, R10
KPV887, KPV888, KPV890

Commented [AB33]: blue = sequencing PCR product exon 3-9
Primer: F3
EHC976

```
.......................GTCCATATCGAAATTAGATTCGAGAAAGGCGAACACGGGGACGGAGATCCT
.......................GTCCATATCGAAATTAGATTCGAGAAAGGCGAACACGGGGACGGAGATCCT
.......................GTCCATATCGAAATTAGATTCGAGAAAGGCGAACACGGGGACGGAGATCCT
.......................GTCCATATCGAAATTAGATTCGAGAAAGGCGAACACGGGGACGGAGATCCT
TTCGATGGACCTGGTGGCACTTTGGCTCACGCTTACTTCCCCGTATACGGTGGTGATGCTCACTTCGACGACGCA
TTCGATGGACCTGGTGGCACTTTGGCTCACGCTTACTTCCCCGTATACGGTGGTGATGCTCACTTCGACGACGCA
TTCGATGGACCTGGTGGCACTTTGGCTCACGCTTACTTCCCCGTATACGGTGGTGATGCTCACTTCGACGACGCA
                               CTTACTTCCCCGTATACGGTGGTGATGCTCACTTCGACGACGCA
TTCGATGGACCTGGTGGCACTTTGGCTCACGCTTACTTCCCCGTATACGGTGGTGATGCTCACTTCGACGACGCA
TTCGATGGACCTGGTGGCACTTTGGCTCACGCTTACTTCCCCGTATACGGTGGTGATGCTCACTTCGACGACGCA
GAA-CAATGGA-CCATCGACAGTTTCCGTGGAACGAATCTGTTTCAAGTAGCTGCTCACGAGTTTGGCCATTCTCTC
GAA-CAATGGA-CCATCGACAGTTTCCGTGGAACGAATCTGTTTCAAGTAGCTGCTCACGAGTTTGGCCATTCTCTC
GAA-CAATGGA-CCATCGACAGTTTCCGTGGAACGAATCTGTTTCAAGTAGCTGCTCACGAGTTTGGCCATTCTCTC
GAACCAATGGAACCATCGACAGTTTCCGTGGAACGAATCTGTTTCAAGTAGCTGCTCACGAGTTTGGCCATTCTCTC
GAA-CAATGGA-CCATCGACAGTTTCCGTGGAACGAATCTGTTTCAAGTAGCTGCTCACGAGTTTGGCCATTCTCTC
GAA-CAATGGA-CCATCGACAGTTTCCGTGGAACGAATCTGTTTCAAGTAGCTGCTCACGAGTTTGGCCATTCTCTC
GGTCTGAGCCACAGCGACGTTAAATCCGCTTTAATGGCACCCTTTTACCGCGGCTATGAGCCTTATTTCCGGTTA
GGTCTGAGCCACAGCGACGTTAAATCCGCTTTAATGGCACCCTTTTACCGCGGCTATGAGCCTTATTTCCGGTTA
GGTGTGAGCCACAGCGACGTTAAATCTGCTTTAATGGCACCCTTTTATCGCGGCTATGAACCTTATTTCCGGTTA
GGTCTGAGCCACAGCGACGTTAAATCCGCTTTAATGGCACCCTTTTATCGCGGCTATGAACCTTATTTCCGGTTA
GGTCTGAGCCACAGCGACGTTAAATCCGCTTTAATGGCACCCTTTTACCGCGGCTATGAACCTTATTTCCGGTTA
GGTCTGAGCCACAGCGACGTTAAATCCGCTTTAATGGCACCCTTTTATCGCGGCTATGAGCCTTATTTCCGGTTA
GACGAGGACGATATTCAGGGTATCCAGGTAAATACAAAGTTCAAAATGTTGGGAGAATTAATTTTTCATAAACGA
GACGAGGACGATATTCAGGGTATCCAG...............................................
GACGAGGACGATATTCAGGGTATCCAG...............................................
GACGAGGACGATATTCAGGGTATCCAG...............................................
GACGAGGACGATATTCAGGGTATCCAG...............................................
GACGAGGACGATATTCAGGGTATCCAG...............................................
AGAATGACAGAATTTCTTCGATTTGAGAAAAAAATTGGAAGAATACATTTTGAAATGGAATGGAAATTGATTGAG
AAGAAATATACGAATTGTATAGCACGATGATGAGGCAGAAGAGTTTAAATTTGAACTTTTCGCGTGATTTAGGCT
.......................................................................GCT
.......................................................................GCT
.......................................................................GCT
.......................................................................GCT
.......................................................................GCT
CTGTATGGAACGAAAACGTCAAATAACGGTGGAGTTCCGAGCGGGCCAAAACCAACTACCACTGCATCGCCTAGC
CTGTATGGAACGAAAACGTCAAATAACGGTGGAGTTCCGAGCGGGCCAAAACCAACTACCACTGCATCGCCTAGC
CTGTATGGAACGAAAACGTCAAATAACGGTGGAGTTCCGAGCGGGCCAAAACCAACTACCACTGCATCGCCTAGC
CTGTATGGAACGAAAACGTCAAATAACGGTGGAGTTCCGAGCGGGCCAAAACCAACTACCACTGCATCGCCTAGC
                                                              TGCATCGCCTAGC
CTGTATGGAACGAAAACGTCAAATAACGGTGGAGTTCCGAGCGGGCCAAAACCAACTACCACTGCATCGCCTAGC
CTGTATGGAACGAAAACGTCAAATAACGGTGGAGTTCCGAGCGGGCCAAAACCAACTACCACTGCATCGCCTAGC
                                                          CACTGCATCGCCTAGC
                                                                  CGCCTAGC
GAAGAGGACTCGGAACTGTGCACGAATTCAAAGGTCGATACCATGTTCAATTCTGCGGAGGGACATATGTACGTG
GAAGAGGACTCGGAACTGTGCACGAATTCAAAGGTCGATACCATGTTCAATTCTGCGGAGGGACATATGTACGTG
GAAGAGGACTCGGAACTGTGCACGAATTCAAAGGTCGATACCATGTTCAATTCTGCGGAGGGACATATGTACGTG
GAAGAGGACTCGGAACTGTGCACGAATTCAAAGGTCGATACCATGTTCAATTCTGCGGAGGGACATATGTACGTG
G-AGAGGACTCGGAACTGTGCACGAATTCAAAGGTCGATACCATGTTCAATTCTGCGGAGGGACATATGTACGTG
GAAGAGGACTCGGAACTGTGCACGAATTCAAAGGTCGATACCATGTTCAATTCTGCGGAGGGACATATGTACGTG
GAAGAGGACTCGGAACTGTGCACGAATTCAAAGGTCGATACCATGTTCAATTCTGCGGAGGGACATATGTACGTG
G-AGAGGACTCGGAACTGTGCACGAATTCAAAGGTCGATACCATGTTCAATTCTGCGGAGGGACATATGTACGTG
GAAGAGGACTCGGAACTGTGCACGAATTCAAAGGTCGATACCATGTTCAATTCTGCGGAGGGACATATGTACGTG
TTCAAGGGTACGTATATGTCAGAATTGGTATGATAACGAAATATCTTTATATATATACTATTAATATATTTATAA
TTCAAGG...................................................................
TTCAAGG...................................................................
TTCAAGG...................................................................
TTCAAGG...................................................................
TTCAAGG...................................................................
TTCAAGG...................................................................
TTCAAGG...................................................................
```

Commented [AB34]: Purple = sequencing 1.2 kb variant (Ex 6-10)
Primer: F6,
KPV898

```
TTCAAGG....................................................................
AATAGAATTATATTTATCTAAATTTTATTTTATTCATTGAACTGATCTATCCTCAACTTTCCCTTACTATATAGA
---------------------------------------------------------------------------
AAAAATGAGTAAATCATTGTAACAATAAGCAGAAAGGGCAAATACAAAAATCTGAGAAGATTTATTCTAGAGAAG
AAAAATTCAATTTCTAAATCCTAACTTATTCATTTATTTTGTAATAAATGATTAAATGAACTACAGGTGATCGTT
..................................................................GTGATCGTT
..................................................................GTGATCGTT
..................................................................GTGATCGTT
..................................................................GTGATCGTT
..................................................................GTGATCGTT
..................................................................GTGATCGTT
..................................................................GTGATCGTT
..................................................................GTGATCGTT
ACTGGAGATTAACCTCAGATGGTGTAGCAGTCGGCTATCCCAAGCTCATTTCACACTCATGGAAAGGATTGCCAG
ACTGGAGATTAACCTCAGATGGTGTAGCAGTCGGCTATCCCAAGCTCATTTCACACTCATGGAAAGGATTGCCAG
ACTGGAGATTAACCTCAGATGGTGTAGCAATCGGCTATCCCAAGCTCATTTCACACTCATGGAAAGGATTGCCAG
ACTGGAGATTAACCTCAGATGGTGTAGCAGTCGGCTATCCCAAGCTCATTTCACACTCATGGAAAGGATTGCCAG
ACTGGAGATTAACCTCAGATGGTGTAGCAATCGGCTATCCCAAGCTCATTTCACACTCATGGAAAGGATTGCCAG
ACTGGAGATTAACCTCAGATGGTGTAGCAATCGGCTATCCCAAGCTCATTTCACACTCATGGAAAGGATTGCCAG
ACTGGAGATTAACCTCAGATGGTGTAGCAGTCGGCTATCCCAAGCTCATTTCACACTCATGGAAAGGATTGCCAG
ACTGGAGATTAACCTCAGATGGTGTAGCAGTCGGCTATCCCAAGCTCATTTCACACTCATGGAAAGGATTGCCAG
ACTGGAGATTAACCTCAGATGGTGTAGCAGTCGGCTATCCCAAGCTCATTTCACACTCATGGAAAGGATTGCCAG
GGAACATAGACGCTGCGTTCACGTACAAAAATGGGAAGACTTACTTCTTCAAGGTATGAATAAAAACCAATAAAT
GGAACATAGACGCTGCGTTCACGTACAAAAATGGGAAGACTTACTTCTTCAAG......................
GGAACATAGACGCTGCGTTCACGTACAAAAATGGGAAGACTTACTTCTTCAAG......................
GGAACATAGACGCTGCGTTCACGTACAAAAATGGGAAGACTTACTTCTTCAAG......................
GGAACATAGACGCTGCGTTCACGTACAAAAATGGGAAGACTTACTTCTTCAAG......................
GGAACATAGACGCTGCGTTCACGTACAAAAATGGGAAGACTTACTTCTTCAAG......................
GGAACATAGACGCTGCGTTCACGTACAAAAATGGGAAGACTTACTTCTTCAAG......................
GGAACATAGACGCTGCGTTCACGTACAAAAATGGGAAGACTTACTTCTTCAAG......................
GGAACATAGACGCTGCGTTCACGTACAAAAATGGGAAGACTTACTTCTTCAAG......................
GATACAACTATCATTATTTCGTTAGATAATTTCTTAGATACAAGACCAAGAATAAAACGGAGAATATAGATGGAA
CGTAAAAGAAGAAATAAATGATATTGTTGCAGGGCTCGAAATACTGGAGATACGTGGGCAAAAAGATGGATGGAG
................................GGCTCGAAATACTGGAGATACGTGGGCAAAAAGATGGATGGAG
................................GGCTCGAAATACTGGAGATACGTGGGCAAAAAGATGGATGGAG
................................GGCTCGAAATACTGGAGATACGTGGGCAAAAAGATGGATGGAG
................................GGCTCGAAATACTGGAGATACGTGGGCAAAAAGATGGATGGAG
................................GGCTCGAAATACTGGAGATACGTGGGCAAAAAGATGGATGGAG
................................GGCTCGAAATACTGGAGATACGTGGGCAAAAAGATGGATGGAG
................................GGCTCGAAATACTGGAGATACGTGGGCAAAAAGATGGATGGAG
................................GGCTCGAAATACTGGAGATACGTGGGCAAAAAGATGGATGGAG
ACTATCCGAAAGAAATCAGCGAAGGTTTTACAGGAATTCCGGATAATATCGACACTGTGACCGTTTGGACCGGAA
ACTATCCGAAAGAAATCAGCGAAGGTTTTACAGGAATTCCGGATAATATCGACACTGTGACCGTTTGGACCGGAA
ACTATCCGAAAGAAATCAGCGAAGGTTTTACAGGAATTCCGGATAATATCGACACTGTGACCGTTTGGACCGGAA
ACTATCCGAAAGAAATCAGCGAAGGTTTTACAGGAATTCCGGATAATATCGACACTGTGACCGTTTGGACCGGAA
ACTATCCGAAAGAAATCAGCGAAGGTTTTACAGGAATTCCGGATAATATCGACACTGTGACCGTTTGGACCGGAA
ACTATCCGAAAGAAATCAGCGAAGGTTTTACAGGAATTCCGGATAATATCGACACTGTGACCGTTTGGACCGGAA
ACTATCCGAAAGAAATCAGCGAAGGTTTTACAGGAATTCCGGATAATATCGACACTGTGACCGTTTGGACCGGAA
ACTATCCGAAAGAAATCAGCGAAGGTTTTACAGGAATTCCGGATAATATCGACACTGTGACCGTTTGGACCGGAA
ACTATCCGAAAGAAATCAGCGAAGGTTTTACAGGAATTCCGGATAATATCGACACTGTGACCGTTTGGACCGGAA
ATGGCAAACTCTACTTCTACAAAGGTAGCAAATTCTGGAGGTTTGACCCATCCCAGAAACCACCTGTGAAGAACA
ATGGCAAACTCTACTTCTACAAAGGTAGCAAATTCTGGAGGTTTGACCCATCCCAGAAACCACCTGTGAAGAACA
ATGGCAAACTCTACTTCTACAAGGGTAGCAAATTCTGGAGGTTTGACCCATCCCAGAAACCACCTGTGAAGAACA
ATGGCAAACTCTACTTCTACAAAGGTAGCAAATTCTGGAGGTTTGACCCATCCCAGAAACCACCTGTGAAGAACA
ATGGCAAACTCTACTTCTACAAAGGTAGCAAATTCTGGAGGTTTGACCCATCCCAGAAACCACCTGTGAAGAACA
ATGGCAAACTCTACTTCTACAAAGGTAGCAAATTCTGGAGGTTTGACCCATCCCAGAAACCACCTGTGAAGAACA
ATGGCAAACTCTACTTCTACAAAGGTAGCAAATTCTGGAGGTTTGACCCATCCCAGAAACCACCTGTGAAGAACA
ATGGCAAACTCTACTTCTACAAAGGTAGCAAATTCTGGAGGTTTGACCCATCCCAGAAACCACCTGTGAAGAACA
ATGGCAAACTCTACTTCTACAAAGGTAGCAAATTCTGGAGGTTTGACCCATCCCAGAAACCACCTGTGAAGAACA
CTTATCCCAAACTGATCTCGAATTGGGAAGGGCTTCCCGATAATCTTGATGCTTCGATAGTCTATCGTGGTTACA
CTTATCCCAAACTGATCTCGAATTGGGAAGGGCTTCCCGATAATCTTGATGCTTCGATAGTCTATCGTGGTTACA
```

```
CTTATCCCAAACTGATCTCGAATTGGGAAGGGCTTCCCGATAATCTTGATGCTTCGATAGTCTATCGTGGTTACA
CTTATCCCAAACTGATCTCGAATTGGGAAGGGCTTCCCGATAATCTTGATGCTTCGATAGTCTATCGTGGTTACA
CTTATCCCAAACTGATCTCGAATTGGGAAGGGCTTCCCGATAATCTTGATGCTTCGATAGTCTATCGTGGTTACA
CTTATCCCAAACTGATCTCGAATTGGGAAGGGCTTCCCGATAATCTTGATGCTTCGATAGTCTATCGTGGTTACA
CTTATCCCAAACTGATCTCGAATTGGGAAGGGCTTCCCGATAATCTTGATGCTTCGATAGTCTATCGTGGTTACA
CTTATCCCAAACTGATCTCGAATTGGGAAGGGCTTCCCGATAATCTTGATGCTTCGATAGTCTATCGTGGTTACA
CTTATCCCAAACTGATCTCGAATTGGGAAGGGCTTCCCGATAATCTTGATGCTTCGATAGTCTATCGTGGTTACA
CGTATTTCTTCAAAGGAGATGCTTATTATCGATTCAACGATAGGACGTTTTCCGTGAGTACTCAAATGATTTTAT
CGTATTTCTTCAAAGGAGATGCTTATTATCGATTCA-CGATAGGACGTTTTCC......................
CGTATTTCTTCAAAGGAGATGCTTATTATCGATTCAACGATAGGACGTTTTCC......................
CGTATTTCTTCAAAGGAGATGCTTATTATCGATTCAACGATAGGACGTTTTCC......................
CGTATTTCTTCAAAGGAGATGCTTATTATCGATTCAACGATAGGACGTTTTCC......................
CGTATTTCTTCAAAGGAGATGCTTATTATCGATTCAACGATAGGACGTTTTCC......................
CGTATTTCTTCAAAGGAGATGCTTATTATCGATTCAACGATAGGACGTTTTCC......................
CGTATTTCTTCAAAGGAGATGCTTATTATCGATTCAACGATAGGACGTTTTCC......................
CGTATTTCTTCAAAGGAGATGCTTATTATCGATTCAACGATAGGACGTTTTCC......................
GTTTTTTTTTTCAAATTTTTTGATAAAAAGTTTTATAATATACGATAAAAAGATTGGAAATTGTTTTTAATAAAA
---------------------------------------------------------------------------
TTATCAAAATTATTTTGGATCGTACTAACCCAATTACTTCTAAATAAATAAATAAAATAATTAGAAATAAGAAAA
AATACCTTTGATTAAAATTGTTTTAAATACTGTTTAGGTGGACATTGCTGATCCAGCATTCCCTAGGTCGACAGC
.....................................GTGGAC-TTGCTGAT-CAGCAT
.....................................GTGGAAATTGCTGATCCAGCATTCCCTAGGTCGACAGC
.....................................GTGGACATTGCTGATCCAGCATTCCCTAGGTCGACAGC
.....................................GTGGACATTGCTGATCCAGCATTCCCTAGGTCGACAGC
.....................................GTGGACATTGCTGATCCAGCATTCCCTAGGTCGACAGC
.....................................GTGGACATTGCTGATCCAGCATTCCCTAGGTCGACAGC
.....................................GTGGACATTGCTGATCCAGCATTCCCTAGGTCGACAGC
.....................................GTGGACATTGCTGATCCAGCATTCCCTAGGTCGACAGC
TTACTGGTGGTTCGGATGCAGATCGGCCAACAAGGGAACGTTAGGTAATGGTTCAATGAATCGATGGTACAATAG
TTACTGGTGGTTCGGATGCAGATCGGCCAACA-GGGAACGTTAGGTAA-GGT
TTACTGGTGGTTCGGATGCAGATCGGCCAACAAGGGAACGTTAGGTAATGGTTCAATGAATCGATGGTACAATAG
TTACTGGTGGTTCGGATGCAGATCGGCCAACAAGGGAACGTTAGGTAATGGTTCAATGAATCGATGGTACTATAG
TTACTGGTGGTTCGGATGCAGATCG
TTACTGGTGGTTCGGATGCAGATCG-CCAA
TTACTGGTGGTTCGGATGCAGATCGGCCAACAAGGGAACGTTAG...............................
TTACTGGTGGTTCGGATGCAGATCGGCCAACAAGGGAACGTTAG...............................
CCAATGTAATGGCAACGAATGCCTATGGTTTCCTGAAAATGCTATTAATACGTTTCTGAATGGCGGAGATTTCTT
CCAATGTAATGGCAACGAATGCCTATGGTTTCCTGAAAATGCTATTAATACGTTTCTGAATGGCGGAGATTTCTT
CCAATGTAATGGCAACGAATGCCTATGGTTTCCTGAAAATGCTATTAATACGTTTCTGAATGGCGGAGATTTCTT
...........................................................................
...........................................................................
CATTCCGGACTCCGTTGATGGTGACAATAACGACGTCGGAGACATCATATTAGATGCAGGTATTAAAGCTTCATT
CATTCCGGACTCCGTTGATGGTGACAATAACGAC
CATTCCGGACTCCGTTGATGGTGACAATAACGACGTCGGAGACATCATATTAGATGCAG................
...........................................................................
...........................................................................
CGAGTCAGACCAGATTCTTACCTCGCCATAGTACAGTTTCATAGCATCCACTTTTTTTTTATGATGTACATAAAA
---------------------------------------------------------------------------
ATATAAATAACGATGGCGAGAAATTTCGAGAAGAACGATCACGGGAACGTACGTTTCGCGATAACGAAAAATAAC
GAAATTCCATTAACCTGAAAAAACCTCACTTTTCCAGAAGACAACAATCTGTATTTTGAATCGCACGGTTACGGG
.....................................AAGACAACAATCTGTATTTTGAATCGAACGGTTACGGG
AAAAAGTGAACGGACAGGAATGAAAACTTGTTCGTTCGAGGAAATCTCGTTGAATCGTAAACTCGAGGTTAGGAG
AAAAAGTGAACGGACAGGAATGAAAACTTGTTCGTTCGAGGAAATCTCGTTGAATCGTAAACTCGAGGTTAGGAG
GCAACAAAATGGGACAAGAAATTTTCAAGGCGAAAATATCATAAAAATCGAGACACAGAGATCACGAGGGGAAAC
GCAACAAAATGGGACAAGAAATTTTCAAGGCGAAAATATCATAAAAATCGAGACACAGAGATCACGAGG
GTTCAGGAAAAAGCCTAGATCTATCTAATGGCGTAACTGATAAGTTCCAAACAACAGAAAACGATTGTTCAACCG
TAGTGCTGTTTCACGCGAGATAGATCGTGTTGAGCGTGTTTGGTGCTGAAGTGAACCATCCATCGATCAGCGACG
ATCCTCTTATAATATTGTACATACAATTTTTTCAATGTCGACCCTCCTACGATTTTTAAACAATTCGACAGGCGA
.......................................................................GCGA
.......................................................................GCGA
```

Commented [AB35]: Alternative splice site in isoform 1 and 3.

Commented [AB36]: Exon 10a

A***ACGGACGAGCAGTTAGTTACG***TCTTCCAATCAGGATATGGGCTCCGCGGCGGGAAACGTTTTCACATCGTCATC

AACGGACGAG

AACGGACGAG

GATATTGTGGTATCTTCCGCTCAATTTGATGACCATATTCATCGTTAAGATTGTTGCCACTACGTAAAAAAAAAA
AAAAAACAGAGATGAATTACTTGCTGGAGATCGTAAAGCGTTGATATCATCAACGAAGGTCCGATAGAAAATATT
TGAAAAAAACGGACAATTAAAACGGA

Commented [AB37]: Exon 10b

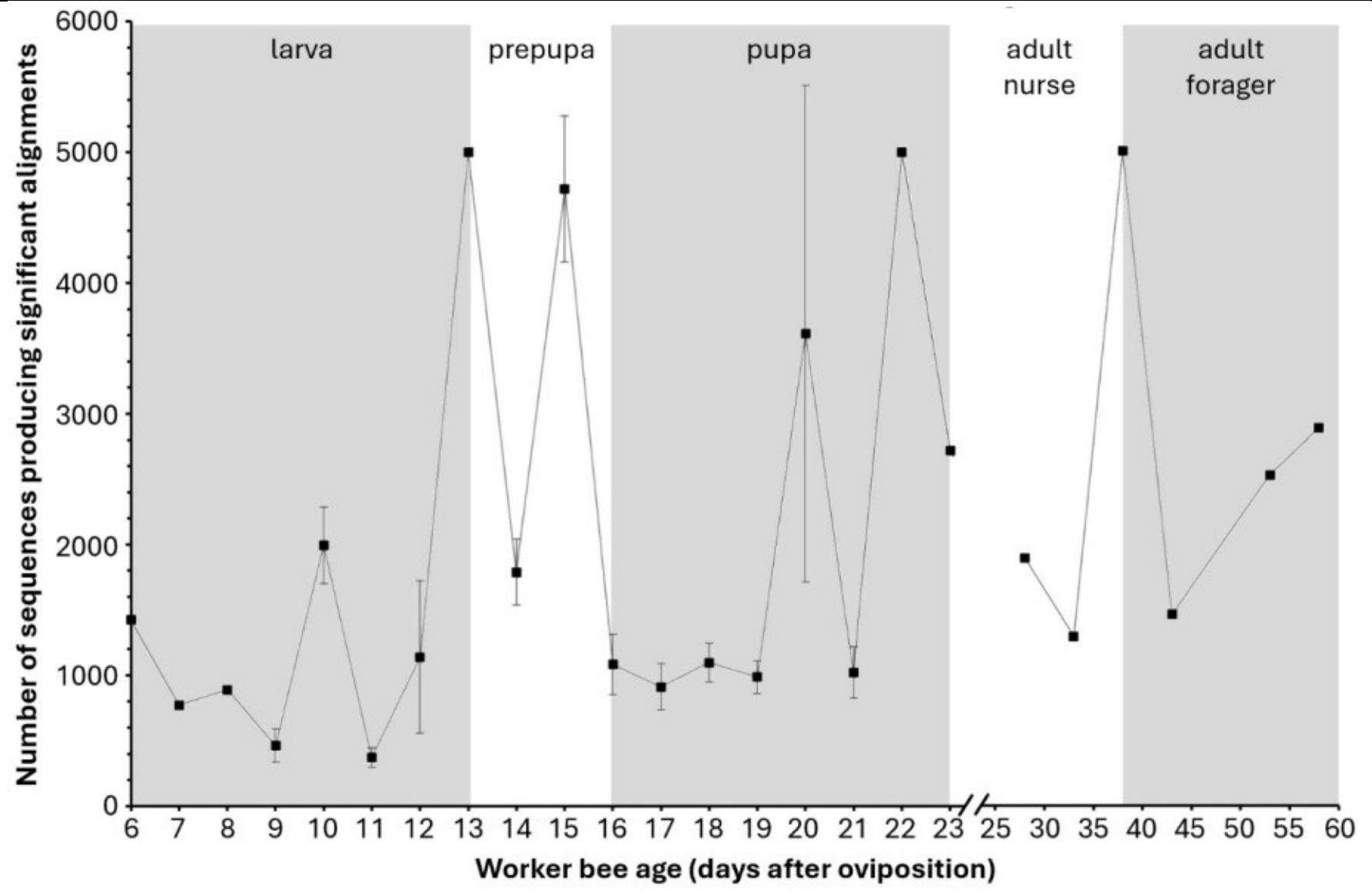

Figure S4: Numbers of sequences producing significant alignments against *mmp1*-isoform 2. The RNAseq data were made available online as part BioProject PRJDB14753 (Yokoi et al., 2025) in which an expression dataset for *A. mellifera* worker larva to adult development was published. The dataset comprises a single individual for days 6 - 8 and 23 - 58, two individuals for day 13, three individuals for days 12, four individuals for day 10, 14 and 15 and five individuals for days 9, 11 and 16 - 22. The graph shows means ± standard deviation for days with replicates.
